# Isolation, Identification, and Antibacterial Properties of Prodigiosin, a Bioactive Product Produced by a New *Serratia marcescens* JSSCPM1 Strain: Exploring the Biosynthetic Gene Clusters of *Serratia* Species for Biological Applications

**DOI:** 10.3390/antibiotics12091466

**Published:** 2023-09-20

**Authors:** Rajaguru Arivuselvam, Ayed A. Dera, Syed Parween Ali, Yasser Alraey, Ahmed Saif, Umme Hani, Sivaa Arumugam Ramakrishnan, Mohamed Sheik Tharik Abdul Azeeze, Raman Rajeshkumar, Aishwarya Susil, Haritha Harindranath, B. R. Prashantha Kumar

**Affiliations:** 1 Department of Pharmaceutical Biotechnology, JSS College of Pharmacy, JSS Academy of Higher Education & Research, Ooty 643001, TN, India; arajaguru@jssuni.edu.in (R.A.); siarvinayaga@gmail.com (S.A.R.); 2 Department of Pharmaceutical Biotechnology, JSS College of Pharmacy, JSS Academy of Higher Education & Research, Sri Shivarathreeshwara Nagar, Mysore 570015, KA, India; 3 Department of Clinical Laboratory Sciences, Central Research Laboratory, College of Applied Medical Sciences, King Khalid University, Abha 62529, Saudi Arabia; ayedd@kku.edu.sa (A.A.D.); sabali@kku.edu.sa (S.P.A.); yahamd@kku.edu.sa (Y.A.); 4 Department of Clinical Laboratory Sciences, College of Applied Medical Sciences, King Khalid University, Abha 62529, Saudi Arabia; amsaif8080@gmail.com; 5 Department of Pharmaceutics, College of Pharmacy, King Khalid University, Guraiger, Abha 62529, Saudi Arabia; uahmed@kku.edu.sa; 6 Department of Pharmaceutical Analysis, College of Pharmacy, JSS Academy of Technical Education, Noida 201301, UP, India; mtharik@jssaten.ac.in; 7 Department of Pharmaceutical Chemistry, JSS College of Pharmacy, JSS Academy of Higher Education & Research, Sri Shivarathreeshwara Nagar, Mysore 570015, KA, Indiaharithadd3@gmail.com (H.H.)

**Keywords:** *Serratia marcescens*, JSSCPM1 strain, prodigiosin, OmpF porin protein, antibacterial agent, in silico molecular docking

## Abstract

Prodigiosin pigment has high medicinal value, so exploring this compound is a top priority. This report presents a prodigiosin bioactive compound isolated from *Serratia marcescens* JSSCPM1, a new strain. The purification process of this compound involves the application of different chromatographic methods, including UV-visible spectroscopy, high-performance liquid chromatography (HPLC), and liquid chromatography–mass spectrometry (LC/MS). Subsequent analysis was performed using nuclear magnetic resonance (NMR) to achieve a deeper understanding of the compound’s structure. Finally, through a comprehensive review of the existing literature, the structural composition of the isolated bioactive compound was found to correspond to that of the well-known compound prodigiosin. The isolated prodigiosin compound was screened for antibacterial activity against both Gram-positive and Gram-negative bacteria. The compound inhibited the growth of Gram-negative bacterial strains compared with Gram-positive bacterial strains. It showed a maximum minimum inhibitory concentration against *Escherichia coli* NCIM 2065 at a 15.9 ± 0.31 μg/mL concentration. The potential binding capabilities between prodigiosin and the OmpF porin proteins (4GCS, 4GCP, and 4GCQ) were determined using in silico studies, which are generally the primary targets of different antibiotics. Comparative molecular docking analysis indicated that prodigiosin exhibits a good binding affinity toward these selected drug targets.

## 1. Introduction

The term antimicrobial resistance refers to the ability of microbes to withstand the inhibitory effects caused by antimicrobials. The efficacy of effective medications against these microbes diminishes as they develop this resistance [1]. These microbes have the ability to withstand effects caused by multiple medications, which is referred to as multidrug resistance (MDR). However, different resistance mechanisms have been seen in microbes, encompassing genetic mutations, naturally occurring resistance, and the acquisition of resistance patterns from other species through conjugation [2].

Universally, antimicrobial resistance is rising because of the reckless or careless use of antimicrobial medications. Treating resistant microbes poses significant challenges, necessitating the use of higher doses of medications or alternative choices of antimicrobials. However, most countries are negatively impacted because of shortages or a lack of effective medicines. According to the World Health Organization (WHO), multidrug-resistant pathogens, commonly referred to as “superbugs”, constitute a serious public threat and annually result in several million fatalities worldwide [3]. The WHO issued a priority list of pathogens resistant to antibiotics in 2021, focusing in particular on Gram-negative bacteria that exhibit high levels of resistance and pose a significant threat to human health [4]. The growing prevalence of antibacterial-resistant infections highlights the need for new antibacterial agents in modern medicine. Since the mid-20th century, considered the golden age of antibiotics, naturally derived compounds have played a vital role as potent therapeutic agents against pathogenic bacteria [5]. Given the lack of success in combinatorial approaches, we believe that focusing on the development of new antibiotics based on proven naturally derived compounds is the optimal short-term solution to combat the escalating crisis of antibiotic resistance.

The microbial secondary metabolite “prodigiosin” is a linear tripyrrol compound secreted by different bacteria, including *Streptomyces* spp., *Pseudomonas* spp., *Vibrio* spp., and *Serratia marcescens*, among others [6]. Prodigiosin (PG) belongs to the ‘prodiginine’ family and stands out as the most well-known member. Other members of this family include metacycloprodigiosin, cycloprodigiosin, undecylprodigiosin, streptorubin B, and prodigiosin R1 [7]. In recent years, healthcare professionals, scientists, and pharmaceutical companies have shown significant interest in the PG compound given its extraordinary medicinal value. This compound encompasses antibacterial [8,9,10,11,12,13,14], antifungal [15,16], anticancer [17,18,19,20,21], antiviral [22,23], and antimalarial [24,25] properties. According to the scientific literature, the PG compound shows significant antibacterial activity against Gram-negative bacteria, including *Klebsiella pneumoniae*, *Aeromonas hydrophila*, *Escherichia coli*, *Proteus mirabilis*, *Pseudomonas aeruginosa*, *Salmonella typhimurium*, *Proteus vulgaris*, and *Salmonella enteritidis* [9,10,11]. Furthermore, it shows activity against Gram-positive bacteria such as *Corynebacterium glutamicum*, *Enterococcus faecalis*, *Enterococcus faecium*, methicillin-resistant *Staphylococcus aureus*, *Listeria monocytogenes*, and *Bacillus cereus* [10,12,13,14]. The PG molecular mechanism of action against bacteria encompasses several general mechanisms. This includes interference with the cell cycle, DNA cleavage, pH disruption, phototoxicity, the generation of reactive oxygen species (ROS), and increased hydrophobic stress leading to plasma membrane disruption [13]. The impact of PG varies depending on whether the target bacteria are Gram-negative or Gram-positive. PG has been observed to cause lysis in Gram-positive bacterial cell walls, resulting in their demise. Conversely, in the case of Gram-negative bacteria, PG influences gene expression and protein synthesis, ultimately leading to alterations in the cellular life cycle and metabolism [9].

In this study, the bioactive compound PG was isolated from the *S. marcescens* JSSCPM1 strain and screened for antibacterial activity against both Gram-negative and Gram-positive bacteria. Analytical methods such as UV-visible spectroscopy, high-performance liquid chromatography (HPLC), liquid chromatography–mass spectrometry (LC-MS), and nuclear magnetic resonance (NMR) spectroscopic analysis were used to identify the purity and elucidate the structure of the PG bioactive compound. Additionally, the binding interaction between the ligand (PG bioactive compound) and the receptor OmpF porin protein (4GCS, 4GCP, and 4GCQ) was evaluated using in silico computational tools. By using the minimum inhibitory concentration method, we evaluated the antimicrobial properties of the PG compound against three Gram-negative (*E. coli* NCIM 2065, *K.pneumoniae* NCIM 2706, and *P.aeruginosa* NCIM 2036) and two Gram-positive bacterial strains (*Bacillus subtilis* NCIM 2545 and methicillin-resistant *Staphylococcus aureus* (MRSA) ATCC 43300). 

## 2. Results

### 2.1. Isolation of Red-Pigmented Bacteria and Biochemical Characterization

**Figure 1:**
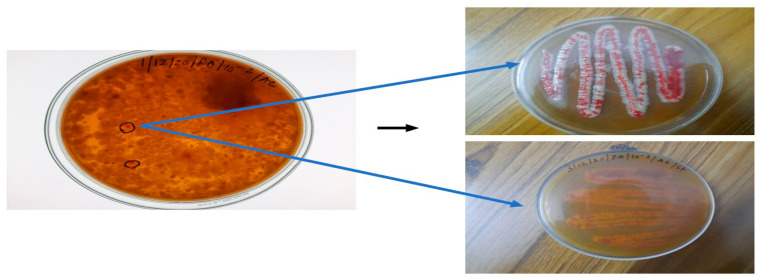
The blue-colored arrow represents the purified JSSCPM1 strain. This strain was selected for and subjected to further investigation.

Following the incubation period, the presence of red-pigment-producing colonies was observed in a 10^−2^ Petri dish. These colonies were taken with the help of an inoculation loop and subcultured in actinomyces isolation agar using a continuous streaking method to obtain pure red-pigmented, single-isolated colonies. Among the purely cultured colonies, the red-pigmented soil bacterium JSSCPM1 strain was selected for study (Figure 1).

This rod-shaped bacterium was motile, facultative anaerobe, and Gram-negative and optimally grew at 7 PH and 26 °C ± 2 °C in actinomyces isolation agar. The biochemical identification of the JSSCPM1 strain was positive for the indole production test; the Voges-Proskauert test; the Nitrate reduction test; and the catalase, urease, and glucose fermentation gas production test but negative for the methyl red test and the oxidase and acid production test (L-arabinose, D-xylose, and cotton sugar fermentation). However, the results of biochemical identification are shown in Supplementary File S1 Annexure 1, and specify that the biochemical characteristics of the JSSCPM1 strain are consistent with *S. marcescens*.

### 2.2. 16S rRNA Sequencing Report and Phylogenetic Tree Analysis

**Figure 2:**
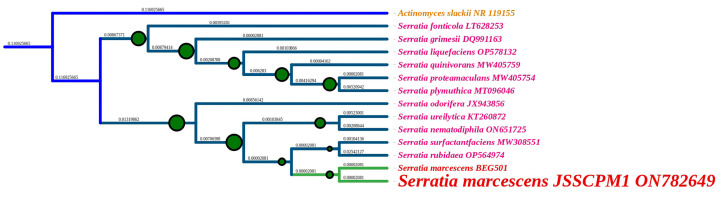
Phylogenetic tree of the *S. marcescens* JSSCPM1 strain and its twelve closest neighbors. A maximum likelihood phylogenetic tree was executed in MEGA 11 using 13 sequences with 1260 base pairs. The green-colored circle with black-colored borders in the branches indicates more than a 60% maximum likelihood of bootstrapping. The unique GenBank accession number is shown to the right of the species name. The dark blue-colored branch shows that *Actinomyces slackii* NR 119155 was used in the outgroup. A high-resolution phylogenetic tree PDF format is provided in Supplementary File S1 Annexure 2.

The molecular identification of bacteria is widely carried out using the 16S rRNA gene sequencing method [26,27]. This is because the 16S rRNA gene is present in every bacterium. It is significant that the function of the 16S rRNA gene has not altered throughout time and also provides nearly 1500 base pairs to identify the genera and species of bacteria [26]. Based on the biochemical identification test results, the JSSCPM1 strain was characterized as the *S. marcescens* species. Furthermore, the amplified partial sequence of this strain of JSSCPM1 16S rRNA genes was sequenced. The resulting sequence was subjected to BLAST analysis, and the results showed that the sequence has higher molecular similarities with the *S. marcescens* species. Thus, this sequence was identified as *S. marcescens* strain JSSCPM1. Furthermore, the sequence was published in the NCBI (USA) GenBank database under the accession number ON782649, with 1292 base pairs in terms of size. A voucher strain (*S. marcescens* strain JSSCPM1) was preserved at the Department of Pharmaceutical Biotechnology, JSS College of Pharmacy, Mysuru, Karnataka, India. A phylogenetic analysis of the *S. marcescens* JSSCPM1 strain shows that this strain is a member of the Serratia genus, closely clustering with the *S. marcescens* species, supported by a high bootstrap value (Figure 2).

### 2.3. Analyzing the PG Compound

The PG compound was collected from the 96 h cultured actinomyces isolation agar plates. A solubility test was conducted using polar solvents. This pigment shows a higher solubility rate in ethanol than in water. The yielded PG compound was too high and reached 1.5 g per liter.

**Figure 3:**
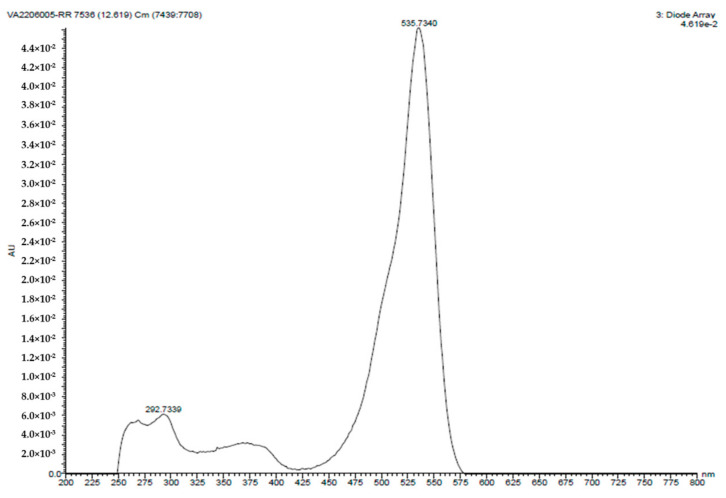
UV absorption spectrum of PG compound isolated from the *S. marcescens* JSSCPM1 strain. A high-resolution UV absorption spectra PDF format is provided in Supplementary File S1 Annexure 3.

The UV-visible spectroscopy analysis involved the PG solution being subjected to a UV-visible spectrophotometer (Shimadzu-1700) in a wavelength range of 200 to 800 nm. Figure 3 displays a distinct absorption peak at 535.73 nm, which corresponds to the characteristic peak of the PG compound.

**Figure 4:**
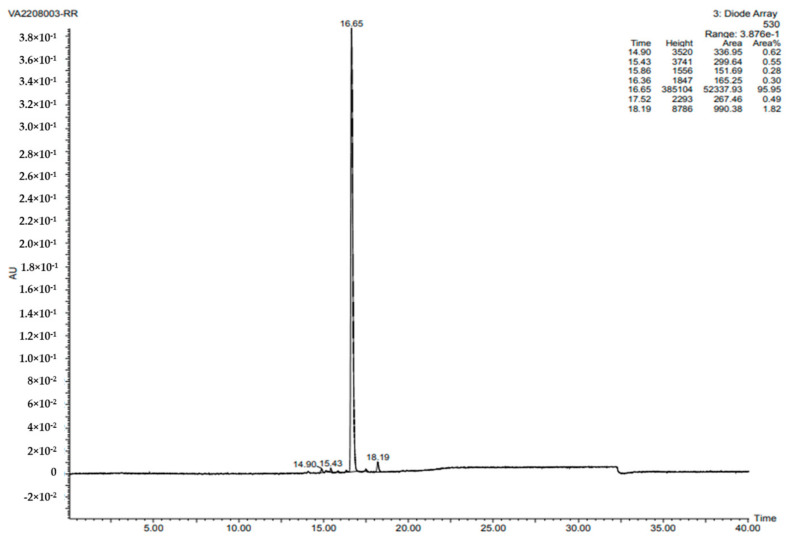
This figure shows the 95.95% purity of the PG compound isolated from the S. marcescens JSSCPM1 strain. A high-resolution HPLC report PDF format is provided in Supplementary File S1 Annexure 4.

To check the purity of the PG compound, an HPLC analysis was performed. The PG compound was observed to have 95.95% purity at a retention time of 16.65 min, and its maximum absorbance was detected at 535 nm (Figure 4). 

**Figure 5:**
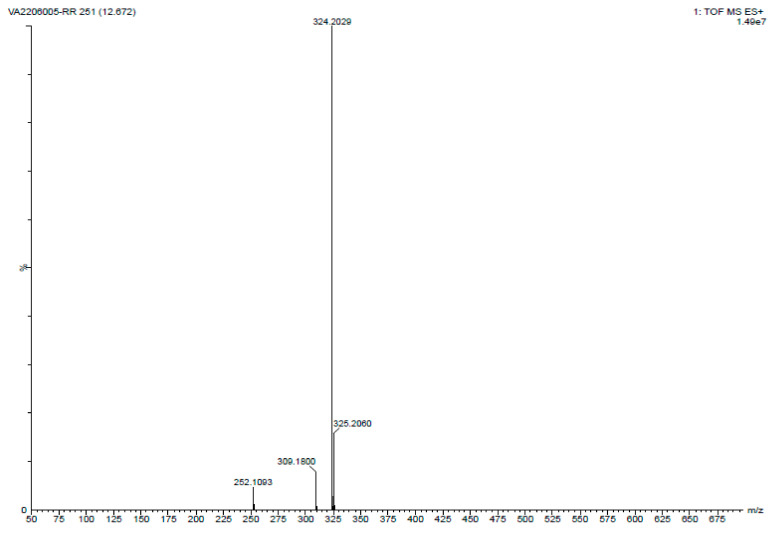
LC-MS analysis of PG compound isolated from the *S. marcescens* JSSCPM1 Strain. A high-resolution LC-MS report PDF format is provided in Supplementary File S1 Annexure 5.

The molecular weight of PG was determined using TOF MS ES+ mode. The following conditions were employed for the analysis: positive ion mode, a mass range of 100 to 1000 *m*/*z*, a nebulizer pressure of 20 psi, a drying gas flow rate of 10 L/min, a desolation gas temperature of 250 °C, and a capillary voltage of 3.5 kV. For the analysis, PG was dissolved in an ethanol solvent (ACN) and infused into the TOF MS instrument. The infused sample was ionized using an electrospray ionization source in positive ion mode. The ionized sample was then accelerated using a potential gradient and passed through a flight tube where it was separated based on its mass-to-charge ratio (*m*/*z*). The time-of-flight (TOF) of each ion was measured, and the resulting data were converted into a mass spectrum. The molecular weight of PG was calculated from the mass spectrum obtained using the TOF MS ES+ analysis. The molecular weight was found to be 323.43 (324.20 *m*/*z*), indicating that the compound had a relatively low molecular weight (Figure 5).

**Figure 6:**
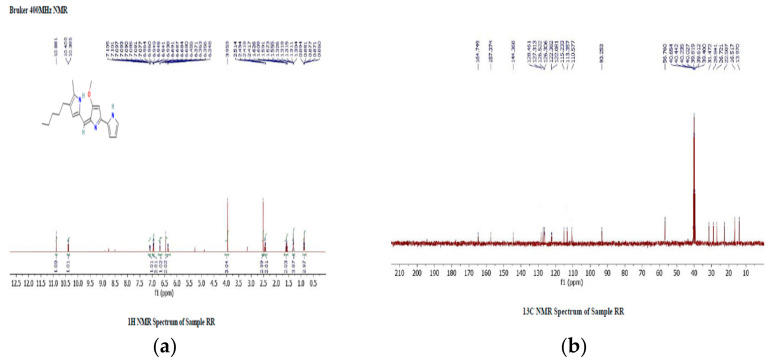
(**a**) 1H NMR report for PG compound isolated from the *S. marcescens* JSSCPM1 strain. A high-resolution 1H NMR report PDF format is provided in Supplementary File S1 Annexure 6. (**b**) 13C NMR report for PG compound isolated from the *S. marcescens* JSSCPM1 strain. A high-resolution 13C NMR report PDF format is provided in Supplementary File S1 Annexure 6.

1H Proton NMR spectroscopy (H-NMR) in CDCl3 solution reveals specific chemical shifts in the prodigiosin: peaks at 6.348–7.105 (five protons, multiplet, Ar–H, j = 7 Hz), 5.34 (one proton, singlet, =CH), 3.67 (three protons, singlet, OCH3), 2.32 (two protons, triplet, CH2), 2.01 (three protons, singlet, CH3), 1.31–1.21 (six protons, multiplet, CH2), and 0.86 (three protons, triplet, CH3). These chemical shifts indicate distinct hydrogen environments within the compound (Figure 6a). The 13C NMR spectrum of prodigiosin reveals 27 carbon signals, encompassing three sp3 carbons for methyl and methoxyl groups, six sp2 carbons (five aromatic and one olefinic carbon), and one CH from the imino ring (ring C). It also includes seven quaternary carbons and multiplets of 20 hydrogens (10 CH2) within δ 1.80 to ~0.95, along with methyl group triplets and a terminal methyl group triplet at δ 0.82 (J = 7.4 Hz) (Figure 6b).

This comprehensive analysis collectively leads to the confident conclusion that the compound is indeed “PG”, an outcome consistent with both MS and NMR data.

### 2.4. Molecular Docking Analysis

Molecular docking was performed using the PyRx-v0.8 virtual screening software with the Lamarckian genetic algorithm, and AutoDock Vina was utilized to study the interaction between PG (ligand) and OmpF porin proteins 4CGS, 4CGP, and 4CGQ. The best docking score was calculated, and the results were as follows: 4CGS = −6.8 kcal/mol, 4CGP = −6.9 kcal/mol, and 4CGQ = −7.1 kcal/mol. The molecular interaction between PG (ligand) and OmpF porin proteins 4CGS, 4CGP, and 4CGQ was analyzed using Discovery Studio 2021 (BIOVIA). 

**Figure 7:**
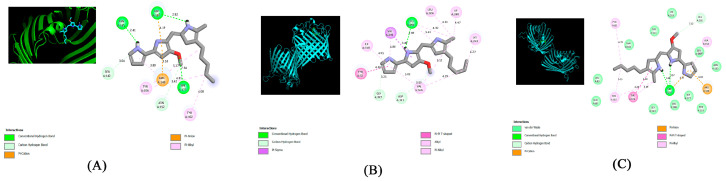
(**A***–***C**) Molecular interactions between the ligand PG and 4CGS-, 4CGP-, and 4CGQ-targeted proteins.

Figure 7A shows molecular interactions between the ligand PG and 4CGS-targeted proteins (Table 1), including three conventional hydrogen bonds (ASN141, ASP107, SER177), two carbon–hydrogen bonds (SER142, ASN152), one π-cation (ARG140), and two π -alkyls (TYR102, TYR106). 

Figure 7B shows molecular interactions between the ligand PG and 4CGP-targeted proteins (Table 1), including one conventional hydrogen bond (lEU291); two carbon–hydrogen bonds (GLY327, ASP121); one π-Σ bond and π-π T-shaped bond (VAL292 and TYR32, respectively); one alkyl (VAL326); and one π-alkyl (Leu324, ILE318, ILE240, lYS243).

Figure 7C shows molecular interactions between the ligand PG and 4CGQ-targeted proteins (Table 1), including one conventional hydrogen bond (ASP107), one carbon–hydrogen bond (GLU181), one π-cation and π-anion (ARG140), one π-π T-shaped bond (TYR106), and three π -alkyls (PHE85, TYR102, ALA154).

Molecular docking between the ligand PG and targeted porin proteins (4CGS, 4CGP, and 4CGQ) showed a strong binding affinity for the isolated red pigment. Using molecular docking software, we analyzed the properties of the isolated PG compound and found that it had the ability to work as an antibiotic agent by targeting the OmpF porin proteins. These findings suggest that the isolated PG may be a better alternative to other standard antibiotics that target OmpF porin proteins.

### 2.5. Antimicrobial Assay Evaluation Report

The PG compound’s antibacterial properties were evaluated with the broth microdilution method (CLSI 2007) using nutrient broth (Hi media). The minimum inhibitory concentration value of the PG compound is represented in Table 2 and compared with the standard drugs ampicillin and carbenicillin. PG exhibited excellent activity against *E. coli* NCIM 2065 with a MIC value of 15.9 μM, whereas the standard drugs ampicillin and carbenicillin showed MIC values of 5.6 μM and 4.1 μM, respectively. PG displayed significant activity against *K. pneumoniae* NCIM 2706 with a MIC value of 22.6 μM, while the standard drugs ampicillin and carbenicillin exhibited MIC values of 3.2 μM and 1.6 μM, respectively. PG showed average activity against *P. aeruginosa* NCIM 2036 with a MIC value of 46.1 μM, in comparison with the standard drugs ampicillin and carbenicillin, which had MIC values of 4 μM and 3 μM, respectively. PG exhibited average activity against *B. subtilis* NCIM 2545 with a MIC value of 43 μM, whereas the standard drugs ampicillin and carbenicillin demonstrated MIC values of 3 μM and 7 μM, respectively. PG displayed poor activity against *MRSA* ATCC 43300 NCIM 2706 with a MIC value of 73.6 μM, whereas the standard drugs ampicillin and carbenicillin showed MIC values of 19.8 μM and 13.1 μM, respectively. The PG compound shows that its activities were significantly lower in comparison with the standard drugs ampicillin and carbenicillin. However, the results indicate that the PG compound exhibited greater activity against Gram-negative bacteria compared with Gram-positive bacteria. This difference in activity against Gram-positive bacteria may be attributed to the poor penetration of the test compounds through the bacterial cell wall.

### 2.6. Biosynthetic Gene Cluster Prediction Report Analysis

The biosynthetic pathway of the PG compound is caused by the expression of the pig cluster. The pig cluster consists of a total of 14 genes and spans a length of 20,960 base pairs. In Sma 274, these genes are organized in a sequential manner, specifically as pigA to pigN [28], and the function mechanisms of these clusters are detailed in reference [29].

A total of 122 chromosomal genomes of *S. marcescens*, *Serratia* sp., and *Serratia nematodiphila* were used in this study, along with their name, GenBank accession number, and unique database links, which are represented in Supplementary File S1 Annexure 7. 

**Figure 8:**
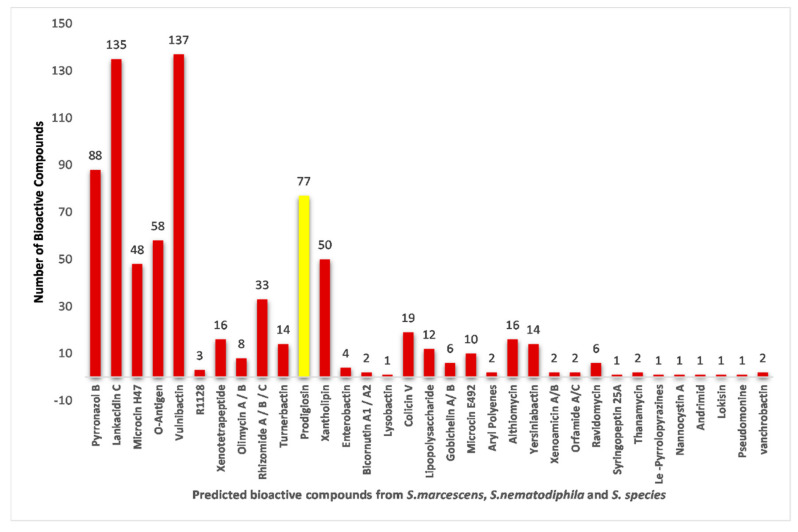
The total number of chromosomal genomes able to produce the PG compound.

**Figure 9:**
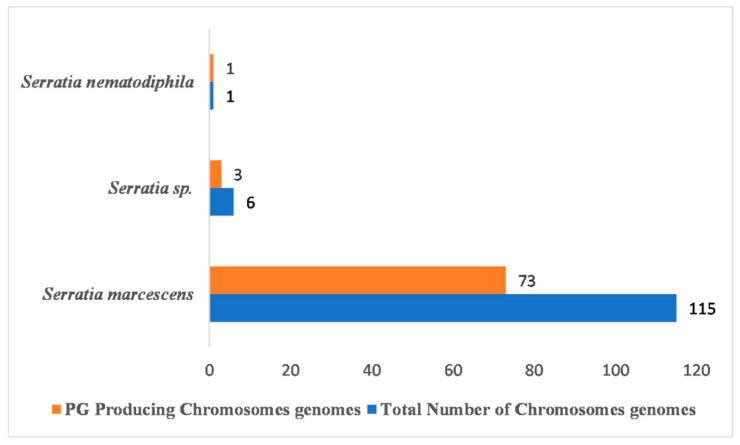
A total of 122 chromosomal genomes and 25 BGCs were found. Remarkably, these 25 clusters were found to occur in genomes multiple times, indicating their significance in various biological processes. These included 494 NRPSs, 68 RRE-containing, 115 thiopeptide, 121 betalactone, 21 hserlactone, 23 siderophore, 15 NRPS-like-hserlactone, 22 NRPS-T1PKS, 30 T1PKS-NRPS, 52 PG, 81 redox–cofactor, 1 thiopeptide-LAP, 1 NRPS-NRPS-like, 11 RiPP-like, 72 NRPS-like, 2 NRPS-like, NRPS, 6 thiopeptide-LAP-NRPS, 2 arylpolyene, 4 NRPS-prodigiosin, 1 NRPS-like-thiopeptide-LAP, 1 hserlactone-NRPS-like, 1 NRPS-like-arylpolyene, 2 T1PKS, 1 lanthipeptide-class-I, and 1 NRPS-butyrolactone gene clusters.

A comprehensive comparative analysis of PG-producing BGCs in 122 *Serratia species* belonging to three different categories (Supplementary File S1 Annexure 8) revealed that PG-producing BGCs are present in 77 Serratia species belonging to three different categories, namely, *S. marcescens*, *Serratia* sp., and *S. nematodiphila* (Figure 8 and Figure 9). Fascinatingly, not all *Serratia* species of *S. marcescens* and *Serratia* sp. categories have PG-producing BGCs (Figure 9). Among 122 Serratia species, only 77 *Serratia species* of *S. marcescens*, *Serratia* sp., and *Serratia nematodiphila* have PG-producing biosynthetic gene clusters; 73 of 115, 3 of 6, and 1 of 1 S. species of *S. marcescens*, *Serratia* sp., and *S. nematodiphila*, respectively, have this PG producing BGCs (Figure 9).

An AntiSMASH analysis revealed that a total of 122 chromosomal genomes have the potential to produce 33 bioactive compounds (Figure 8).

These compounds include Pyrronazol B, Lankacidin C, Microcin H47, O-Antigen, Vulnibactin, R1128, Xenotetrapeptide, Olimycin A/B, Rhizomide A/B/C, Turnerbactin, PG, Xantholipin, Enterobactin, Bicornutin A1/Bicornutin A2, Lysobactin, Colicin V, Lipopolysaccharide, Gobichelin A/Gobichelin B, Microcin E492, Aryl Polyenes, Althiomycin, Yersiniabactin, Xenoamicin A/B, Orfamide A/C, Ravidomycin, Syringopeptin 25A, Thanamycin, Le-Pyrrolopyrazines, Nannocystin A, Andrimid, Lokisin, Pseudomonine, and Vanchrobactin (Figure 7).

In this study, 122 chromosomal genomes revealed 25 BGCs (Figure 8). These gene clusters play a crucial role in the production of 33 bioactive compounds, and they encompass a diverse range of biosynthetic pathways. Among the identified clusters were NRPS, RRE-containing, thiopeptide, betalactone, hserlactone, siderophore, NRPS-like-hserlactone, NRPS-T1PKS, T1PKS-NRPS, PG, redox–cofactor, thiopeptide-LAP, NRPS-NRPS-like, RiPP-like, NRPS-like, NRPS-like, NRPS, thiopeptide-LAP-NRPS arylpolyene, NRPS-prodigiosin, NRPS-like-thiopeptide-LAP, hserlactone-NRPS-like, NRPS-like-arylpolyene, T1PKS, lanthipeptide-class-I, and NRPS-butyrolactone gene clusters.

**Figure 10:**
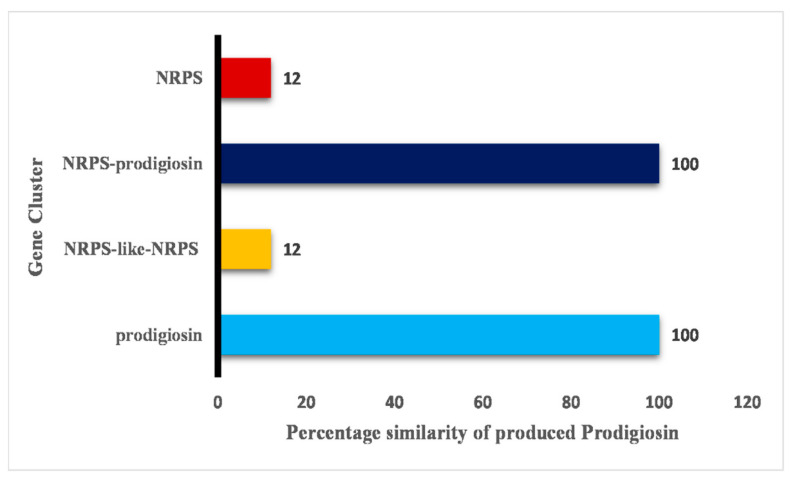
Different gene clusters responsible for the production of PG compound and its percentage similarities.

Among these 77 chromosomal genomes, different gene clusters are involved in the production of PG bioactive compounds, including PG, NRPS-like-NRPS, NRPS-prodigiosin, and NRPS. The gene clusters responsible for the production of PG bioactive compounds, namely, PG and NRPS-prodigiosin, exhibit 100% similarity to the PG compound. On the other hand, NRPS-like-NRPS and NRPS show only 12% similarity to the compound (Figure 10).

## 3. Discussion

Plants and microbes serve as the primary sources of natural pigments. Microbial-derived pigments offer several advantages over plant-derived pigments, including enhanced stability, cheap cost, excellent yields, and the convenience of downstream processing. These attributes make microbial pigments a favorable choice for synthetic pigments [30]. Nowadays, riboflavin [31], β-carotene [32], melanin [33], astaxanthin [34], and PG [35] are examples of natural pigments currently generated by microbes.

A significant number of PG-producing wild strains have been found in soil [36,37,38,39,40], freshwater lakes [41,42], and the ocean [43]. However, according to a literature survey, soil bacteria [36,37,38,39,40] play a promising role in producing the PG compound. In this study, we isolated the PG compound from the *S. marcescens* JSSCPM1 strain soil bacterium.

The composition of the medium plays an important role in the growth and development of bacteria. It is also responsible for the production of secondary metabolites. Different studies have suggested that peptone glycol medium [6,40,44,45,46], nutrient broth [8,47,48], and lysogeny broth medium [49] are employed most often for producing PG. We used actinomyces isolation agar and actinomyces broth for the isolation of the PG compound. According to Tao J. et al., carbon sources (glycerol) are essential for the production of the PG compound [50]. Thus, we chose actinomyces isolation agar, which is rich in carbon sources. In actinomyces isolation agar, glycerol acts as a carbon source, and this helps in the production of the PG compound.

*S. marcescens* was the first species to produce the PG compound [51]. According to research in the literature, PG is produced by bacteria in different amounts. However, we achieved a PG compound yield of 1.5 g per liter. De Araújo H.W. et al. [36] isolated *S. marcescens* UCP 1549 from semiarid soil and achieved the highest yield of PG compound at around 49.5 g per liter [36]. 

Utilizing UV-visible spectroscopy, HPLC, LC-MS, and NMR methods, a qualitative analysis of the PG compound was performed. UV-visible spectroscopy shows that the maximum absorption of the PG compound dissolved in methanol varies between 460 and 540 nm [41,52]. This absorption value differs based on the solvent pH utilized. For example, PG maintains its red color at pH 7.0, and it shows maximum absorption at 533 nm. At pH 2.0, PG changes its color to pink and shows maximum absorption at 540 nm. Similarly, at pH 9.0, PG changes its color to orange and shows maximum absorption at 468 nm [40]. In this study, the UV-visible spectroscopy maximum absorption of the isolated PG compound was 535.73 nm, which corresponds to the characteristic peak of the PG compound. Determining the purity of the PG compound is usually achieved by using the HPLC method. This method offers both qualitative and quantitative assessments of results. We achieved 95.95% purity at a retention time of 16.65 min. Nguyen S. et al. isolated the PG compound from *S. marcescens* EMS and achieved 98.67% purity at an initiated retention time of 18.866 min [53]. In another study, Lee JS. et al. isolated a PG compound from *Zooshikella rubidus* S1-1 and determined its purity at a retention time of 7.86 min [54]. When utilizing HPLC for analysis, these investigations revealed variance in the retention time. In our study, the determination of the molecular weight of the PG compound was performed using the LC-MS method. The LC-MS results showed a single peak of 324.20 *m/z*, which corresponds to the molecular weight of PG from the study conducted by Lin C. et al. [38] and Nguyen S. et al. [53]. The structure of the PG compound was determined through the use of high-field 13C-NMR and 1H-NMR spectroscopy. The structure of the PG compound was identical to the PG compound obtained from various other studies (for example, references [53,55,56]). 

Normally, cyclic molecules typically show prominent differences in antibacterial activity when compared with linear molecules [54]. Kamble K.D. et al. [8] proposed three potential mechanisms through which PG functions as an effective antimicrobial agent: firstly, by cleaving bacterial DNA; secondly, by inhibiting the cell cycle; and thirdly, by modulating pH levels [8]. The outer membrane (OM) of Gram-negative bacteria serves as the initial defense mechanism, safeguarding cells from various environmental challenges, such as physical, chemical, and biological threats [57]. At the same time, it facilitates the specific absorption of vital nutrients and the discharge of metabolic byproducts. In terms of its biochemical arrangement and makeup, the OM is a complex arrangement of lipids and proteins. The OM consists of an uneven lipid bilayer where phospholipids exclusively reside in the inner leaflet, while the outer leaflet is enveloped by lipopolysaccharides [58].

The OM also comprises proteins known as outer membrane proteins (OMPs), specifically referred to as porins. These porins possess a characteristic β-barrel structure, forming aqueous channels that facilitate the passage of various hydrophilic molecules. Among the antibiotics that exhibit activity against Gram-negative bacteria, two prominent classes are β-lactams and fluoroquinolones. These antibiotics are relatively small and hydrophilic, allowing them to bind and disrupt the functioning of OmpF porin proteins [59].

In our research, we focused on the interaction between traditional antibiotics and OmpF porin proteins, utilizing structures such as 4CGS, 4CGP, and 4CGQ as examples. By using molecular docking, we examined the PG compound’s antibacterial activity by targeting OmpF porin proteins, and the results show that it has the capacity to bind OmpF porin proteins analogous to where antibiotic drugs commonly bind, signifying its functional resemblance to commercially available medications.

In this study, PG exhibited effective action against Gram-negative bacterial strains of *E. coli* NCIM 2065, *K. pneumoniae* NCIM 2706, and *P. aeruginosa* NCIM 2036. In contrast, PG exhibited poor activity against *B. subtilis* NCIM 2545 and *MRSA* ATCC 43300. However, Darshan N. et al. [40] found that PG derived from *S. nematodiphila* triggers programmed cell death (PCD) in *P. aeruginosa*, *E. coli*, *B. cereus*, and *S. aureus*. Additionally, their study found that PG inhibits the motility of *B. cereus* and *E. coli* [40]. However, PG has been shown to display inhibition effects against a range of microorganisms, including *B. cereus*, *S. aureus* [45], *B. licheniformis*, *A. tumefacien*, *A. anitratus*, *MSRA*, *Micrococcus* sp., *E. coli*, *Erwinia* sp., *B. thuringiensis*, *S. saprophyticus*, and *S. epidermidis* [60]. 

An AntiSMASH analysis of 122 chromosomal genomes revealed that each *Serratia species*, falling into three different categories, has gene clusters responsible for producing bioactive compounds, such as Vulnibactin, Pyrronazol B, and Lankacidin C, in comparison with the gene clusters responsible for producing PG (Supplementary File S1 Annexure 8 and Figure 1). However, our search using the PubMed search engine resulted in a limited number of studies, with 25 on Vulnibactin, 3 on Pyrronazol B, and 26 on Lankacidin C (as of 07/2023).

On the other hand, only a few chromosome genome gene clusters of the *Serratia* species are responsible for producing bioactive compounds such as R1128, Olimycin A/B, Enterobactin, Bicornutin A1/Bicornutin A2, Lysobactin, Gobichelin A/Gobichelin B, Aryl Polyenes, Xenoamicin A/B, Syringopeptin 25A, Thanamycin, Le-Pyrrolopyrazines, Nannocystin A, Andrimid, Lokisin, Pseudomonine, and Vanchrobactin (Supplementary File S1 Annexure 8 and Figure 7).

It may be that BGCs responsible for these compounds are cryptic in nature, which has led researchers to focus on accessing these compounds given their high medical value and potential impact on antimicrobial resistance treatment.

## 4. Experimental Section

### 4.1. Isolation and Purification of Soil Bacteria

The study area was Ooty, a city in the Nilgiris district of the South Indian province of Tamil Nadu. The Nilgiris district is a natural corridor linking Kerala and Karnataka. Ooty is a mountainous area bordered to the northwest by Coimbatore and south by Mysore. The soil sample was collected from HMT Street of Ooty in a sterile polyethene container. The sample was obtained from a 12 to 15 cm depth using an ax. After collection, the soil sample was transferred to the laboratory for processing. Then, 1 g of soil sample was added to a 250 mL conical flask with 100 mL of demineralized water, and uniform mixing at 400 rpm at 26 °C ± 2 °C for 2 h an using orbital shaker (LT-Orbital Laboratory Incubator Shaker) was conducted. After that, 1 mL of soil solution was taken and serially diluted in demineralized water using a ten-fold serial dilution method down to 10^−10^. In total, 100 µL from each was evenly spread with a sterile L-shaped glass rod on the surface of actinomyces isolation agar plates using the spread plate technique. All the Petri dishes were incubated at 26 °C ± 2 °C for 96 h, and colony formation was observed [61,62,63,64,65]. 

The microscopic examination and Gram-staining were performed on the JSSCPM1 strain [66,67]. Moreover, the biochemical identification of the JSSCPM1 strain was performed based on the book *Berger Bacteria Identification* [68].

### 4.2. Molecular Characterization

#### 4.2.1. Isolation of JSSCPM1 Strain Genomic DNA 

The taxonomical identification of the *S. marcescens* strain JSSCPM1 was performed using 16S rRNA gene sequencing. The JSSCPM1 genomic DNA was extracted using the MEDOX-Easy Ultrapure Genomic DNA Spin Minipreps Kit (Bacteria) according to the manufacturer’s instructions.

#### 4.2.2. 16S rRNA Gene Sequencing of JSSCPM1 Strain

16S rRNA gene amplification of the isolate JSSCPM1 strain was carried out using a personal PCR (Eppendorf^®^ Mastercycler, Hamburg, Germany; personal; AC/DC input, 230 V; AC, 50–60 Hz) with the help of 27F (forward primer) and 1492R (reverse primer) using Taq polymerase enzymes [62,69,70]. After that, the amplified PCR products were verified using 0.7% agarose gel with a 2D gel electrophoresis system (Gel Electrophoresis Unit (Horizontal) (Model No. HV-1)), and the gene product was purified and sequenced using a DNA analyzer [71]. The resulting sequences were subjected to the NCBI (National Center for Biotechnology Information) nucleotide BLAST (Basic Local Alignment Search Tool) program with 1000 targeted sequences [66] to analyze the similarity, and they were deposited in GenBank with a unique accession number. 

#### 4.2.3. Phylogenetic Tree Construction 

To construct a phylogenetic tree, the sequence of the *S. marcescens* JSSCPM1 strain and its twelve closest-neighbor sequences were retrieved from the NCBI using a nucleotide search. Multiple sequence alignments of these sequences were carried out using the MUSCLE algorithm, executed with the MEGA11 software [72]. The phylogenetic tree was constructed using the maximum likelihood method with the Kimura two-parameter model, and the resulting tree phylogeny was evaluated using bootstrap analysis with 500 replicates [73,74]. The generated tree was created and visualized on the iTOL server (Interactive Tree of Life) [75].

### 4.3. Pigment Extraction and Characterization

A purely cultured *S. marcescens* JSSCPM1 strain was inoculated in 20 mL of actinomyces broth, incubated at 26 °C ± 2 °C for 48 h, and then transferred into 500 mL of actinomyces broth and incubated for a further 26 °C ± 2 °C for 48 h. Then, 100 µL from this cultured broth was spread onto actinomycetes isolation agar plates and incubated at 26 °C ± 2 °C for 96 h. The lawn of the incubated plates was dissolved in ethanol and centrifuged for 30 min at 5000 rpm using a cooling centrifuge (Remi CM-12 Plus 8 × 5 mL Cooling Micro Centrifuge with Angle Rotor Head). After centrifugation, the supernatant layer was collected and dried at 37 °C using a bacteriological incubator [76]. The resulting dried red pigment (PG compound) was used for further study.

#### 4.3.1. UV-Visible Spectroscopic Analysis

The PG solution was prepared using 1 mg of dried PG diluted with a concentration of 95% ethanol (Merck). From this stock solution, a 1 µg/mL solution was prepared by diluting 1 mL of the stock solution with 10 mL of ethanol, and its absorption spectrum in the range of 200–800 nm was analyzed using a UV-1700 spectrophotometer (Shimadzu) [77] at the Department of Pharmaceutical Analysis, JSS College of Pharmacy, Mysuru.

#### 4.3.2. HPLC Analysis

The red pigment was subjected to HPLC analysis at the Department of Pharmaceutical Analysis, JSS College of Pharmacy, Mysuru, in order to obtain purified fractions. The purity of the red pigment was checked using an HPLC system (Shimadzu, Kyoto, Japan) equipped with a diode-array UV detector (SPD-M10A) and with an analytical column (Jones Chromatography C18, 150 mm × 4.6 mm) of 3 µm in particle size. Firstly, the PG compound was dissolved in HPLC-grade acetonitrile (Merck Limited (Mumbai, India)) at a ratio of 70:30 (*v*/*v*) into a 10 mg/mL concentration, and 20 µL was injected into an injector. This PG compound passed into the Jones Chromatography column along with mobile phases at ambient temperatures. Elution was performed with 0.1% HPLC-grade formic acid (Merck Limited (Mumbai, India)) as solvent A [78] and 0.1% HPLC-grade acetonitrile (Merck Limited (Mumbai, India)) as solvent B [78], with a flow rate of 3 mL/min a detection wavelength of 535 nm [52], with parameters of 0 to 20 min. As pure PG standards can be cost-prohibitive, the resultant HPLC chromatogram was compared with a reference chromatogram reported by Lee M.A. et al. [79] and Lin P.B. et al. [76]. 

#### 4.3.3. LC-MS Analysis

The molecular weight of the PG was determined using a Shimadzu 8030 LC system with tandem mass spectrometry in Tokyo, Japan. The LC system included an LC-20AD pump, a SIL-20AC autosampler, a CBM-20 Alite controller, a CTO-20AC column oven, and an SPD-M20 PDA detector [38,80]. 

#### 4.3.4. 1H and 13C NMR Spectroscopic Analysis

The structural analysis of the PG compound was performed using 13C NMR and 1H NMR [53] at the University of Mysore, India. The NMR spectra, both 1D and 2D, were acquired using a Bruker AV 500 MHz spectrometer. The measurements were performed in CDCl3 solvent. For 1H NMR analysis, a frequency of 500 MHz was used, while for 13C NMR, a frequency of 125 MHz was utilized. In both cases, tetramethylsilane (TMS) served as the internal standard for referencing the chemical shifts. The NMR signals obtained were classified based on their appearance in the spectra, such as singlet (s), doublet (d), double doublet (dd), triplet (t), quintet (quint), and multiplet (m), providing information about the multiplicity and splitting patterns of the respective proton or carbon nuclei.

### 4.4. Molecular Docking Process—Experimental Protocol

The roadmap for this investigation involved selecting and preparing various protein targets, retrieving ligand molecules, and executing molecular docking. The RCSB-PDB (https://www.rcsb.org/, accessed on 27 August 2023) and PubChem (https://pubchem.ncbi.nlm.nih.gov/, accessed on 27 August 2023) databases were used to find and retrieve the 3D crystal structures of the target proteins and ligand molecules. The PyRx-v0.8 software [81], featuring AutoDock Vina [82], was used to determine the binding affinities between the protein targets and ligands. Finally, molecular interactions were analyzed using the Discovery Studio 2021 (BIOVIA) software [83].

#### 4.4.1. Preparation of Protein Targets

The structures of the OmpF porin proteins were selected for interaction with the isolated PG. The crystal structures of *E. coli* OmpF porin were in complexes with Ertapenem (PDB ID: 4GCS) (https://www.rcsb.org/structure/4GCS, accessed on 26 August 2023), Ampicillin (PDB ID: 4GCP) (https://www.rcsb.org/structure/4GCP, accessed on 26 August 2023), and Carbenicillin (PDB ID: 4GCQ) (https://www.rcsb.org/structure/4GCQ, accessed on 26 August 2023), which were retrieved from the RCSB database in PDB format [84]. The structures of 4GCS, 4GCP, and 4GCQ showed reasonable resolutions at 1.87 Å, 1.98 Å, and 2.20 Å, respectively. The preparation and refinement of the porin protein complexes, including the removal of water molecules, native ligands, and heteroatoms, were executed using the Swiss PDB Viewer software [85].

#### 4.4.2. Preparation of Ligand

The structure of the isolated PG (https://pubchem.ncbi.nlm.nih.gov/compound/135455579, accessed on 26 August 2023) was retrieved in SDF format from PubChem and converted to the Autodock-suitable PDBQT format in PyRx-v0.8. Finally, the ligand was energy-minimized using the Universal Forcefield (UFF) of PyRx-v0.8.

#### 4.4.3. Molecular Docking

The molecular docking investigation was conducted using the PyRx-v0.8 virtual screening software, which featured AutoDock Vina with the Lamarckian genetic algorithm [86,87,88,89]. The active sites for the target proteins, 4CGS (Tyr102, Gly15, Arg140, Lys46, Lys16, Tyr14, Tyr58, Lys89, Asn101, Gly110, Gln60, Ser95, Phe85, Asp97, Asp107, Phe340, Tyr106, Gln339), 4CGP (Tyr294, Arg42, Leu291, Asn316, Tyr32, Arg82, Glu117, Val326, Gly119, Ala123, Arg132, Tyr124, Pro239, Arg167, Ile240, Asp121, Asp113, Val292, Phe118, Gly120, Asn293, Thr241, Ile318, Gly327), and 4CGQ (Leu115, Leu83, Tyr106, Asp107, Gly110, Tyr111, Met114, Arg140, Asn152, Ser177, Ser179, Glu181, Lys16, Val188, Lys219, Gln262, Asp113, Tyr102, Pro116, Arg270, Arg82, Thr300, Glu62, Tyr302) were identified through an extensive literature review [84,90] and were used in the docking approach. The dimensions of the grid box were defined based on the size and shape of the active sites, facilitating efficient execution of the docking approach. Ligands were docked with their respective target proteins, and the results were clustered based on the root-mean-square deviation (RMSD) criterion. Ligands with 0 RMSD were selected as potential lead compounds for drug discovery. The interactions between the selected ligands and the target proteins were determined using PyMol [83].

#### 4.4.4. Protein–Ligand Interactions

At the end of the docking process, Discovery Studio 2021 (BIOVIA) [83] was used to generate 2D diagrams for each “protein–ligand complex” to identify the amino acid residues involved in important molecular interactions and significant contacts that secure the ligand within the active site.

### 4.5. Antimicrobial Activity

The PG compound’s antibacterial properties were evaluated against three Gram-negative (*E. coli* NCIM 2065, *K. pneumoniae* NCIM 2706, *P. aeruginosa* NCIM 2036) and two Gram-positive bacterial strains (*B. subtilis* NCIM 2545, *MRSA* ATCC 43300) using the guidelines of Clinical & Laboratory Standards Institute (CLSI 2007). The entire set of tests was accomplished using the broth microdilution method in nutrient broth (Hi-media), utilizing 96-well microtiter plates. The PG compound was dissolved in ethanol and was screened for its antibacterial activity in a concentration range of 12.5 to 100 μg/mL. Ampicillin and carbenicillin were used as positive controls dissolved in ethanol, while ethanol alone was used as a negative control. Bacterial suspensions at 10^5^ (CFU/mL) were inoculated into the corresponding wells along with various concentrations of PG compound and positive controls. Subsequently, the 96-well microtiter plates were incubated without agitation at 36 °C for 24 h. Following the incubation period, the plates were agitated, and the absorbance was measured at 530 nm [91]. The tests were performed in triplicate, and the results were recorded as the mean value. 

The following formula was used to compute the viability percentage:

Percentage viability = (OD control − OD sample/OD control) × 100

The percentage inhibition was calculated using this formula (100-cell viability) [92]. Typically, a MIC ratio less than or equal to two is classified as being indicative of bactericidal activity for a test compound, signifying its ability to kill bacteria effectively. Conversely, a MIC ratio greater than or equal to four is regarded as an indication of bacteriostatic activity, meaning that the test compound is capable of inhibiting bacterial growth without necessarily killing the bacteria outright [91,93].

### 4.6. Biosynthetic Gene Cluster Prediction

AntiSMASH (Antibiotics and Secondary Metabolites Analysis Shell) [94] is a commonly used tool to detect and analyze secondary-metabolite-producing biosynthetic gene clusters (BGCs) in the genomes of bacteria and fungi [95,96]. In this study, 1715 whole-community metagenome assemblies generated from *S. marcescens* were retrieved from the NCBI Assembly Database [97] (as of 11 December 2022). To limit the analyzed dataset, fully assembled 165 chromosomal genomes were extracted from the assemblies. To further narrow down the analyzed dataset, the BLAST (described in method 2.2.2) results showed that the 122 chromosomal genomes had molecular similarities with *S. marcescens* strain JSSCPM1. Thus, out of 165, 122 chromosomal genomes were selected for further study. The 122 GenBank files of the *S. marcescens* chromosomal genomes were mined to analyze the possible secondary-metabolite-producing BGCs using AntiSMASH 6.0 with default features [95,97]. 

## 5. Conclusions

The pigment extracted from the *S. marcescens* JSSCPM1 strain is identical to PG, consistent with the findings reported in the existing literature. PG exhibited significant antibacterial efficacy against Gram-negative bacterial strains when compared with its effects on Gram-positive bacterial strains. The sensitivity of Gram-negative bacterial strains *E. coli* NCIM 2065, *K. pneumoniae* NCIM 2706, and *P. aeruginosa* NCIM 2036 reveals the moderate effective action of PG in comparison with the usual antibiotics.

However, molecular docking studies revealed PG has the ability to bind OmpF porin proteins, mirroring the mechanisms of action of commercially available antibiotics. This discovery suggests that PG could serve as a functional alternative to conventional antibiotics, offering a temporary solution to combat antibiotic-resistant bacteria.

Moreover, our AntiSMASH analysis of chromosomal genomes revealed intriguing insights into the gene clusters responsible for producing bioactive compounds in *Serratia* species. 

While PG is a valuable compound, this study highlights how *Serratia* species possess a diverse array of gene clusters responsible for producing other bioactive compounds with varying degrees of research attention. This underscores the complexity of secondary microbial metabolites and their potential contributions to addressing antimicrobial resistance.

We found nearly 998 studies (as of 7 September 2023) by using the keyword prodigiosin, but we found a limited number of studies on certain bioactive compounds produced by *Serratia* species, for example, Vulnibactin, Pyrronazol B, and Lankacidin C, which underscores the need for further exploration in these areas. The cryptic nature of *Serratia* species biosynthetic gene clusters (BGCs) associated with these compounds presents a tantalizing challenge for researchers. Unlocking the potential of these cryptic BGCs could have a profound impact on the field of antimicrobial resistance treatment and the development of novel therapeutics.

While many researchers have traditionally focused their efforts on the natural occurrence of PG, it has already been explored for various potential applications, so a shift in research emphasis is imperative. The emergence of antimicrobial resistance underscores the need to redirect attention toward cryptic gene clusters. Researchers should explore these gene clusters using innovative techniques such as co-culturing, nutritional manipulation, and gene editing to stimulate the discovery of novel bioactive compounds. In light of the urgent challenges posed by antimicrobial resistance, a fresh approach centered on cryptic gene clusters holds the promise of uncovering innovative solutions.

Finally, the existing literature highlights the potential of PG as a versatile compound with promising antiviral, antibacterial, antifungal, and immunosuppressant properties. However, for PG to transition from laboratory promise to practical clinical applications, a comprehensive and rigorous drug development process is required. This process encompasses safety and toxicology studies, preclinical research to understand its mechanisms of action, formulation development, regulatory approval, and clinical trials involving different phases. Additionally, scaling up production, addressing market competition, securing funding, and adhering to ethical and regulatory standards are vital considerations. While PG holds significant potential, its journey from scientific discovery to a clinically approved compound demands careful, multidisciplinary collaboration and adherence to stringent regulatory and ethical guidelines to ensure its ultimate success in the medical field.

## Figures and Tables

**Table 1:** Molecular interactions between the ligand PG and OmpF porin proteins (4GCS, 4GCP, and 4GCQ).

Ligand	Protein PDB ID	Conventional Hydrogen Bond	Carbon–Hydrogen Bond	Pi-Cation	Pi-Sigma	Pi-Pi T-Shaped	PI-ANION	Alkyl	Pi-Alkyl
PG	4CGS	ASN141, ASP107, SER177	SER142, ASN152	ARG140	-	-	ARG140	-	TYR102, TYR106
	4CGP	lEU291	GLY327, ASP121	-	VAL292	TYR32	-	VAL326	Leu324, ILE318, ILE240, lYS243
	4CGQ	ASP107	GLU181	ARG140	-	TYR106	ARG140	-	PHE85, TYR102, ALA154

**Table 2:** PG compound antibacterial properties were evaluated against selected Gram-negative and Gram-positive bacteria.

Compound	Minimum Inhibitory Concentration of PG Compound (μg/mL)				
	*E. coli* NCIM 2065	*K. pneumoniae* NCIM 2706	*P. aeruginosa* NCIM 2036	*B. subtilis* NCIM 2545	*MRSA* ATCC 43300
PG	15.9 ± 0.31	22.6 ± 0.08	46.1 ± 0.5	43 ± 0.49	73.6 ± 0.16
Ampicillin	5.6 ± 0.72	3.2 ± 0.60	4 ± 0.26	3 ± 0.53	19.8 ± 0.32
Carbenicillin	4.1 ± 0.58	1.6 ± 0.77	3 ± 0.68	7 ± 0.08	13.1 ± 0.98

## Data Availability

Data will be provided on request.

## Supplementary Materials

The following supporting information can be downloaded at https://www.mdpi.com/article/10.3390/antibiotics12091466/s1: The supplementary material includes all supporting analyses and analytical data. Supplementary File S1, Annexure 1: Biochemical test identification results of JSSCPM1 strain. Annexure 2: Phylogenetic tree of *S. marcescens* JSSCPM1 strain and its twelve closest neighbours. Annexure 3: UV absorption spectrum of PG compound. Annexure 4: HPLC report of PG compound. Annexure 5: LC-MS analysis of PG Compound. Annexure 6: ^1^H and ^13^C NMR report of PG compound. Annexure 7: Total 122 chromosomal genomes of *S. marcescens*, *Serratia* sp., and *Serratia nematodiphila* used in this study, along with their name, GenBank accession number and unique database links. Annexure 8: Comprehensive comparative analysis of PG producing BGCs in 122 *Serratia* species belonging to three different categories.
