# Supplementary material for: Isolation, Identification, and Antibacterial Properties of Prodigiosin, a Bioactive Product Produced by a New Serratia marcescens JSSCPM1 Strain: Exploring the Biosynthetic Gene Clusters of Serratia Species for Biological Applications
Source: Antibiotics (Basel). 2023 Sep 20;12(9):1466. doi: 10.3390/antibiotics12091466 (PMC10526024; doi:10.3390/antibiotics12091466)
Supplement: Supplementary file 1 [file antibiotics-12-01466-s001.zip › antibiotics-2605368-supplementary.pdf]

# Isolation, Identification, and Antibacterial Properties of Prodigiosin, a Bioactive Product Produced by a New *Serratia marcescens* JSSCPM1 Strain: Exploring the Biosynthetic Gene Clusters of *Serratia* Species for Biological Applications

**Rajaguru Arivuselvam [1,2], Ayed A Dera [3], Syed Parween Ali [3], Yasser Alraey [3], Ahmed Saif [4], Umme Hani [5], Sivaa Arumugam Ramakrishnan [1,2], Mohamed Sheik Tharik Abdul Azeeze [6], Raman Rajeshkumar [1,*], Aishwarya S [7], Haritha H [7] and B.R. Prashantha Kumar [7,*]**

[1] Department of Pharmaceutical Biotechnology, JSS College of Pharmacy, JSS Academy of Higher Education & Research, Ooty 643001, TN, India; arajaguru@jssuni.edu.in (R.A.); siarvinayaga@gmail.com (S.A.R.)

[2] Department of Pharmaceutical Biotechnology, JSS College of Pharmacy, JSS Academy of Higher Education & Research, Sri Shivarathreeshwara Nagar, Mysore 570015, KA, India

[3] Department of Clinical Laboratory Sciences, Central Research Laboratory, College of Applied Medical Sciences, King Khalid University, Abha 62529, Saudi Arabia; ayedd@kku.edu.sa (A.A.D.); sabali@kku.edu.sa (S.P.A.); yahamd@kku.edu.sa (Y.A.)

[4] Department of Clinical Laboratory Sciences, College of Applied Medical Sciences, King Khalid University, Abha 62529, Saudi Arabia; amsaif8080@gmail.com

[5] Department of Pharmaceutics, College of Pharmacy, King Khalid University, Guraiger, Abha 62529, Saudi Arabia; uahmed@kku.edu.sa

[6] Department of Pharmaceutical Analysis, College of Pharmacy, JSS Academy of Technical Education, Noida 201301, UP, India; mtharik@jssaten.ac.in

[7] Department of Pharmaceutical Chemistry, JSS College of Pharmacy, JSS Academy of Higher Education & Research, Sri Shivarathreeshwara Nagar, Mysore 570015, KA, India; aishwaryasusil31@gmail.com (A.S.); harithadd3@gmail.com (H.H.)

* Correspondence: 1ramanbiotech@gmail.com, bathmic@jssuni.edu.in (R.R.); brprashanthkumar@jssuni.edu.in (B.R.P.K.)

## Supplementary files

# Annexure – I

Biochemical test identification results of JSSCPM1 strain.

**Biochemical test identification results of JSSCPM1 strain**

| **Test** | **JSSCPM1 strain** | ***S. marcescens*** |
|---|---|---|
| Indole production test | + | + |
| Nitrate reduction test | + | + |
| Catalase | + | + |
| Methyl red test | - | - |
| Glucose fermentation gas production test | + | + |
| Voges-Proskauert test | + | + |
| Urease | + | + |
| Oxidase | - | - |
| Acid production test (L-arabinose, D-xylose and cotton sugar fermentation) | - | - |

**Note: Positive (+), (-) negative**

# <u>**Annexure – II**</u>

Phylogenetic tree of *S. marcescens* JSSCPM1 strain and its twelve closest neighbours.

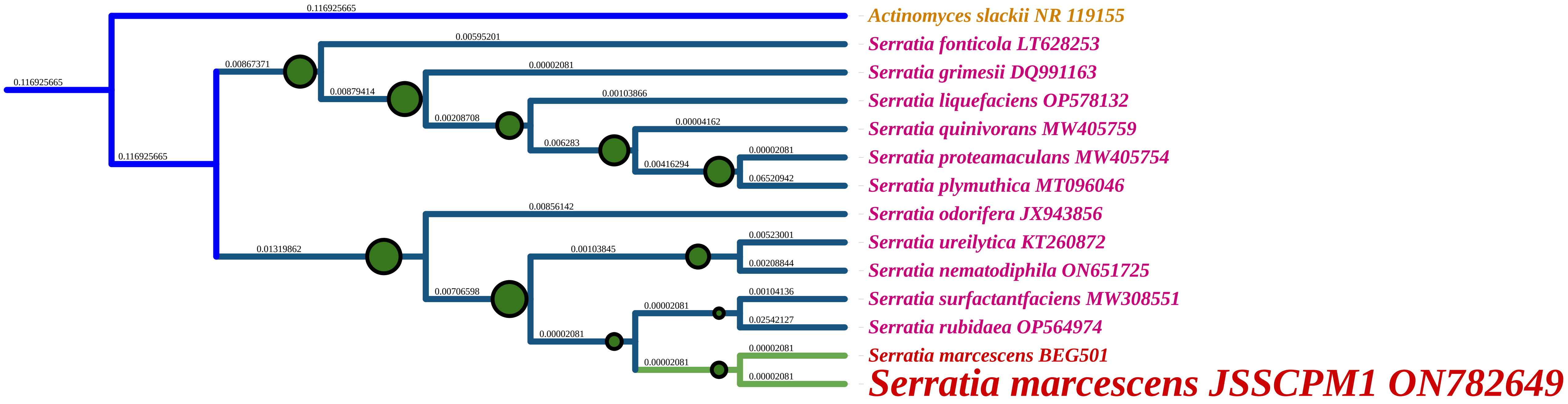
0.116925665
0.116925665
0.116925665
0.00867371
0.00595201
0.00879414
0.00002081
0.00208708
0.00103866
0.006283
0.00004162
0.00416294
0.00002081
0.06520942
0.01319862
0.00856142
0.00706598
0.00103845
0.00523001
0.00208844
0.00002081
0.00002081
0.00104136
0.02542127
0.00002081
0.00002081
0.00002081
Actinomyces slackii NR 119155
Serratia fonticola LT628253
Serratia grimesii DQ991163
Serratia liquefaciens OP578132
Serratia quinivorans MW405759
Serratia proteamaculans MW405754
Serratia plymuthica MT096046
Serratia odorifera JX943856
Serratia ureilytica KT260872
Serratia nematodiphila ON651725
Serratia surfactantfaciens MW308551
Serratia rubidaea OP564974
Serratia marcescens BEG501
Serratia marcescens JSSCPM1 ON782649

# Annexure – III

UV absorption spectrum of PG compound

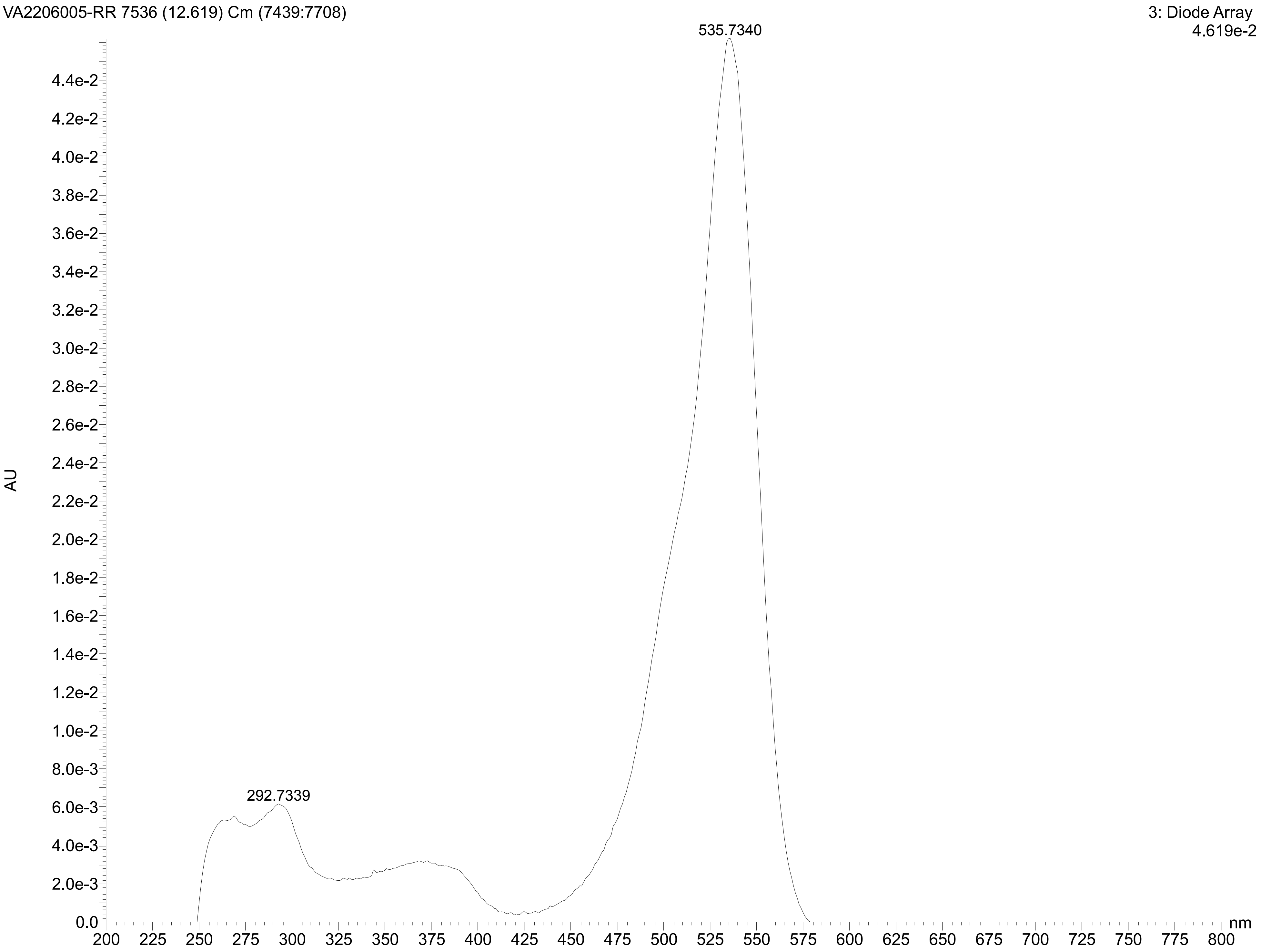

VA2206005-RR 7536 (12.619) Cm (7439:7708)
3: Diode Array
4.619e-2
535.7340
292.7339
AU
4.4e-2
4.2e-2
4.0e-2
3.8e-2
3.6e-2
3.4e-2
3.2e-2
3.0e-2
2.8e-2
2.6e-2
2.4e-2
2.2e-2
2.0e-2
1.8e-2
1.6e-2
1.4e-2
1.2e-2
1.0e-2
8.0e-3
6.0e-3
4.0e-3
2.0e-3
0.0
nm
200 225 250 275 300 325 350 375 400 425 450 475 500 525 550 575 600 625 650 675 700 725 750 775 800

# Annexure – IV

## HPLC report of PG compound

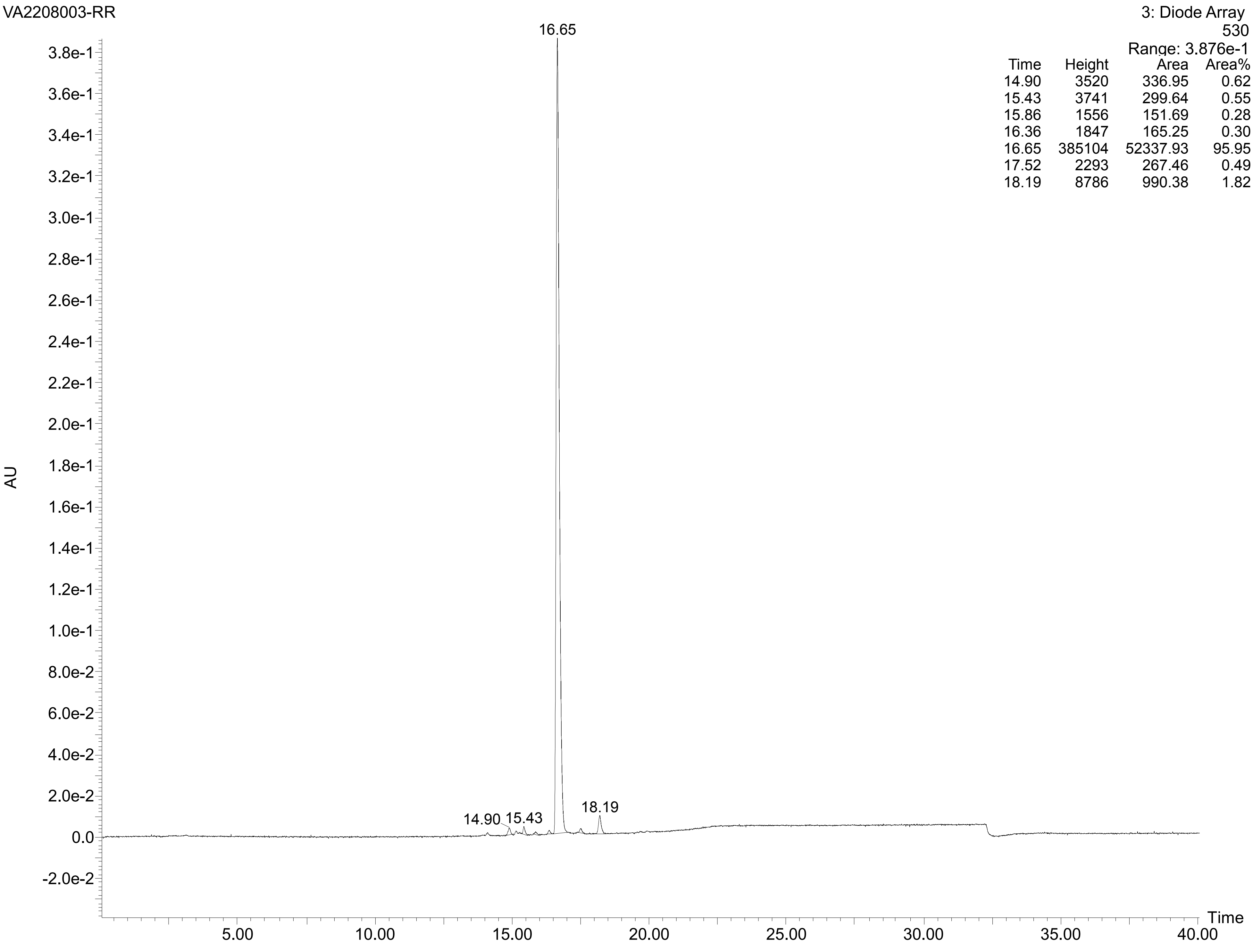


| Time | Height | Area | Area% |
|---|---|---|---|
| 14.90 | 3520 | 336.95 | 0.62 |
| 15.43 | 3741 | 299.64 | 0.55 |
| 15.86 | 1556 | 151.69 | 0.28 |
| 16.36 | 1847 | 165.25 | 0.30 |
| 16.65 | 385104 | 52337.93 | 95.95 |
| 17.52 | 2293 | 267.46 | 0.49 |
| 18.19 | 8786 | 990.38 | 1.82 |

# Annexure – V

## LC-MS analysis of PG Compound

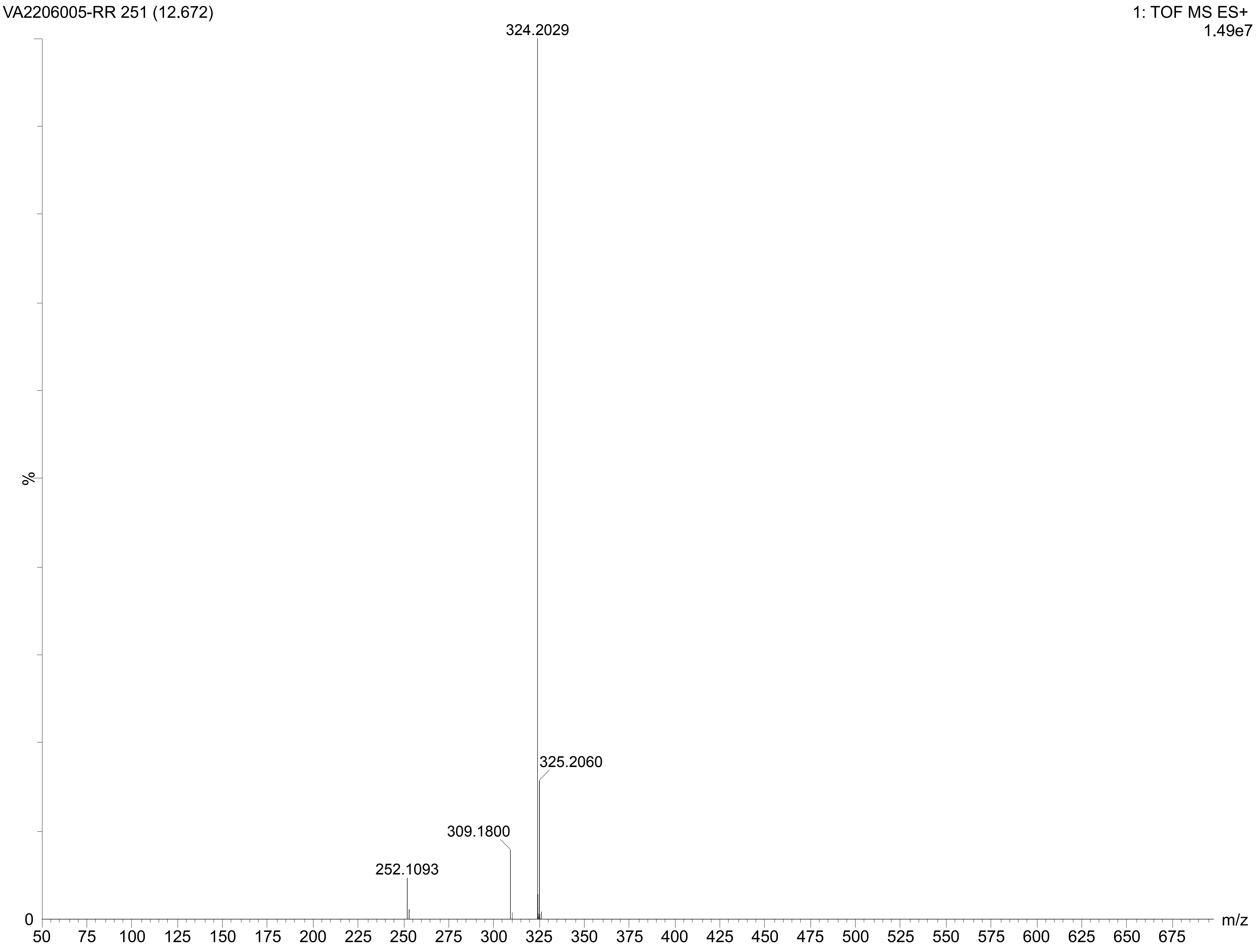
VA2206005-RR 251 (12.672)
1: TOF MS ES+
1.49e7
324.2029
325.2060
309.1800
252.1093
%
0
m/z
50
75
100
125
150
175
200
225
250
275
300
325
350
375
400
425
450
475
500
525
550
575
600
625
650
675

# Annexure –VI

## $^{1}H$ and $^{13}C$ NMR report of PG compound

**Sample RR NRM Report:**

**Bruker 400MHz NMR**

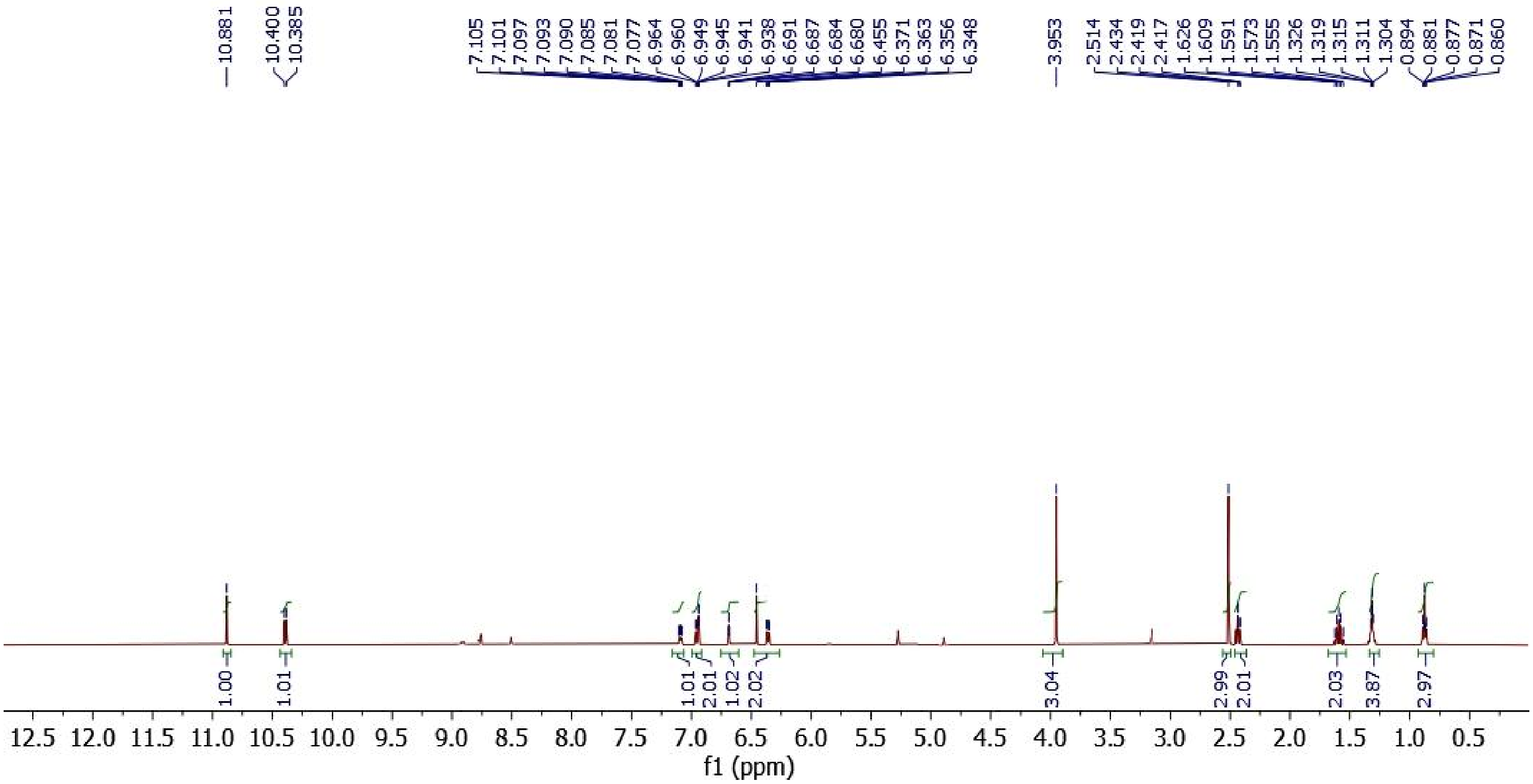

**1H NMR Spectrum of Sample RR**

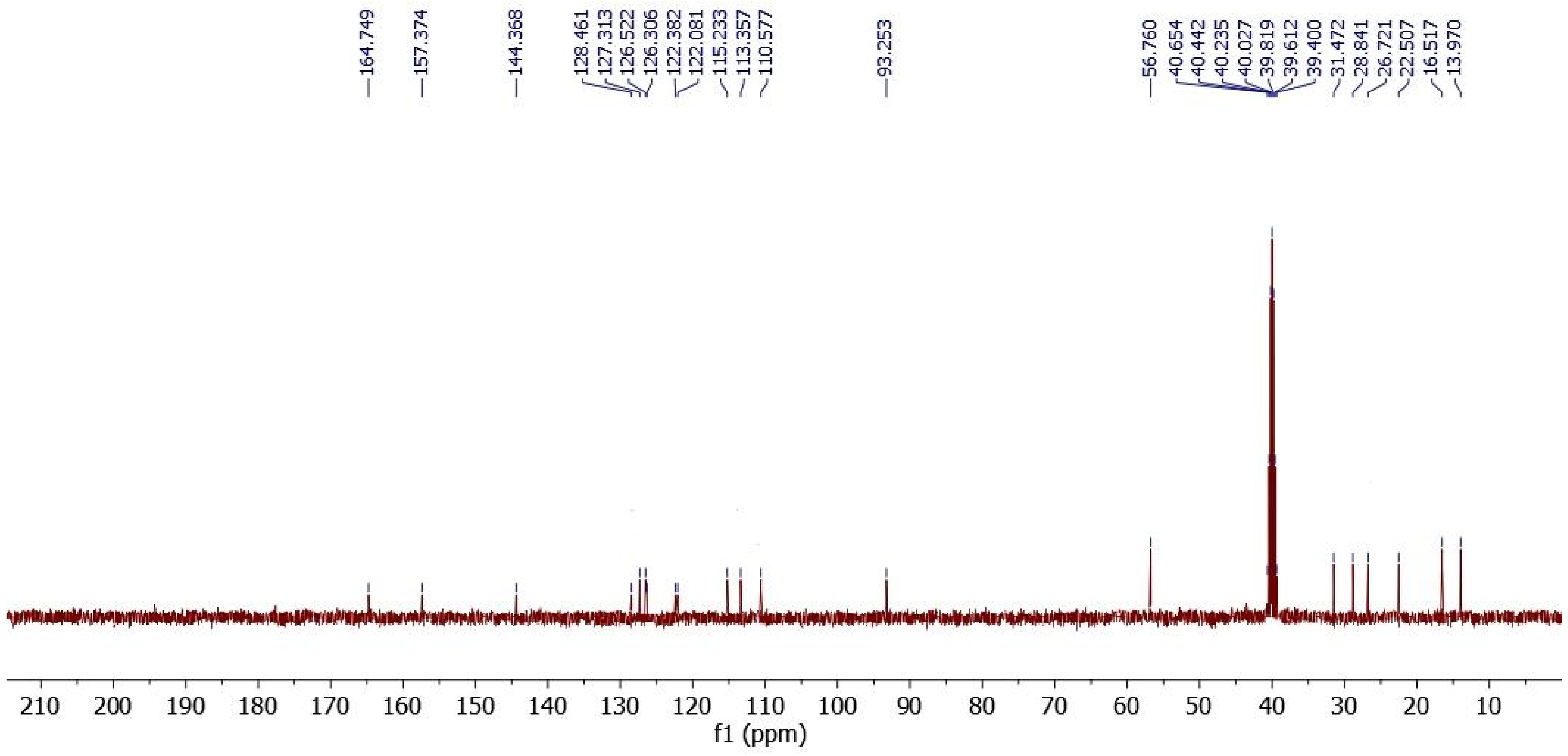


**13C NMR Spectrum of Sample RR**

# Annexure – VII

**Total 122 chromosomal genomes of *S. marcescens*, *Serratia sp.*, and *Serratia nematodiphila* used in this study, along with their name, GenBank accession number and unique database links.**

| Sl.NO | Species name | GenBank Accession number | Genome database link |
|---|---|---|---|
| 1 | *Serratia marcescens* strain M158-1-1 chromosome, complete genome | CP060440.1 | https://www.ncbi.nlm.nih.gov/nuccore/CP060440.1 |
| 2 | *Serratia marcescens* strain MGH246 chromosome | CP060487.1 | https://www.ncbi.nlm.nih.gov/nuccore/CP060487.1 |
| 3 | *Serratia marcescens* strain MV-u1-SK1-O chromosome, complete genome | CP085860.1 | https://www.ncbi.nlm.nih.gov/nuccore/CP085860.1 |
| 4 | *Serratia marcescens* strain N10A28 chromosome, complete genome | CP033623.1 | https://www.ncbi.nlm.nih.gov/nuccore/CP033623.1 |
| 5 | *Serratia marcescens* strain N4-5 chromosome, complete genome | CP031316.1 | https://www.ncbi.nlm.nih.gov/nuccore/CP031316.1 |
| 6 | *Serratia marcescens* strain RH10 chromosome, complete genome | CP092461.1 | https://www.ncbi.nlm.nih.gov/nuccore/CP092461.1 |
| 7 | *Serratia marcescens* strain RSC-14, complete genome | CP012639.1 | https://www.ncbi.nlm.nih.gov/nuccore/CP012639.1 |
| 8 | *Serratia marcescens* strain S2I7 genome | CP021984.1 | https://www.ncbi.nlm.nih.gov/nuccore/CP021984.1 |
| 9 | *Serratia marcescens* strain SARVS06 chromosome, complete genome | CP110102.1 | https://www.ncbi.nlm.nih.gov/nuccore/CP110102.1 |
| 10 | *Serratia marcescens* strain SASK1000 chromosome, complete genome | CP100753.1 | https://www.ncbi.nlm.nih.gov/nuccore/CP100753.1 |
| 11 | *Serratia marcescens* strain 12/2010 chromosome, complete genome | CP053925.1 | https://www.ncbi.nlm.nih.gov/nuccore/CP053925.1 |
| 12 | *Serratia marcescens* strain 1274 chromosome, partial genome | CP019927.2 | https://www.ncbi.nlm.nih.gov/nuccore/CP019927.2 |
| 13 | *Serratia marcescens* strain 1602 chromosome, complete genome | CP047391.1 | https://www.ncbi.nlm.nih.gov/nuccore/CP047391.1 |
| 14 | *Serratia marcescens* strain 1912768R chromosome, complete genome | CP040350.1 | https://www.ncbi.nlm.nih.gov/nuccore/CP040350.1 |
| 15 | *Serratia marcescens* strain 2838 chromosome, complete genome | CP047685.1 | https://www.ncbi.nlm.nih.gov/nuccore/CP047685.1 |
| 16 | *Serratia marcescens* strain 3024 chromosome, complete genome | CP047682.1 | https://www.ncbi.nlm.nih.gov/nuccore/CP047682.1 |
| 17 | *Serratia marcescens* strain 332 chromosome, complete genome | CP021164.1 | https://www.ncbi.nlm.nih.gov/nuccore/CP021164.1 |
| 18 | *Serratia marcescens* strain 4201 chromosome, complete genome | CP047679.1 | https://www.ncbi.nlm.nih.gov/nuccore/CP047679.1 |
| 19 | *Serratia marcescens* strain 95 chromosome, complete genome | CP020503.1 | https://www.ncbi.nlm.nih.gov/nuccore/CP020503.1 |
| 20 | *Serratia marcescens* strain UMH3 chromosome, complete genome | CP018925.1 | https://www.ncbi.nlm.nih.gov/nuccore/CP018925.1 |
| 21 | *Serratia marcescens* strain UMH5 chromosome, complete genome | CP018917.1 | https://www.ncbi.nlm.nih.gov/nuccore/CP018917.1 |
| 22 | *Serratia marcescens* strain UMH6 chromosome, complete genome | CP018926.1 | https://www.ncbi.nlm.nih.gov/nuccore/CP018926.1 |
| 23 | *Serratia marcescens* strain UMH7 chromosome, complete genome | CP018919.1 | https://www.ncbi.nlm.nih.gov/nuccore/CP018919.1 |
| 24 | *Serratia marcescens* strain UMH8 chromosome, complete genome | CP018927.1 | https://www.ncbi.nlm.nih.gov/nuccore/CP018927.1 |
| 25 | *Serratia marcescens* strain UMH9 chromosome, complete genome | CP018923.1 | https://www.ncbi.nlm.nih.gov/nuccore/CP018923.1 |
| 26 | *Serratia marcescens* strain SJC1048 genome assembly, chromosome: 1 | OX291586.1 | https://www.ncbi.nlm.nih.gov/nuccore/OX291586.1 |
| 27 | *Serratia marcescens* strain SJC1050 genome assembly, chromosome: 1 | OX291796.1 | https://www.ncbi.nlm.nih.gov/nuccore/OX291796.1 |
| 28 | *Serratia marcescens* strain SJC1051 genome assembly, chromosome: 1 | OX291746.1 | https://www.ncbi.nlm.nih.gov/nuccore/OX291746.1 |
| 29 | *Serratia marcescens* strain SJC1052 genome assembly, chromosome: 1 | OX291705.1 | https://www.ncbi.nlm.nih.gov/nuccore/OX291705.1 |
| 30 | *Serratia marcescens* strain SJC1054 genome assembly, chromosome: 1 | OX291528.1 | https://www.ncbi.nlm.nih.gov/nuccore/OX291705.1 |
| 31 | *Serratia marcescens subsp. marcescens* ATCC 13880 substr. Sm_S97_jyu2015 chromosome, complete genome | CP071202.1 | https://www.ncbi.nlm.nih.gov/nuccore/CP071202.1 |

| | | | |
|---|---|---|---|
| 32 | *Serratia marcescens subsp. marcescens* ATCC 13880 substr. Sm_S9_jyu2015 chromosome, complete genome | CP071238.1 | https://www.ncbi.nlm.nih.gov/nuccore/CP071238.1/ |
| 33 | *Serratia marcescens subsp. marcescens* ATCC 13880 substr. Sm_SA_jyu2015 chromosome, complete genome | CP071214.1 | https://www.ncbi.nlm.nih.gov/nuccore/CP071214.1/ |
| 34 | *Serratia marcescens subsp. marcescens* Db11, complete genome | HG326223.1 | https://www.ncbi.nlm.nih.gov/nuccore/HG326223.1/ |
| 35 | *Serratia marcescens* WW4, complete genome | CP003959.1 | https://www.ncbi.nlm.nih.gov/nuccore/CP003959.1/ |
| 36 | *Serratia nematodiphila* strain DH-S01 chromosome, complete genome | CP038662.1 | https://www.ncbi.nlm.nih.gov/nuccore/CP038662.1 |
| 37 | *Serratia sp.* FDAARGOS_506 chromosome, complete genome | CP033831.1 | https://www.ncbi.nlm.nih.gov/nuccore/CP033831.1 |
| 38 | *Serratia sp.* FS14, complete genome | CP005927.1 | https://www.ncbi.nlm.nih.gov/nuccore/CP005927.1 |
| 39 | *Serratia sp.* HRI chromosome, complete genome | CP083690.1 | https://www.ncbi.nlm.nih.gov/nuccore/CP083690.1 |
| 40 | *Serratia sp.* JKS000199 genome assembly, chromosome: I | LT907843.1 | https://www.ncbi.nlm.nih.gov/nuccore/LT907843.1 |
| 41 | *Serratia sp.* LS-1 chromosome, complete genome | CP033504.1 | https://www.ncbi.nlm.nih.gov/nuccore/CP033504.1 |
| 42 | *Serratia sp.* SSNIH1 chromosome, complete genome | CP026383.1 | https://www.ncbi.nlm.nih.gov/nuccore/CP026383.1 |
| 43 | *Serratia marcescens* strain UMH1 chromosome, complete genome | CP018915.1 | https://www.ncbi.nlm.nih.gov/nuccore/CP018915.1 |
| 44 | *Serratia marcescens* strain UMH10 chromosome, complete genome | CP018928.1 | https://www.ncbi.nlm.nih.gov/nuccore/CP018928.1 |
| 45 | *Serratia marcescens* strain UMH11 chromosome, complete genome | CP018929.1 | https://www.ncbi.nlm.nih.gov/nuccore/CP018929.1 |
| 46 | *Serratia marcescens* strain UMH12 chromosome, complete genome | CP018930.1 | https://www.ncbi.nlm.nih.gov/nuccore/CP018930.1 |
| 47 | *Serratia marcescens* strain UMH2 chromosome, complete genome | CP018924.1 | https://www.ncbi.nlm.nih.gov/nuccore/CP018924.1 |
| 48 | *Serratia marcescens* strain FDAARGOS_65 chromosome, complete genome | CP026050.1 | https://www.ncbi.nlm.nih.gov/nuccore/CP026050.1 |
| 49 | *Serratia marcescens* strain FDAARGOS_659 chromosome | CP050960.1 | https://www.ncbi.nlm.nih.gov/nuccore/CP050960.1 |
| 50 | *Serratia marcescens* strain FY chromosome, complete genome | CP053378.1 | https://www.ncbi.nlm.nih.gov/nuccore/CP053378.1 |
| 51 | *Serratia marcescens* strain FZSF02 chromosome, complete genome | CP053286.1 | https://www.ncbi.nlm.nih.gov/nuccore/CP053286.1 |
| 52 | *Serratia marcescens* strain ICU-2 chromosome | CP059038.1 | https://www.ncbi.nlm.nih.gov/nuccore/CP059038.1 |
| 53 | *Serratia marcescens* strain ICU-3 chromosome | CP059037.1 | https://www.ncbi.nlm.nih.gov/nuccore/CP059037.1 |
| 54 | *Serratia marcescens* strain ICU-4 chromosome | CP059036.1 | https://www.ncbi.nlm.nih.gov/nuccore/CP059036.1 |
| 55 | *Serratia marcescens* strain JW-CZ2 chromosome, complete genome | CP055161.1 | https://www.ncbi.nlm.nih.gov/nuccore/CP055161.1 |
| 56 | *Serratia marcescens* strain KS10 chromosome | CP027798.1 | https://www.ncbi.nlm.nih.gov/nuccore/CP027798.1 |
| 57 | *Serratia marcescens* strain LVF3 chromosome, complete genome | CP063229.1 | https://www.ncbi.nlm.nih.gov/nuccore/CP063229.1 |
| 58 | *Serratia marcescens* strain WVU-002 chromosome, complete genome | CP041123.1 | https://www.ncbi.nlm.nih.gov/nuccore/CP041123.1 |
| 59 | *Serratia marcescens* strain WVU-004 chromosome, complete genome | CP041125.1 | https://www.ncbi.nlm.nih.gov/nuccore/CP041125.1 |
| 60 | *Serratia marcescens* strain WVU-007 chromosome, complete genome | CP041130.1 | https://www.ncbi.nlm.nih.gov/nuccore/CP041130.1 |
| 61 | *Serratia marcescens* strain WVU-008 chromosome, complete genome | CP041131.1 | https://www.ncbi.nlm.nih.gov/nuccore/CP041131.1 |
| 62 | *Serratia marcescens* strain WVU-009 chromosome, complete genome | CP041132.1 | https://www.ncbi.nlm.nih.gov/nuccore/CP041132.1 |
| 63 | *Serratia marcescens* strain WVU-010 chromosome, complete genome | CP041134.1 | https://www.ncbi.nlm.nih.gov/nuccore/CP041134.1 |

| | | | |
|---|---|---|---|
| 64 | *Serratia marcescens* strain YHYF1 chromosome, complete genome | CP092184.1 | https://www.ncbi.nlm.nih.gov/nuccore/CP092184.1 |
| 65 | *Serratia marcescens subsp. marcescens* ATCC 13880 chromosome, complete genome | CP072199.1 | https://www.ncbi.nlm.nih.gov/nuccore/CP072199.1 |
| 66 | *Serratia marcescens subsp. marcescens* ATCC 13880 chromosome, complete genome | CP041233.1 | https://www.ncbi.nlm.nih.gov/nuccore/CP041233.1 |
| 67 | *Serratia marcescens subsp. marcescens* ATCC 13880 substr. Sm_S13_jyu2015 chromosome, complete genome | CP071236.1 | https://www.ncbi.nlm.nih.gov/nuccore/CP071236.1 |
| 68 | *Serratia marcescens subsp. marcescens* ATCC 13880 substr. Sm_S22_jyu2015 chromosome, complete genome | CP071226.1 | https://www.ncbi.nlm.nih.gov/nuccore/CP071226.1 |
| 69 | *Serratia marcescens subsp. marcescens* ATCC 13880 substr. Sm_S24_jyu2015 chromosome, complete genome | CP071216.1 | https://www.ncbi.nlm.nih.gov/nuccore/CP071216.1 |
| 70 | *Serratia marcescens subsp. marcescens* ATCC 13880 substr. Sm_S28_jyu2015 chromosome, complete genome | CP071222.1 | https://www.ncbi.nlm.nih.gov/nuccore/CP071222.1 |
| 71 | *Serratia marcescens subsp. marcescens* ATCC 13880 substr. Sm_S33_jyu2015 chromosome, complete genome | CP071224.1 | https://www.ncbi.nlm.nih.gov/nuccore/CP071224.1 |
| 72 | *Serratia marcescens subsp. marcescens* ATCC 13880 substr. Sm_S37_jyu2015 chromosome, complete genome | CP071244.1 | https://www.ncbi.nlm.nih.gov/nuccore/CP071244.1 |
| 73 | *Serratia marcescens subsp. marcescens* ATCC 13880 substr. Sm_S57_jyu2015 chromosome, complete genome | CP071220.1 | https://www.ncbi.nlm.nih.gov/nuccore/CP071220.1 |
| 74 | *Serratia marcescens subsp. marcescens* ATCC 13880 substr. Sm_S60_jyu2015 chromosome, complete genome | CP071192.1 | https://www.ncbi.nlm.nih.gov/nuccore/CP071192.1 |
| 75 | *Serratia marcescens subsp. marcescens* ATCC 13880 substr. Sm_S64_jyu2015 chromosome, complete genome | CP071240.1 | https://www.ncbi.nlm.nih.gov/nuccore/CP071240.1 |
| 76 | *Serratia marcescens subsp. marcescens* ATCC 13880 substr. Sm_S65_jyu2015 chromosome, complete genome | CP071218.1 | https://www.ncbi.nlm.nih.gov/nuccore/CP071218.1 |
| 77 | *Serratia marcescens subsp. marcescens* ATCC 13880 substr. Sm_S67_jyu2015 chromosome, complete genome | CP071232.1 | https://www.ncbi.nlm.nih.gov/nuccore/CP071232.1 |
| 78 | *Serratia marcescens* strain BP2 chromosome, complete genome | CP050013.1 | https://www.ncbi.nlm.nih.gov/nuccore/CP050013.1 |
| 79 | *Serratia marcescens* strain BWH-23 chromosome, complete genome | CP020501.1 | https://www.ncbi.nlm.nih.gov/nuccore/CP020501.1 |
| 80 | *Serratia marcescens* strain BWH-35 chromosome, complete genome | CP020507.1 | https://www.ncbi.nlm.nih.gov/nuccore/CP020507.1 |
| 81 | *Serratia marcescens* strain Byron chromosome, complete genome | CP054277.1 | https://www.ncbi.nlm.nih.gov/nuccore/CP054277.1 |
| 82 | *Serratia marcescens* strain C110 chromosome, complete genome | CP047691.1 | https://www.ncbi.nlm.nih.gov/nuccore/CP047691.1 |
| 83 | *Serratia marcescens* strain CM2012_028 chromosome, complete genome | CP091122.1 | https://www.ncbi.nlm.nih.gov/nuccore/CP091122.1 |
| 84 | *Serratia marcescens* strain E28 chromosome, complete genome | CP042512.1 | https://www.ncbi.nlm.nih.gov/nuccore/CP042512.1 |
| 85 | *Serratia marcescens* strain EL1 chromosome | CP027796.1 | https://www.ncbi.nlm.nih.gov/nuccore/CP027796.1 |
| 86 | *Serratia marcescens* strain ESE2014 chromosome, complete genome | CP058353.1 | https://www.ncbi.nlm.nih.gov/nuccore/CP058353.1 |
| 87 | *Serratia marcescens* strain SJC1058 genome assembly, chromosome: 1 | OX291724.1 | https://www.ncbi.nlm.nih.gov/nuccore/OX291724.1 |
| 88 | *Serratia marcescens* strain SJC1061 genome assembly, chromosome: 1 | OX291610.1 | https://www.ncbi.nlm.nih.gov/nuccore/OX291610.1 |

| | | | |
|---|---|---|---|
| 89 | *Serratia marcescens* strain SJC1062 genome assembly, chromosome: 1 | OX291474.1 | https://www.ncbi.nlm.nih.gov/nuccore/OX291474.1 |
| 90 | *Serratia marcescens* strain SJC1070 genome assembly, chromosome: 1 | OX291687.1 | https://www.ncbi.nlm.nih.gov/nuccore/OX291687.1 |
| 91 | *Serratia marcescens* strain SJC1039 genome assembly, chromosome: 1 | OX291654.1 | https://www.ncbi.nlm.nih.gov/nuccore/OX291654.1 |
| 92 | *Serratia marcescens* strain SJC1043 genome assembly, chromosome: 1 | OX291536.1 | https://www.ncbi.nlm.nih.gov/nuccore/OX291536.1 |
| 93 | *Serratia marcescens* strain SJC1044 genome assembly, chromosome: 1 | OX291771.1 | https://www.ncbi.nlm.nih.gov/nuccore/OX291771.1 |
| 94 | *Serratia marcescens* strain SJC1045 genome assembly, chromosome: 1 | OX291470.1 | https://www.ncbi.nlm.nih.gov/nuccore/OX291470.1 |
| 95 | *Serratia marcescens* strain SJC1046 genome assembly, chromosome: 1 | OX291532.1 | https://www.ncbi.nlm.nih.gov/nuccore/OX291532.1 |
| 96 | *Serratia marcescens subsp. marcescens* ATCC 13880 substr. Sm_S68_jyu2015 chromosome, complete genome | CP071198.1 | https://www.ncbi.nlm.nih.gov/nuccore/CP071198.1 |
| 97 | *Serratia marcescens subsp. marcescens* ATCC 13880 substr. Sm_S6_jyu2015 chromosome, complete genome | CP071204.1 | https://www.ncbi.nlm.nih.gov/nuccore/CP071204.1 |
| 98 | *Serratia marcescens subsp. marcescens* ATCC 13880 substr. Sm_S71_jyu2015 chromosome, complete genome | CP071206.1 | https://www.ncbi.nlm.nih.gov/nuccore/CP071206.1 |
| 99 | *Serratia marcescens subsp. marcescens* ATCC 13880 substr. Sm_S78_jyu2015 chromosome, complete genome | CP071228.1 | https://www.ncbi.nlm.nih.gov/nuccore/CP071228.1 |
| 100 | *Serratia marcescens subsp. marcescens* ATCC 13880 substr. Sm_S79_jyu2015 chromosome, complete genome | CP071190.1 | https://www.ncbi.nlm.nih.gov/nuccore/CP071190.1 |
| 101 | *Serratia marcescens subsp. marcescens* ATCC 13880 substr. Sm_S81_jyu2015 chromosome, complete genome | CP071208.1 | https://www.ncbi.nlm.nih.gov/nuccore/CP071208.1 |
| 102 | *Serratia marcescens subsp. marcescens* ATCC 13880 substr. Sm_S89_jyu2015 chromosome, complete genome | CP071210.1 | https://www.ncbi.nlm.nih.gov/nuccore/CP071210.1 |
| 103 | *Serratia marcescens subsp. marcescens* ATCC 13880 substr. Sm_S94_jyu2015 chromosome, complete genome | CP071188.1 | https://www.ncbi.nlm.nih.gov/nuccore/CP071188.1 |
| 104 | *Serratia marcescens subsp. marcescens* ATCC 13880 substr. Sm_S95_jyu2015 chromosome, complete genome | CP071234.1 | https://www.ncbi.nlm.nih.gov/nuccore/CP071234.1 |
| 105 | *Serratia marcescens subsp. marcescens* ATCC 13880 substr. Sm_S96_jyu2015 chromosome, complete genome | CP071200.1 | https://www.ncbi.nlm.nih.gov/nuccore/CP071200.1 |
| 106 | *Serratia marcescens* strain SCH909 chromosome, complete genome | CP063238.1 | https://www.ncbi.nlm.nih.gov/nuccore/CP063238.1 |
| 107 | *Serratia marcescens* strain SCQ1 chromosome, complete genome | CP063354.1 | https://www.ncbi.nlm.nih.gov/nuccore/CP063354.1 |
| 108 | *Serratia marcescens* strain SGAir0764 chromosome, complete genome | CP027300.1 | https://www.ncbi.nlm.nih.gov/nuccore/CP027300.1 |
| 109 | *Serratia marcescens* strain SMBC50 chromosome, complete genome | CP109829.1 | https://www.ncbi.nlm.nih.gov/nuccore/CP109829.1 |
| 110 | *Serratia marcescens* strain SMNSF-1 chromosome, complete genome | CP090244.1 | https://www.ncbi.nlm.nih.gov/nuccore/CP090244.1 |
| 111 | *Serratia marcescens* strain SmUNAM836 chromosome, complete sequence | CP012685.1 | https://www.ncbi.nlm.nih.gov/nuccore/CP012685.1 |
| 112 | *Serratia marcescens* strain Sys06 chromosome, complete genome | CP090908.1 | https://www.ncbi.nlm.nih.gov/nuccore/CP090908.1 |
| 113 | *Serratia marcescens* strain U36365 chromosome, complete genome | CP016032.1 | https://www.ncbi.nlm.nih.gov/nuccore/CP016032.1 |
| 114 | *Serratia marcescens* 2020-O-9 DNA, complete genome | AP024847.1 | https://www.ncbi.nlm.nih.gov/nuccore/AP024847.1 |

| | | | |
|---|---|---|---|
| 115 | *Serratia marcescens* AS-1 DNA, complete genome | AP019009.1 | https://www.ncbi.nlm.nih.gov/nuccore/AP019009.1 |
| 116 | *Serratia marcescens* ATCC 274 DNA, complete genome | AP021873.1 | https://www.ncbi.nlm.nih.gov/nuccore/AP021873.1 |
| 117 | *Serratia marcescens* isolate GN26 chromosome | CP026650.1 | https://www.ncbi.nlm.nih.gov/nuccore/CP026650.1 |
| 118 | *Serratia marcescens* isolate PWN146_assembly genome assembly, chromosome: Chromosome | LT575490.1 | https://www.ncbi.nlm.nih.gov/nuccore/LT575490.1 |
| 119 | *Serratia marcescens* SM39 DNA, complete genome | AP013063.1 | https://www.ncbi.nlm.nih.gov/nuccore/AP013063.1 |
| 120 | *Serratia marcescens* SMB2099 complete genome | HG738868.1 | https://www.ncbi.nlm.nih.gov/nuccore/HG738868.1 |
| 121 | *Serratia marcescens* strain 11/2010 chromosome, complete genome | CP053927.1 | https://www.ncbi.nlm.nih.gov/nuccore/CP053927.1 |
| 122 | *Serratia marcescens* strain 1140- chromosome, complete genome | CP047688.1 | https://www.ncbi.nlm.nih.gov/nuccore/CP047688.1 |

# Annexure – VIII

**Comprehensive comparative analysis of PG producing BGCs in 122 *Serratia* species belonging to three different categories**

| Type | Location | | Bioactive compound | Most Known cluster | similarity |
|---|---|---|---|---|---|
| | From | To | | | |
| *Serratia marcescens* strain M158-1-1 chromosome, complete genome | | | | | |
| NRPS | 1,768,922 | 1,815,529 | pyrronazol B | NRP + Polyketide | 9% |
| RRE-containing | 1,849,816 | 1,870,094 | lankacidin C | NRP + Polyketide | 13% |
| NRPS | 1,946,631 | 1,994,234 | microcin H47 | RiPP:Microcin | 20% |
| thiopeptide | 2,571,915 | 2,598,359 | O-antigen | Saccharide | 14% |
| betalactone | 3,600,139 | 3,625,807 | - | - | - |
| hserlactone | 3,802,810 | 3,823,454 | - | - | - |
| NRPS | 3,959,473 | 4,043,669 | vulnibactin | NRP | 18% |
| NRPS | 4,812,080 | 4,856,018 | R1128 | Polyketide | 14% |
| *Serratia marcescens* strain MGH246 chromosome | | | | | |
| thiopeptide | 255,818 | 282,261 | O-antigen | Saccharide | 14% |
| RRE-containing | 992,988 | 1,013,266 | lankacidin C | NRP + Polyketide | 13% |
| NRPS | 1,033,752 | 1,081,677 | pyrronazol B | NRP + Polyketide | 9% |
| NRPS | 1,719,642 | 1,805,531 | - | - | - |
| siderophore | 2,998,995 | 3,010,854 | - | - | - |
| NRPS | 3,198,021 | 3,271,448 | xenotetrapeptide | NRP | 100% |
| NRPS | 4,045,919 | 4,105,114 | vulnibactin | NRP | 18% |
| NRPS-like,hserlactone | 4,238,536 | 4,289,518 | - | - | - |
| betalactone | 4,483,768 | 4,509,437 | - | - | - |
| NRPS,T1PKS | 4,912,950 | 4,969,288 | olimycin A / olimycin B | Polyketide | 5% |
| *Serratia marcescens* strain MV-u1-SK1-O chromosome, complete genome | | | | | |
| NRPS | 301,925 | 360,320 | vulnibactin | NRP | 18% |
| NRPS-like,hserlactone | 484,411 | 536,204 | - | - | - |
| betalactone | 744,225 | 769,893 | - | - | - |
| thiopeptide | 1,723,598 | 1,750,041 | O-antigen | Saccharide | 14% |
| NRPS | 2,318,574 | 2,365,587 | microcin H47 | RiPP:Microcin | 20% |
| RRE-containing | 2,452,198 | 2,472,476 | lankacidin C | NRP + Polyketide | 13% |
| NRPS | 2,488,797 | 2,534,905 | pyrronazol B | NRP + Polyketide | 9% |

| | | | | | |
|---|---|---|---|---|---|
| T1PKS,NRPS | 3,147,495 | 3,209,493 | althiomycin | NRP | 100% |
| NRPS | 4,612,536 | 4,670,316 | rhizomide A / rhizomide B / rhizomide C | NRP | 100% |
| *Serratia marcescens* strain N10A28 chromosome, complete genome | | | | | |
| betalactone | 61,318 | 86,987 | - | - | - |
| NRPS,T1PKS | 540,336 | 595,959 | olimycin A / olimycin B | Polyketide | 5% |
| thiopeptide | 1,159,811 | 1,186,254 | O-antigen | Saccharide | 14% |
| RRE-containing | 1,897,121 | 1,917,399 | lankacidin C | NRP + Polyketide | 13% |
| NRPS | 1,939,073 | 1,986,863 | pyrronazol B | NRP + Polyketide | 9% |
| NRPS | 2,613,416 | 2,699,302 | - | - | - |
| siderophore | 3,879,359 | 3,891,218 | - | - | - |
| NRPS | 4,078,386 | 4,135,283 | xenotetrapeptide | NRP | 100% |
| NRPS | 4,906,759 | 4,966,587 | turnerbactin | NRP | 30% |
| NRPS-like | 5,094,203 | 5,137,166 | - | - | - |
| *Serratia marcescens* strain N4-5 chromosome, complete genome | | | | | |
| NRPS-like,hserlactone | 464,549 | 515,758 | - | - | - |
| betalactone | 718,310 | 743,980 | - | - | - |
| **prodigiosin** | **1,096,070** | **1,131,090** | **prodigiosin** | **Polyketide** | **100%** |
| thiopeptide | 1,709,166 | 1,735,610 | O-antigen | Saccharide | 14% |
| NRPS | 2,327,560 | 2,372,840 | microcin H47 | RiPP:Microcin | 20% |
| redox-cofactor | 2,461,838 | 2,484,000 | lankacidin C | NRP + Polyketide | 13% |
| NRPS | 2,511,923 | 2,559,392 | pyrronazol B | NRP + Polyketide | 9% |
| NRPS | 4,524,652 | 4,568,587 | xantholipin | Polyketide | 4% |
| *Serratia marcescens* strain RH10 chromosome, complete genome | | | | | |
| NRPS | 315,410 | 357,890 | enterobactin | NRP | 12% |
| NRPS-like | 480,852 | 523,292 | - | - | - |
| betalactone | 739,899 | 765,566 | - | - | - |
| thiopeptide | 1,706,630 | 1,733,074 | O-antigen | Saccharide | 14% |
| NRPS | 2,368,316 | 2,415,325 | microcin H47 | RiPP:Microcin | 20% |
| redox-cofactor | 2,493,929 | 2,516,091 | lankacidin C | NRP + Polyketide | 13% |

| | | | | | |
|---|---|---|---|---|---|
| NRPS | 2,545,383 | 2,593,893 | pyrronazol B | NRP + Polyketide | 9% |
| *Serratia marcescens* strain RSC-14, complete genome | | | | | |
| thiopeptide | 321,461 | 347,209 | - | - | - |
| NRPS | 937,262 | 982,472 | - | - | - |
| betalactone | 1,352,119 | 1,377,787 | - | - | - |
| NRPS | 1,744,602 | 1,803,283 | vulnibactin | NRP | 12% |
| hserlactone | 2,035,550 | 2,056,224 | - | - | - |
| NRPS | 2,578,081 | 2,634,840 | rhizomide A / rhizomide B / rhizomide C | NRP | 100% |
| thiopeptide,LAP | 3,389,285 | 3,418,062 | - | - | - |
| NRPS | 3,926,126 | 4,011,902 | - | - | - |
| NRPS | 4,632,414 | 4,680,924 | pyrronazol B | NRP + Polyketide | 9% |
| RRE-containing | 4,712,243 | 4,732,521 | lankacidin C | NRP + Polyketide | 13% |
| *Serratia marcescens* strain S2I7 genome | | | | | |
| NRPS | 329,927 | 384,346 | vulnibactin | NRP | 18% |
| betalactone | 807,967 | 833,637 | - | - | - |
| redox-cofactor | 1,030,071 | 1,051,482 | lankacidin C | NRP + Polyketide | 13% |
| **prodigiosin** | **1,204,774** | **1,239,794** | **prodigiosin** | **Polyketide** | **100%** |
| thiopeptide | 1,842,979 | 1,869,424 | O-antigen | Saccharide | 14% |
| redox-cofactor | 2,595,048 | 2,617,210 | lankacidin C | NRP + Polyketide | 13% |
| NRPS | 2,635,571 | 2,682,118 | pyrronazol B | NRP + Polyketide | 9% |
| NRPS | 3,359,430 | 3,403,192 | - | - | - |
| NRPS | 4,730,647 | 4,774,585 | xantholipin | Polyketide | 4% |
| *Serratia marcescens* strain SARVS06 chromosome, complete genome | | | | | |
| NRPS | 74,291 | 115,597 | bicornutin A1 / bicornutin A2 | NRP | 100% |
| NRPS | 1,457,516 | 1,501,271 | - | - | - |
| NRPS | 2,147,453 | 2,193,972 | pyrronazol B | NRP + Polyketide | 9% |
| RRE-containing | 2,216,040 | 2,233,241 | lankacidin C | NRP + Polyketide | 13% |
| thiopeptide | 2,987,192 | 3,013,636 | O-antigen | Saccharide | 14% |
| **prodigiosin** | **3,605,836** | **3,640,856** | **prodigiosin** | **Polyketide** | **100%** |

| | | | | | |
|---|---|---|---|---|---|
| redox-cofactor | 3,793,423 | 3,815,585 | lankacidin C | NRP + Polyketide | 13% |
| betalactone | 3,986,125 | 4,011,795 | - | - | - |
| NRPS | 4,417,714 | 4,460,809 | vulnibactin | NRP | 12% |
| *Serratia marcescens* strain SASK1000 chromosome, complete genome | | | | | |
| NRPS | 74,291 | 115,598 | bicornutin A1 / bicornutin A2 | NRP | 100% |
| NRPS | 1,457,519 | 1,501,273 | - | - | - |
| NRPS | 2,147,454 | 2,193,971 | pyrronazol B | NRP + Polyketide | 9% |
| redox-cofactor | 2,213,833 | 2,234,891 | lankacidin C | NRP + Polyketide | 13% |
| thiopeptide | 2,987,190 | 3,013,634 | O-antigen | Saccharide | 14% |
| **prodigiosin** | **3,605,830** | **3,640,850** | **prodigiosin** | **Polyketide** | **100%** |
| redox-cofactor | 3,793,416 | 3,815,578 | lankacidin C | NRP + Polyketide | 13% |
| betalactone | 3,986,116 | 4,011,786 | - | - | - |
| NRPS | 4,400,963 | 4,460,804 | vulnibactin | NRP | 18% |
| *Serratia marcescens* strain 12/2010 chromosome, complete genome | | | | | |
| NRPS | 287,085 | 345,646 | vulnibactin | NRP | 18% |
| NRPS-like,hserlactone | 459,515 | 510,629 | - | - | - |
| betalactone | 717,332 | 743,001 | - | - | - |
| **NRPS** | **1,110,632** | **1,155,304** | **prodigiosin** | **Polyketide** | **12%** |
| thiopeptide | 1,747,804 | 1,774,245 | O-antigen | Saccharide | 14% |
| NRPS | 2,119,200 | 2,172,276 | - | - | - |
| RRE-containing | 2,585,999 | 2,606,277 | lankacidin C | NRP + Polyketide | 13% |
| NRPS | 2,621,159 | 2,667,417 | - | - | - |
| NRPS | 3,485,251 | 3,534,269 | - | - | - |
| NRPS | 5,063,102 | 5,119,952 | xenotetrapeptide | NRP | 100% |
| NRPS,T1PKS | 5,355,540 | 5,410,162 | - | - | - |
| *Serratia marcescens* strain 1274 chromosome, partial genome | | | | | |
| thiopeptide | 736,176 | 762,618 | O-antigen | Saccharide | 14% |
| betalactone | 1,828,191 | 1,853,858 | - | - | - |
| hserlactone | 2,042,741 | 2,063,433 | - | - | - |

| | | | | | |
|---|---|---|---|---|---|
| NRPS | 2,218,300 | 2,260,684 | turnerbactin | NRP | 30% |
| NRPS,NRPS-like | 3,998,394 | 4,052,053 | lysobactin | NRP | 2% |
| siderophore | 4,239,977 | 4,251,836 | - | - | - |
| NRPS | 4,310,601 | 4,396,437 | - | - | - |
| NRPS | 5,026,004 | 5,074,514 | pyrronazol B | NRP + Polyketide | 9% |
| RRE-containing | 5,095,122 | 5,115,400 | lankacidin C | NRP + Polyketide | 13% |
| *Serratia marcescens* strain 1602 chromosome, complete genome | | | | | |
| NRPS | 270,830 | 329,366 | vulnibactin | NRP | 18% |
| NRPS-like,hserlactone | 444,964 | 497,080 | - | - | - |
| betalactone | 700,290 | 725,960 | - | - | - |
| **NRPS** | **1,089,090** | **1,133,764** | **prodigiosin** | **Polyketide** | **12%** |
| thiopeptide | 1,733,034 | 1,759,475 | O-antigen | Saccharide | 14% |
| NRPS | 2,363,456 | 2,409,846 | colicin V | RiPP | 1% |
| RRE-containing | 2,497,536 | 2,517,814 | lankacidin C | NRP + Polyketide | 13% |
| NRPS | 2,539,518 | 2,586,327 | - | - | - |
| NRPS | 3,222,740 | 3,271,248 | - | - | - |
| NRPS | 4,539,011 | 4,594,551 | rhizomide A / rhizomide B / rhizomide C | NRP | 100% |
| NRPS,T1PKS | 4,829,806 | 4,884,428 | - | - | - |
| *Serratia marcescens* strain 1912768R chromosome, complete genome | | | | | |
| NRPS | 1,219,713 | 1,263,481 | - | - | - |
| NRPS | 1,955,399 | 2,001,998 | turnerbactin | NRP | 15% |
| redox-cofactor | 2,019,297 | 2,040,267 | lankacidin C | NRP + Polyketide | 13% |
| thiopeptide | 2,707,712 | 2,734,156 | O-antigen | Saccharide | 14% |
| **prodigiosin** | **3,328,113** | **3,363,133** | **prodigiosin** | **Polyketide** | **100%** |
| redox-cofactor | 3,515,714 | 3,537,876 | lankacidin C | NRP + Polyketide | 13% |
| betalactone | 3,708,560 | 3,734,230 | - | - | - |
| NRPS | 4,140,914 | 4,199,727 | vulnibactin | NRP | 18% |
| NRPS | 4,997,021 | 5,040,959 | xantholipin | Polyketide | 4% |
| *Serratia marcescens* strain 2838 chromosome, complete genome | | | | | |

| | | | | | |
|---|---|---|---|---|---|
| NRPS | 292,397 | 350,100 | vulnibactin | NRP | 18% |
| NRPS-like | 474,518 | 516,297 | - | - | - |
| betalactone | 772,671 | 798,340 | - | - | - |
| **NRPS** | **1,191,319** | **1,235,993** | **prodigiosin** | **Polyketide** | **12%** |
| thiopeptide | 1,833,431 | 1,859,872 | O-antigen | Saccharide | 14% |
| NRPS | 2,547,860 | 2,594,559 | colicin V | RiPP | 1% |
| RRE-containing | 2,679,230 | 2,699,508 | lankacidin C | NRP + Polyketide | 13% |
| NRPS | 2,719,697 | 2,764,840 | pyrronazol B | NRP + Polyketide | 9% |
| NRPS | 3,407,003 | 3,456,553 | lipopolysaccharide | Saccharide:Lipopolysaccharide | 5% |
| NRPS | 3,519,745 | 3,576,602 | gobichelin A / gobichelin B | NRP | 11% |
| RiPP-like | 3,918,291 | 3,930,837 | - | - | - |
| NRPS | 4,830,442 | 4,888,294 | rhizomide A / rhizomide B / rhizomide C | NRP | 100% |
| *Serratia marcescens* strain 3024 chromosome, complete genome | | | | | |
| NRPS | 789,965 | 846,809 | rhizomide A / rhizomide B / rhizomide C | NRP | 100% |
| RiPP-like | 1,746,396 | 1,758,942 | - | - | - |
| NRPS | 2,100,631 | 2,157,488 | gobichelin A / gobichelin B | NRP | 11% |
| NRPS | 2,220,680 | 2,270,230 | lipopolysaccharide | Saccharide:Lipopolysaccharide | 5% |
| NRPS | 2,913,492 | 2,958,635 | pyrronazol B | NRP + Polyketide | 9% |
| RRE-containing | 2,978,824 | 2,999,102 | lankacidin C | NRP + Polyketide | 13% |
| NRPS | 3,083,773 | 3,130,472 | colicin V | RiPP | 1% |
| thiopeptide | 3,818,626 | 3,845,067 | O-antigen | Saccharide | 14% |
| **NRPS** | **4,442,505** | **4,487,179** | **prodigiosin** | **Polyketide** | **12%** |
| betalactone | 4,880,157 | 4,905,826 | - | - | - |
| NRPS-like | 5,162,200 | 5,203,979 | - | - | - |
| NRPS | 5,326,799 | 5,386,622 | vulnibactin | NRP | 18% |
| *Serratia marcescens* strain 332 chromosome, complete genome | | | | | |
| NRPS | 281,130 | 339,279 | vulnibactin | NRP | 18% |
| betalactone | 698,703 | 724,372 | - | - | - |
| thiopeptide | 1,685,588 | 1,712,031 | O-antigen | Saccharide | 14% |

| | | | | | |
|---|---|---|---|---|---|
| RRE-containing | 2,356,430 | 2,376,708 | lankacidin C | NRP + Polyketide | 13% |
| NRPS | 2,410,990 | 2,457,623 | pyrronazol B | NRP + Polyketide | 9% |
| RiPP-like | 3,647,550 | 3,660,078 | - | - | - |
| NRPS | 4,521,966 | 4,565,904 | R1128 | Polyketide | 14% |
| *Serratia marcescens* strain 4201 chromosome, complete genome | | | | | |
| hserlactone | 23,319 | 43,993 | - | - | - |
| NRPS | 277,466 | 336,713 | vulnibactin | NRP | 12% |
| betalactone | 711,467 | 737,135 | - | - | - |
| **NRPS-like,NRPS** | **1,126,560** | **1,171,227** | **prodigiosin** | **Polyketide** | **12%** |
| thiopeptide | 1,819,149 | 1,844,869 | O-antigen | Saccharide | 14% |
| RRE-containing | 2,539,939 | 2,560,217 | lankacidin C | NRP + Polyketide | 13% |
| T1PKS,NRPS | 2,572,441 | 2,642,698 | pyrronazol B | NRP + Polyketide | 9% |
| NRPS | 3,284,769 | 3,370,545 | - | - | - |
| thiopeptide,LAP,NRPS | 3,870,841 | 3,918,187 | microcin E492 | RiPP:Microcin | 12% |
| NRPS | 4,774,975 | 4,832,821 | rhizomide A / rhizomide B / rhizomide C | NRP | 100% |
| *Serratia marcescens* strain 95 chromosome, complete genome | | | | | |
| NRPS | 267,640 | 326,049 | turnerbactin | NRP | 30% |
| NRPS-like | 440,629 | 482,641 | - | - | - |
| betalactone | 686,207 | 711,877 | - | - | - |
| thiopeptide | 1,823,248 | 1,849,690 | O-antigen | Saccharide | 14% |
| siderophore | 1,999,540 | 2,011,399 | - | - | - |
| redox-cofactor | 2,570,922 | 2,593,084 | lankacidin C | NRP + Polyketide | 13% |
| T1PKS,NRPS | 3,264,723 | 3,327,660 | althiomycin | NRP | 100% |
| NRPS | 3,455,571 | 3,500,770 | - | - | - |
| NRPS | 4,730,531 | 4,788,383 | rhizomide A / rhizomide B / rhizomide C | NRP | 100% |
| *Serratia marcescens* strain UMH3 chromosome, complete genome | | | | | |
| betalactone | 60,940 | 86,609 | - | - | - |
| **NRPS** | **428,720** | **473,393** | **prodigiosin** | **Polyketide** | **12%** |
| thiopeptide | 1,077,451 | 1,103,892 | O-antigen | Saccharide | 14% |

| | | | | | |
|---|---|---|---|---|---|
| NRPS | 1,735,352 | 1,782,064 | colicin V | RiPP | 1% |
| RRE-containing | 1,865,618 | 1,885,896 | lankacidin C | NRP + Polyketide | 13% |
| NRPS | 1,935,243 | 1,981,754 | turnerbactin | NRP | 15% |
| NRPS | 2,686,444 | 2,734,951 | - | - | - |
| NRPS | 4,078,128 | 4,134,978 | rhizomide A / rhizomide B / rhizomide C | NRP | 100% |
| NRPS,T1PKS | 4,357,877 | 4,412,036 | - | - | - |
| NRPS | 4,918,550 | 4,978,379 | vulnibactin | NRP | 18% |
| NRPS-like,hserlactone | 5,092,894 | 5,144,974 | | | |
| *Serratia marcescens* strain UMH5 chromosome, complete genome | | | | | |
| betalactone | 60,796 | 86,465 | - | - | - |
| thiopeptide | 1,153,113 | 1,179,556 | O-antigen | Saccharide | 14% |
| RRE-containing | 1,883,518 | 1,903,796 | lankacidin C | NRP + Polyketide | 13% |
| NRPS | 1,924,541 | 1,972,355 | pyrronazol B | NRP + Polyketide | 9% |
| NRPS | 2,598,877 | 2,684,754 | - | - | - |
| siderophore | 3,841,687 | 3,853,546 | - | - | - |
| NRPS | 4,913,885 | 4,973,712 | vulnibactin | NRP | 18% |
| hserlactone | 5,213,524 | 5,234,168 | - | - | - |
| *Serratia marcescens* strain UMH6 chromosome, complete genome | | | | | |
| betalactone | 61,010 | 86,679 | | | |
| NRPS,T1PKS | 448,346 | 548,890 | olimycin A / olimycin B | Polyketide | 5% |
| thiopeptide | 1,098,383 | 1,124,825 | O-antigen | Saccharide | 14% |
| RRE-containing | 1,868,800 | 1,889,078 | lankacidin C | NRP + Polyketide | 13% |
| NRPS | 1,889,898 | 1,937,878 | turnerbactin | NRP | 15% |
| NRPS | 2,590,530 | 2,667,119 | ravidomycin | Polyketide | 5% |
| NRPS | 3,995,027 | 4,049,933 | rhizomide A / rhizomide B / rhizomide C | NRP | 100% |
| NRPS | 4,234,941 | 4,277,402 | pseudomonine | NRP | 20% |
| hserlactone | 4,502,685 | 4,523,359 | - | - | - |
| NRPS | 4,840,443 | 4,884,387 | enterobactin | NRP | 12% |
| *Serratia marcescens* strain UMH7 chromosome, complete genome | | | | | |

| | | | | | |
|---|---|---|---|---|---|
| betalactone | 61,010 | 86,678 | | | |
| **NRPS** | **457,943** | **503,165** | **prodigiosin** | **Polyketide** | **12%** |
| thiopeptide | 1,126,065 | 1,151,789 | O-antigen | Saccharide | 14% |
| RRE-containing | 1,846,706 | 1,866,984 | lankacidin C | NRP + Polyketide | 13% |
| T1PKS,NRPS | 1,877,903 | 1,947,780 | pyrronazol B | NRP + Polyketide | 9% |
| NRPS | 2,560,316 | 2,636,614 | - | - | - |
| thiopeptide,LAP,NRPS | 3,146,832 | 3,192,346 | microcin E492 | RiPP:Microcin | 12% |
| NRPS | 3,987,938 | 4,044,684 | rhizomide A / rhizomide B / rhizomide C | NRP | 100% |
| hserlactone | 4,566,104 | 4,586,778 | - | - | - |
| NRPS | 4,819,283 | 4,879,273 | vulnibactin | NRP | 12% |
| *Serratia marcescens* strain UMH8 chromosome, complete genome | | | | | |
| betalactone | 61,029 | 86,698 | - | - | - |
| **prodigiosin** | **443,511** | **478,531** | **prodigiosin** | **Polyketide** | **100%** |
| thiopeptide | 1,057,373 | 1,083,817 | O-antigen | Saccharide | 14% |
| NRPS | 1,665,800 | 1,712,675 | microcin H47 | RiPP:Microcin | 20% |
| redox-cofactor | 1,789,938 | 1,812,100 | lankacidin C | NRP + Polyketide | 13% |
| NRPS | 1,837,358 | 1,883,756 | pyrronazol B | NRP + Polyketide | 9% |
| NRPS | 3,910,884 | 3,954,107 | xantholipin | Polyketide | 4% |
| NRPS | 4,762,634 | 4,822,454 | vulnibactin | NRP | 12% |
| NRPS-like | 4,944,552 | 4,987,530 | - | - | - |
| *Serratia marcescens* strain UMH9 chromosome, complete genome | | | | | |
| betalactone | 60,933 | 86,602 | | | |
| **NRPS** | **439,738** | **484,387** | **prodigiosin** | **Polyketide** | **12%** |
| thiopeptide | 1,065,875 | 1,092,316 | O-antigen | Saccharide | 14% |
| NRPS | 1,634,363 | 1,681,070 | colicin V | RiPP | 1% |
| RRE-containing | 1,769,262 | 1,789,540 | lankacidin C | NRP + Polyketide | 13% |
| NRPS | 1,807,920 | 1,854,010 | pyrronazol B | NRP + Polyketide | 9% |
| NRPS | 2,478,653 | 2,527,576 | - | - | - |
| NRPS | 3,788,524 | 3,845,379 | xenotetrapeptide | NRP | 100% |

| | | | | | |
|---|---|---|---|---|---|
| NRPS,T1PKS | 4,068,151 | 4,121,045 | - | - | - |
| NRPS | 4,642,493 | 4,702,316 | vulnibactin | NRP | 18% |
| *Serratia marcescens* strain SJC1048 genome assembly, chromosome: 1 | | | | | |
| NRPS | 291,794 | 351,526 | vulnibactin | NRP | 12% |
| NRPS-like | 475,241 | 517,428 | - | - | - |
| RRE-containing | 524,603 | 539,264 | - | - | - |
| betalactone | 745,983 | 771,652 | - | - | - |
| thiopeptide | 1,842,573 | 1,869,016 | O-antigen | Saccharide | 14% |
| siderophore | 2,054,663 | 2,066,522 | - | - | - |
| redox-cofactor | 2,592,864 | 2,615,026 | lankacidin C | NRP + Polyketide | 13% |
| T1PKS,NRPS | 3,248,078 | 3,311,145 | althiomycin | NRP | 100% |
| NRPS | 3,376,840 | 3,421,933 | - | - | - |
| NRPS | 3,487,193 | 3,533,250 | yersiniabactin | NRP + Polyketide | 4% |
| NRPS | 4,820,838 | 4,875,468 | rhizomide A / rhizomide B / rhizomide C | NRP | 100% |
| *Serratia marcescens* strain SJC1050 genome assembly, chromosome: 1 | | | | | |
| T1PKS,NRPS | 1 | 44,123 | althiomycin | NRP | 100% |
| NRPS | 109,818 | 154,911 | - | - | - |
| NRPS | 220,171 | 266,228 | yersiniabactin | NRP + Polyketide | 4% |
| NRPS | 1,553,851 | 1,608,189 | rhizomide A / rhizomide B / rhizomide C | NRP | 100% |
| NRPS | 2,457,726 | 2,517,717 | vulnibactin | NRP | 12% |
| NRPS-like | 2,641,027 | 2,684,008 | - | - | - |
| RRE-containing | 2,690,757 | 2,705,418 | - | - | - |
| betalactone | 2,912,137 | 2,937,806 | - | - | - |
| thiopeptide | 4,008,405 | 4,034,848 | O-antigen | Saccharide | 14% |
| siderophore | 4,220,495 | 4,232,354 | - | - | - |
| redox-cofactor | 4,758,697 | 4,780,859 | lankacidin C | NRP + Polyketide | 13% |
| *Serratia marcescens* strain SJC1051 genome assembly, chromosome: 1 | | | | | |
| NRPS | 291,793 | 351,524 | vulnibactin | NRP | 12% |
| NRPS-like | 475,239 | 517,426 | - | - | - |

| | | | | | |
|---|---|---|---|---|---|
| RRE-containing | 524,601 | 539,262 | - | - | - |
| betalactone | 745,981 | 771,650 | - | - | - |
| thiopeptide | 995,870 | 1,022,313 | O-antigen | Saccharide | 14% |
| siderophore | 2,054,373 | 2,066,232 | - | - | - |
| redox-cofactor | 2,638,910 | 2,661,072 | lankacidin C | NRP + Polyketide | 13% |
| T1PKS,NRPS | 3,294,136 | 3,357,203 | althiomycin | NRP | 100% |
| NRPS | 3,422,898 | 3,467,991 | - | - | - |
| NRPS | 3,533,251 | 3,579,308 | yersiniabactin | NRP + Polyketide | 4% |
| NRPS | 4,866,940 | 4,921,570 | rhizomide A / rhizomide B / rhizomide C | NRP | 100% |
| *Serratia marcescens* strain SJC1052 genome assembly, chromosome: 1 | | | | | |
| thiopeptide | 249,683 | 276,126 | O-antigen | Saccharide | 14% |
| siderophore | 461,773 | 473,632 | - | - | - |
| redox-cofactor | 999,974 | 1,022,136 | lankacidin C | NRP + Polyketide | 13% |
| T1PKS,NRPS | 1,655,187 | 1,718,254 | althiomycin | NRP | 100% |
| NRPS | 1,783,949 | 1,829,042 | - | - | - |
| NRPS | 1,894,302 | 1,940,359 | yersiniabactin | NRP + Polyketide | 4% |
| NRPS | 3,229,636 | 3,282,371 | cichopeptin | NRP | 46% |
| NRPS | 4,131,908 | 4,191,899 | vulnibactin | NRP | 12% |
| NRPS-like | 4,315,209 | 4,358,190 | - | - | - |
| RRE-containing | 4,362,837 | 4,383,103 | - | - | - |
| betalactone | 4,586,311 | 4,611,980 | - | - | - |
| *Serratia marcescens* strain SJC1054 genome assembly, chromosome: 1 | | | | | |
| NRPS | 291,794 | 351,526 | vulnibactin | NRP | 12% |
| NRPS-like | 475,241 | 517,428 | - | - | - |
| RRE-containing | 524,603 | 539,264 | - | - | - |
| betalactone | 745,983 | 771,652 | - | - | - |
| thiopeptide | 1,842,414 | 1,868,857 | O-antigen | Saccharide | 14% |
| siderophore | 2,054,504 | 2,066,363 | - | - | - |
| redox-cofactor | 2,592,705 | 2,614,867 | lankacidin C | NRP + Polyketide | 13% |

| | | | | | |
|---|---|---|---|---|---|
| T1PKS,NRPS | 3,247,919 | 3,310,986 | althiomycin | NRP | 100% |
| NRPS | 3,376,681 | 3,421,774 | - | - | - |
| NRPS | 3,487,034 | 3,533,091 | yersiniabactin | NRP + Polyketide | 4% |
| NRPS | 4,822,071 | 4,875,261 | orfamide A / orfamide C | NRP:Cyclic depsipeptide | 17% |
| *Serratia marcescens subsp. marcescens* ATCC 13880 substr. Sm_S97_jyu2015 chromosome, complete genome | | | | | |
| NRPS | 299,729 | 358,346 | vulnibactin | NRP | 12% |
| NRPS-like | 480,799 | 520,920 | - | - | - |
| betalactone | 758,651 | 784,322 | - | - | - |
| **prodigiosin** | **1,139,869** | **1,174,889** | **prodigiosin** | **Polyketide** | **100%** |
| thiopeptide | 1,767,991 | 1,794,435 | O-antigen | Saccharide | 14% |
| NRPS | 2,368,848 | 2,415,859 | microcin H47 | RiPP:Microcin | 20% |
| redox-cofactor | 2,490,765 | 2,512,927 | lankacidin C | NRP + Polyketide | 13% |
| NRPS | 2,542,776 | 2,589,195 | pyrronazol B | NRP + Polyketide | 9% |
| NRPS | 4,577,071 | 4,621,009 | xantholipin | Polyketide | 4% |
| *Serratia marcescens subsp. marcescens* ATCC 13880 substr. Sm_S9_jyu2015 chromosome, complete genome | | | | | |
| NRPS | 299,729 | 358,346 | vulnibactin | NRP | 12% |
| NRPS-like | 480,799 | 520,920 | - | - | - |
| betalactone | 758,651 | 784,322 | - | - | - |
| **prodigiosin** | **1,139,869** | **1,174,889** | **prodigiosin** | **Polyketide** | **100%** |
| thiopeptide | 1,767,948 | 1,794,392 | O-antigen | Saccharide | 14% |
| NRPS | 2,368,805 | 2,415,816 | microcin H47 | RiPP:Microcin | 20% |
| redox-cofactor | 2,490,722 | 2,512,884 | lankacidin C | NRP + Polyketide | 13% |
| NRPS | 2,542,733 | 2,589,152 | pyrronazol B | NRP + Polyketide | 9% |
| NRPS | 4,577,027 | 4,620,965 | xantholipin | Polyketide | 4% |
| *Serratia marcescens subsp. marcescens* ATCC 13880 substr. Sm_SA_jyu2015 chromosome, complete genome | | | | | |
| NRPS | 299,729 | 358,346 | vulnibactin | NRP | 12% |
| NRPS-like | 480,799 | 520,920 | - | - | - |
| betalactone | 758,651 | 784,322 | - | - | - |
| **prodigiosin** | **1,139,869** | **1,174,889** | **prodigiosin** | **Polyketide** | **100%** |
| thiopeptide | 1,767,949 | 1,794,393 | O-antigen | Saccharide | 14% |

| | | | | | |
|---|---|---|---|---|---|
| NRPS | 2,368,806 | 2,415,817 | microcin H47 | RiPP:Microcin | 20% |
| redox-cofactor | 2,490,723 | 2,512,885 | lankacidin C | NRP + Polyketide | 13% |
| NRPS | 2,542,734 | 2,589,153 | pyrronazol B | NRP + Polyketide | 9% |
| NRPS | 4,577,029 | 4,620,967 | xantholipin | Polyketide | 4% |
| *Serratia marcescens subsp. marcescens* Db11, complete genome | | | | | |
| betalactone | 60,868 | 86,536 | - | - | - |
| thiopeptide | 1,024,305 | 1,050,756 | O-antigen | Saccharide | 14% |
| NRPS | 1,619,286 | 1,666,298 | microcin H47 | RiPP:Microcin | 20% |
| RRE-containing | 1,754,535 | 1,774,813 | lankacidin C | NRP + Polyketide | 13% |
| NRPS | 1,796,558 | 1,843,060 | pyrronazol B | NRP + Polyketide | 9% |
| T1PKS,NRPS | 2,395,828 | 2,457,825 | althiomycin | NRP | 100% |
| arylpolyene | 3,839,497 | 3,883,093 | aryl polyenes | Other | 100% |
| NRPS | 3,883,633 | 3,940,276 | rhizomide A / rhizomide B / rhizomide C | NRP | 100% |
| RiPP-like | 4,500,368 | 4,512,896 | - | - | - |
| NRPS | 4,715,800 | 4,775,756 | vulnibactin | NRP | 18% |
| NRPS-like | 4,933,385 | 4,976,369 | - | - | - |
| *Serratia marcescens* WW4, complete genome | | | | | |
| NRPS | 329,936 | 388,977 | vulnibactin | NRP | 18% |
| betalactone | 807,967 | 833,637 | - | - | - |
| redox-cofactor | 1,030,071 | 1,051,482 | lankacidin C | NRP + Polyketide | 13% |
| **prodigiosin** | **1,204,774** | **1,239,794** | **prodigiosin** | **Polyketide** | **100%** |
| thiopeptide | 1,842,979 | 1,869,424 | O-antigen | Saccharide | 14% |
| redox-cofactor | 2,595,048 | 2,617,210 | lankacidin C | NRP + Polyketide | 13% |
| NRPS | 2,635,571 | 2,682,136 | pyrronazol B | NRP + Polyketide | 9% |
| NRPS | 3,359,430 | 3,403,192 | - | - | - |
| NRPS | 4,730,647 | 4,774,585 | xantholipin | Polyketide | 4% |
| *Serratia nematodiphila* strain DH-S01 chromosome, complete genome | | | | | |
| NRPS | 270,826 | 329,600 | vulnibactin | NRP | 18% |
| betalactone | 737,420 | 763,089 | - | - | - |

| | | | | | |
|---|---|---|---|---|---|
| redox-cofactor | 966,089 | 987,140 | lankacidin C | NRP + Polyketide | 13% |
| **NRPS,prodigiosin** | **1,138,450** | **1,199,322** | **prodigiosin** | **Polyketide** | **100%** |
| thiopeptide | 1,785,395 | 1,811,839 | O-antigen | Saccharide | 14% |
| NRPS | 2,418,527 | 2,464,724 | microcin H47 | RiPP:Microcin | 13% |
| redox-cofactor | 2,543,865 | 2,566,027 | lankacidin C | NRP + Polyketide | 13% |
| NRPS | 2,574,938 | 2,622,872 | - | - | - |
| NRPS | 3,240,739 | 3,317,255 | ravidomycin | Polyketide | 5% |
| NRPS | 4,706,161 | 4,750,096 | xantholipin | Polyketide | 4% |
| *Serratia sp.* FDAARGOS_506 chromosome, complete genome | | | | | |
| NRPS | 981,778 | 1,024,295 | R1128 | Polyketide | 14% |
| NRPS | 1,788,075 | 1,847,910 | turnerbactin | NRP | 30% |
| betalactone | 2,183,474 | 2,209,141 | - | - | - |
| thiopeptide | 3,154,703 | 3,181,145 | O-antigen | Saccharide | 14% |
| RRE-containing | 3,804,251 | 3,824,529 | lankacidin C | NRP + Polyketide | 13% |
| *Serratia sp.* FS14, complete genome | | | | | |
| betalactone | 118,946 | 144,616 | - | - | - |
| NRPS | 520,270 | 562,659 | enterobactin | NRP | 12% |
| NRPS | 1,367,361 | 1,410,107 | xantholipin | Polyketide | 4% |
| NRPS-like,thiopeptide,LAP | 2,282,421 | 2,329,041 | microcin E492 | RiPP:Microcin | 12% |
| NRPS | 2,838,063 | 2,914,575 | ravidomycin | Polyketide | 5% |
| NRPS | 3,584,001 | 3,632,511 | turnerbactin | NRP | 15% |
| redox-cofactor | 3,635,442 | 3,657,604 | lankacidin C | NRP + Polyketide | 13% |
| thiopeptide | 4,341,885 | 4,368,329 | O-antigen | Saccharide | 14% |
| **prodigiosin** | **4,984,802** | **5,019,822** | **prodigiosin** | **Polyketide** | **100%** |
| *Serratia sp.* HRI chromosome, complete genome | | | | | |
| RRE-containing | 51,492 | 71,770 | lankacidin C | NRP + Polyketide | 13% |
| siderophore | 615,034 | 626,893 | - | - | - |
| thiopeptide | 778,811 | 805,253 | O-antigen | Saccharide | 14% |
| betalactone | 1,898,614 | 1,924,284 | - | - | - |
| NRPS-like | 2,160,759 | 2,202,954 | - | - | - |

| | | | | | |
|---|---|---|---|---|---|
| NRPS | 2,319,451 | 2,378,422 | vulnibactin | NRP | 12% |
| NRPS | 3,173,401 | 3,230,608 | rhizomide A / rhizomide B / rhizomide C | NRP | 100% |
| NRPS | 4,641,719 | 4,684,660 | - | - | - |
| NRPS,T1PKS | 4,772,658 | 4,835,570 | althiomycin | NRP | 100% |
| *Serratia sp.* JKS000199 genome assembly, chromosome: I | | | | | |
| **NRPS** | **407,871** | **452,538** | **prodigiosin** | **Polyketide** | **12%** |
| thiopeptide | 1,057,678 | 1,083,422 | O-antigen | Saccharide | 14% |
| RRE-containing | 1,722,738 | 1,743,016 | lankacidin C | NRP + Polyketide | 13% |
| T1PKS,NRPS | 1,754,014 | 1,823,956 | pyrronazol B | NRP + Polyketide | 9% |
| NRPS | 2,444,730 | 2,530,512 | | | |
| thiopeptide,LAP,NRPS | 3,031,011 | 3,078,357 | microcin E492 | RiPP:Microcin | 12% |
| NRPS | 3,848,791 | 3,905,540 | xenotetrape-ptide | NRP- | -100% |
| hserlactone | 4,434,954 | 4,455,628 | - | - | - |
| NRPS | 4,687,820 | 4,747,807 | vulnibactin | NRP | 12% |
| *Serratia sp.* LS-1 chromosome, complete genome | | | | | |
| betalactone | 60,998 | 86,666 | - | - | - |
| **NRPS** | **458,441** | **503,640** | **prodigiosin** | **Polyketide** | **12%** |
| thiopeptide | 1,131,760 | 1,157,501 | O-antigen | Saccharide | 14% |
| RRE-containing | 1,826,788 | 1,847,066 | lankacidin C | NRP + Polyketide | 13% |
| T1PKS,NRPS | 1,856,123 | 1,926,240 | pyrronazol B | NRP + Polyketide | 9% |
| NRPS | 2,545,248 | 2,621,858 | ravidomycin | Polyketide | 5% |
| thiopeptide,LAP,NRPS | 3,124,460 | 3,171,810 | microcin E492 | RiPP:Microcin | 12% |
| NRPS | 3,919,990 | 3,976,734 | rhizomide A / rhizomide B / rhizomide C | NRP | 100% |
| hserlactone | 4,513,748 | 4,534,422 | - | - | - |
| NRPS | 4,767,060 | 4,827,050 | vulnibactin | NRP | 12% |
| *Serratia sp.* SSNIH1 chromosome, complete genome | | | | | |
| NRPS | 775,160 | 834,356 | vulnibactin | NRP | 12% |
| NRPS-like | 912,465 | 992,709 | - | - | - |
| betalactone | 1,197,509 | 1,223,182 | - | - | - |

| | | | | | |
|---|---|---|---|---|---|
| thiopeptide | 2,273,286 | 2,299,728 | O-antigen | Saccharide | 14% |
| siderophore | 2,449,698 | 2,461,557 | - | - | - |
| redox-cofactor | 2,970,905 | 2,993,067 | lankacidin C | NRP + Polyketide | 13% |
| T1PKS,NRPS | 3,696,584 | 3,757,175 | althiomycin | NRP | 87% |
| NRPS | 3,808,016 | 3,853,115 | - | - | - |
| NRPS | 5,189,111 | 5,246,963 | rhizomide A / rhizomide B / rhizomide C | NRP | 100% |
| *Serratia marcescens* strain UMH1 chromosome, complete genome | | | | | |
| betalactone | 60,784 | 86,452 | - | - | - |
| thiopeptide | 1,050,165 | 1,076,609 | O-antigen | Saccharide | 14% |
| NRPS | 1,654,124 | 1,701,133 | microcin H47 | RiPP:Microcin | 20% |
| RRE-containing | 1,785,712 | 1,805,990 | lankacidin C | NRP + Polyketide | 13% |
| NRPS | 1,827,873 | 1,874,287 | pyrronazol B | NRP + Polyketide | 9% |
| T1PKS,NRPS | 2,436,069 | 2,498,066 | althiomycin | NRP | 100% |
| NRPS | 3,858,692 | 3,913,460 | rhizomide A / rhizomide B / rhizomide C | NRP | 100% |
| NRPS | 4,695,729 | 4,755,684 | vulnibactin | NRP | 18% |
| NRPS-like,hserlactone | 4,881,993 | 4,934,209 | - | - | - |
| *Serratia marcescens* strain UMH10 chromosome, complete genome | | | | | |
| betalactone | 60,785 | 86,453 | - | - | - |
| thiopeptide | 1,048,395 | 1,074,838 | O-antigen | Saccharide | 14% |
| NRPS | 1,642,516 | 1,689,526 | microcin H47 | RiPP:Microcin | 20% |
| RRE-containing | 1,776,113 | 1,796,391 | lankacidin C | NRP + Polyketide | 13% |
| NRPS | 1,818,797 | 1,865,274 | pyrronazol B | NRP + Polyketide | 9% |
| T1PKS,NRPS | 2,436,245 | 2,498,248 | althiomycin | NRP | 100% |
| NRPS | 3,948,603 | 4,003,373 | rhizomide A / rhizomide B / rhizomide C | NRP | 100% |
| NRPS | 4,812,232 | 4,872,188 | vulnibactin | NRP | 18% |
| NRPS-like,hserlactone | 4,996,478 | 5,048,547 | - | - | - |
| *Serratia marcescens* strain UMH11 chromosome, complete genome | | | | | |
| betalactone | 60,785 | 86,453 | - | - | - |

| | | | | | |
|---|---|---|---|---|---|
| thiopeptide | 1,048,396 | 1,074,839 | O-antigen | Saccharide | 14% |
| NRPS | 1,642,520 | 1,689,530 | microcin H47 | RiPP:Microcin | 20% |
| RRE-containing | 1,776,117 | 1,796,395 | lankacidin C | NRP + Polyketide | 13% |
| NRPS | 1,818,801 | 1,865,278 | pyrronazol B | NRP + Polyketide | 9% |
| T1PKS,NRPS | 2,436,248 | 2,498,251 | althiomycin | NRP | 100% |
| NRPS | 3,948,606 | 4,003,376 | rhizomide A / rhizomide B / rhizomide C | NRP | 100% |
| NRPS | 4,812,235 | 4,872,191 | vulnibactin | NRP | 18% |
| NRPS-like,hserlactone | 4,996,482 | 5,048,551 | - | - | - |
| *Serratia marcescens* strain UMH12 chromosome, complete genome | | | | | |
| betalactone | 60,784 | 86,452 | - | - | - |
| thiopeptide | 1,041,164 | 1,067,607 | O-antigen | Saccharide | 14% |
| NRPS | 1,640,561 | 1,687,563 | microcin H47 | RiPP:Microcin | 20% |
| RRE-containing | 1,777,303 | 1,797,581 | lankacidin C | NRP + Polyketide | 13% |
| NRPS | 1,819,465 | 1,865,716 | pyrronazol B | NRP + Polyketide | 9% |
| T1PKS,NRPS | 2,558,541 | 2,620,538 | althiomycin | NRP | 100% |
| NRPS | 3,989,895 | 4,044,666 | rhizomide A / rhizomide B / rhizomide C | NRP | 100% |
| NRPS | 4,828,128 | 4,888,083 | vulnibactin | NRP | 18% |
| NRPS-like,hserlactone | 5,010,822 | 5,062,889 | - | - | - |
| *Serratia marcescens* strain UMH2 chromosome, complete genome | | | | | |
| betalactone | 61,013 | 86,681 | - | - | - |
| thiopeptide | 1,098,231 | 1,123,986 | O-antigen | Saccharide | 14% |
| RRE-containing | 1,862,442 | 1,882,720 | lankacidin C | NRP + Polyketide | 13% |
| T1PKS,NRPS | 1,909,785 | 1,979,587 | pyrronazol B | NRP + Polyketide | 9% |
| NRPS | 2,618,711 | 2,704,491 | - | - | - |
| thiopeptide,LAP,NRPS | 3,220,159 | 3,267,506 | microcin E492 | RiPP:Microcin | 12% |
| NRPS | 4,082,584 | 4,139,329 | xenotetrapeptide | NRP | 100% |
| hserlactone | 4,688,976 | 4,709,650 | - | - | - |
| NRPS | 4,941,738 | 5,001,728 | vulnibactin | NRP | 12% |
| *Serratia marcescens* strain FDAARGOS_65 chromosome, complete genome | | | | | |

| | | | | | |
|---|---|---|---|---|---|
| NRPS | 357,494 | 402,638 | pyrronazol B | NRP + Polyketide | 9% |
| RRE-containing | 423,039 | 443,317 | lankacidin C | NRP + Polyketide | 13% |
| NRPS | 525,965 | 572,683 | colicin V | RiPP | 1% |
| thiopeptide | 1,190,014 | 1,216,455 | O-antigen | Saccharide | 14% |
| **NRPS** | **1,812,941** | **1,857,616** | **prodigiosin** | **Polyketide** | **12%** |
| betalactone | 2,250,588 | 2,276,257 | - | - | - |
| hserlactone | 2,489,101 | 2,509,793 | - | - | - |
| NRPS | 2,664,903 | 2,722,606 | vulnibactin | NRP | 18% |
| NRPS | 3,529,737 | 3,586,582 | rhizomide A / rhizomide B / rhizomide C | NRP | 100% |
| RiPP-like | 4,442,267 | 4,454,813 | - | - | - |
| NRPS | 4,794,323 | 4,851,180 | gobichelin A / gobichelin B | NRP | 11% |
| NRPS | 4,914,220 | 4,964,236 | lipopolysaccharide | Saccharide:Lipopolysaccharide | 5% |
| *Serratia marcescens* strain FDAARGOS_659 chromosome | | | | | |
| betalactone | 103,673 | 129,343 | - | - | - |
| redox-cofactor | 306,443 | 326,929 | lankacidin C | NRP + Polyketide | 13% |
| **prodigiosin** | **481,796** | **516,816** | **prodigiosin** | **Polyketide** | **100%** |
| thiopeptide | 1,095,484 | 1,121,928 | O-antigen | Saccharide | 14% |
| redox-cofactor | 1,784,320 | 1,806,482 | lankacidin C | NRP + Polyketide | 13% |
| NRPS | 1,820,062 | 1,867,803 | pyrronazol B | NRP + Polyketide | 9% |
| NRPS | 2,504,556 | 2,548,312 | - | - | - |
| NRPS | 3,922,400 | 3,965,575 | xantholipin | Polyketide | 4% |
| NRPS | 4,736,164 | 4,796,006 | vulnibactin | NRP | 18% |
| *Serratia marcescens* strain FY chromosome, complete genome | | | | | |
| NRPS | 279,793 | 338,100 | vulnibactin | NRP | 12% |
| betalactone | 689,261 | 714,929 | - | - | - |
| redox-cofactor | 894,323 | 915,612 | lankacidin C | NRP + Polyketide | 13% |
| **prodigiosin** | **1,068,190** | **1,103,210** | **prodigiosin** | **Polyketide** | **100%** |
| thiopeptide | 1,687,236 | 1,713,680 | O-antigen | Saccharide | 14% |
| NRPS | 2,283,030 | 2,328,314 | microcin H47 | RiPP:Microcin | 20% |

| | | | | | |
|---|---|---|---|---|---|
| redox-cofactor | 2,409,143 | 2,431,305 | lankacidin C | NRP + Polyketide | 13% |
| NRPS | 2,434,744 | 2,481,565 | turnerbactin | NRP | 15% |
| NRPS | 3,131,895 | 3,175,651 | - | - | - |
| RiPP-like | 3,619,671 | 3,632,199 | - | - | - |
| NRPS | 4,503,410 | 4,547,348 | xantholipin | Polyketide | 4% |
| *Serratia marcescens* strain FZSF02 chromosome, complete genome | | | | | |
| NRPS | 292,086 | 351,080 | vulnibactin | NRP | 18% |
| betalactone | 750,034 | 775,704 | - | - | - |
| **prodigiosin** | **1,149,547** | **1,184,567** | **prodigiosin** | **Polyketide** | **100%** |
| thiopeptide | 1,831,194 | 1,857,638 | O-antigen | Saccharide | 14% |
| redox-cofactor | 2,572,385 | 2,594,547 | lankacidin C | NRP + Polyketide | 13% |
| NRPS | 2,620,445 | 2,667,611 | turnerbactin | NRP | 15% |
| NRPS | 3,333,029 | 3,376,799 | - | - | - |
| NRPS | 4,778,265 | 4,822,203 | xantholipin | Polyketide | 4% |
| *Serratia marcescens* strain ICU-2 chromosome | | | | | |
| hserlactone | 68,823 | 89,497 | - | - | - |
| NRPS-like | 506,362 | 548,668 | - | - | - |
| betalactone | 769,294 | 794,963 | | - | - |
| **NRPS** | **1,145,167** | **1,189,833** | **prodigiosin** | **Polyketide** | **12%** |
| thiopeptide | 1,727,664 | 1,754,102 | O-antigen | Saccharide | 14% |
| NRPS | 2,309,939 | 2,356,426 | colicin V | RiPP | 1% |
| RRE-containing | 2,427,864 | 2,448,142 | lankacidin C | NRP + Polyketide | 13% |
| NRPS | 2,465,875 | 2,511,492 | - | - | - |
| NRPS | 4,473,726 | 4,521,571 | syringopeptin 25A | NRP | 100% |
| NRPS,T1PKS | 4,756,503 | 4,811,124 | - | - | -- |
| *Serratia marcescens* strain ICU-3 chromosome | | | | | |
| betalactone | 63,175 | 88,843 | - | - | - |
| **NRPS** | **438,566** | **483,230** | **prodigiosin** | **Polyketide** | **12%** |
| thiopeptide | 1,056,133 | 1,082,571 | O-antigen | Saccharide | 14% |
| NRPS | 1,638,146 | 1,684,531 | colicin V | RiPP | 1% |

| | | | | | |
|---|---|---|---|---|---|
| RRE-containing | 1,759,572 | 1,779,850 | lankacidin C | NRP + Polyketide | 13% |
| NRPS | 2,495,657 | 2,543,793 | - | - | - |
| NRPS | 3,776,395 | 3,830,663 | thanamycin | NRP:Beta-lactam | 18% |
| NRPS,T1PKS | 4,060,517 | 4,114,670 | - | - | - |
| hserlactone | 4,368,517 | 4,389,191 | - | - | |
| NRPS | 4,643,966 | 4,687,910 | turnerbactin | NRP | 23% |
| NRPS-like | 4,843,178 | 4,886,165 | - | - | - |
| *Serratia marcescens* strain ICU-4 chromosome | | | | | |
| betalactone | 62,962 | 88,630 | - | - | - |
| **NRPS** | **438,302** | **483,362** | **prodigiosin** | **Polyketide** | **12%** |
| thiopeptide | 1,017,929 | 1,044,367 | - | - | - |
| NRPS | 1,600,569 | 1,645,273 | colicin V | RiPP | 1% |
| RRE-containing | 1,720,103 | 1,740,381 | lankacidin C | NRP + Polyketide | 20% |
| NRPS | 3,729,192 | 3,782,018 | Le-pyrrolopyrazines | NRP | 27% |
| NRPS | 4,010,500 | 4,065,120 | - | - | - |
| hserlactone | 4,395,476 | 4,416,150 | - | - | - |
| NRPS-like | 4,848,420 | 4,890,712 | - | - | - |
| NRPS-like | 5,183,622 | 5,209,716 | nannocystin a | NRP + Polyketide | 21% |
| *Serratia marcescens* strain JW-CZ2 chromosome, complete genome | | | | | |
| NRPS | 628,929 | 671,919 | xantholipin | Polyketide | 4% |
| NRPS | 2,577,090 | 2,623,679 | pyrronazol B | NRP + Polyketide | 9% |
| redox-cofactor | 2,658,049 | 2,680,211 | lankacidin C | NRP + Polyketide | 13% |
| NRPS | 2,761,695 | 2,807,568 | microcin H47 | RiPP:Microcin | 20% |
| thiopeptide | 3,365,828 | 3,392,272 | O-antigen | Saccharide | 14% |
| **prodigiosin** | **3,972,294** | **4,007,314** | **prodigiosin** | **Polyketide** | **100%** |
| betalactone | 4,352,236 | 4,377,906 | - | - | - |
| hserlactone,NRPS-like | 4,556,975 | 4,608,541 | - | - | - |
| NRPS | 4,730,868 | 4,790,691 | vulnibactin | NRP | 18% |
| *Serratia marcescens* strain KS10 chromosome | | | | | |
| NRPS | 72,422 | 115,598 | xantholipin | Polyketide | 4% |

| | | | | | |
|---|---|---|---|---|---|
| NRPS | 1,457,499 | 1,501,253 | - | - | - |
| NRPS | 2,147,430 | 2,193,947 | pyrronazol B | NRP + Polyketide | 9% |
| redox-cofactor | 2,213,809 | 2,234,868 | lankacidin C | NRP + Polyketide | 13% |
| thiopeptide | 2,987,155 | 3,013,599 | O-antigen | Saccharide | 14% |
| **prodigiosin** | **3,605,793** | **3,640,813** | **prodigiosin** | **Polyketide** | **100%** |
| redox-cofactor | 3,793,379 | 3,815,541 | lankacidin C | NRP + Polyketide | 13% |
| betalactone | 3,986,079 | 4,011,749 | - | - | - |
| NRPS | 4,400,917 | 4,460,758 | vulnibactin | NRP | 18% |
| *Serratia marcescens* strain LVF3 chromosome, complete genome | | | | | |
| NRPS | 270,894 | 329,117 | vulnibactin | NRP | 18% |
| hserlactone | 576,985 | 597,677 | - | - | - |
| betalactone | 787,022 | 812,711 | | - | - |
| thiopeptide | 1,859,438 | 1,885,880 | O-antigen | Saccharide | 14% |
| RRE-containing | 2,655,355 | 2,675,633 | lankacidin C | NRP + Polyketide | 13% |
| T1PKS,NRPS | 2,682,525 | 2,749,394 | pyrronazol B | NRP + Polyketide | 9% |
| NRPS | 3,404,896 | 3,448,585 | - | - | - |
| NRPS-like,arylpolyene | 3,478,555 | 3,526,663 | andrimid | NRP:Beta-lactam + Polyketide:Type II | 95% |
| T1PKS | 4,417,765 | 4,458,747 | - | - | - |
| siderophore | 4,658,418 | 4,670,277 | - | - | - |
| NRPS | 4,861,191 | 4,914,409 | lokisin | NRP | 21% |
| NRPS,T1PKS | 5,146,930 | 5,201,552 | - | - | - |
| *Serratia marcescens* strain WVU-002 chromosome, complete genome | | | | | |
| NRPS | 262,689 | 321,011 | vulnibactin | NRP | 18% |
| NRPS-like | 453,528 | 494,770 | - | - | - |
| betalactone | 711,400 | 737,069 | - | - | - |
| NRPS,T1PKS | 1,223,026 | 1,278,651 | olimycin A / olimycin B | Polyketide | 5% |
| thiopeptide | 1,851,898 | 1,878,341 | O-antigen | Saccharide | 14% |
| RRE-containing | 2,609,714 | 2,629,992 | lankacidin C | NRP + Polyketide | 13% |
| NRPS | 2,651,206 | 2,698,989 | pyrronazol B | NRP + Polyketide | 9% |

| | | | | | |
|---|---|---|---|---|---|
| NRPS | 3,340,206 | 3,425,890 | - | - | - |
| NRPS | 4,749,364 | 4,806,970 | xenotetrapeptide | NRP | 100% |
| *Serratia marcescens* strain WVU-004 chromosome, complete genome | | | | | |
| NRPS | 262,725 | 321,047 | vulnibactin | NRP | 18% |
| NRPS-like | 453,564 | 494,806 | - | - | - |
| betalactone | 711,436 | 737,105 | - | - | - |
| NRPS,T1PKS | 1,223,062 | 1,278,687 | olimycin A / olimycin B | Polyketide | 5% |
| thiopeptide | 1,851,933 | 1,878,376 | O-antigen | Saccharide | 14% |
| RRE-containing | 2,609,750 | 2,630,028 | lankacidin C | NRP + Polyketide | 13% |
| NRPS | 2,651,242 | 2,699,025 | pyrronazol B | NRP + Polyketide | 9% |
| NRPS | 3,340,242 | 3,425,926 | - | - | - |
| NRPS | 4,749,400 | 4,807,039 | xenotetrapeptide | NRP | 100% |
| *Serratia marcescens* strain WVU-007 chromosome, complete genome | | | | | |
| NRPS | 262,733 | 321,048 | vulnibactin | NRP | 18% |
| NRPS-like | 453,565 | 494,807 | - | - | |
| betalactone | 711,437 | 737,106 | - | - | - |
| NRPS,T1PKS | 1,222,702 | 1,278,328 | olimycin A / olimycin B | Polyketide | 5% |
| thiopeptide | 1,851,576 | 1,878,019 | O-antigen | Saccharide | 14% |
| RRE-containing | 2,643,878 | 2,664,156 | lankacidin C | NRP + Polyketide | 13% |
| NRPS | 2,685,346 | 2,733,129 | pyrronazol B | NRP + Polyketide | 9% |
| NRPS | 3,374,346 | 3,460,030 | - | - | - |
| NRPS | 4,766,976 | 4,824,828 | xenotetrapeptide | NRP | 100% |
| *Serratia marcescens* strain WVU-008 chromosome, complete genome | | | | | |
| betalactone | 724,883 | 750,552 | - | - | - |
| thiopeptide | 1,856,534 | 1,882,971 | O-antigen | Saccharide | 14% |
| RRE-containing | 2,589,923 | 2,610,201 | lankacidin C | NRP + Polyketide | 13% |
| NRPS | 2,631,882 | 2,679,201 | pyrronazol B | NRP + Polyketide | 9% |
| NRPS | 3,357,966 | 3,403,499 | | | |
| NRPS | 4,705,706 | 4,760,170 | thanamycin | NRP:Beta-lactam | 18% |
| *Serratia marcescens* strain WVU-009 chromosome, complete genome | | | | | |

| | | | | | |
|---|---|---|---|---|---|
| hserlactone | 23,292 | 43,966 | - | - | - |
| NRPS | 290,044 | 347,904 | vulnibactin | NRP | 12% |
| betalactone | 729,231 | 754,899 | - | - | - |
| **NRPS** | **1,124,997** | **1,170,206** | **prodigiosin** | **Polyketide** | **12%** |
| thiopeptide | 1,790,895 | 1,816,576 | O-antigen | Saccharide | 14% |
| RRE-containing | 2,508,330 | 2,528,608 | lankacidin C | NRP + Polyketide | 13% |
| T1PKS | 2,555,673 | 2,598,552 | pyrronazol B | NRP + Polyketide | 9% |
| NRPS | 3,229,609 | 3,315,384 | - | - | - |
| thiopeptide,LAP,NRPS | 3,819,912 | 3,867,256 | microcin E492 | RiPP:Microcin | 12% |
| NRPS | 4,751,178 | 4,809,024 | xenotetrapeptide | NRP | 100% |
| *Serratia marcescens* strain WVU-010 chromosome, complete genome | | | | | |
| NRPS | 262,701 | 321,016 | vulnibactin | NRP | 18% |
| NRPS-like | 453,533 | 494,775 | - | - | - |
| betalactone | 711,405 | 737,074 | - | - | - |
| NRPS,T1PKS | 1,222,671 | 1,278,297 | olimycin A / olimycin B | Polyketide | 5% |
| thiopeptide | 1,851,546 | 1,877,989 | O-antigen | Saccharide | 14% |
| RRE-containing | 2,643,847 | 2,664,125 | lankacidin C | NRP + Polyketide | 13% |
| NRPS | 2,685,315 | 2,733,098 | pyrronazol B | NRP + Polyketide | 9% |
| NRPS | 3,374,315 | 3,459,999 | - | - | - |
| NRPS | 4,766,945 | 4,824,797 | xenotetrapeptide | NRP | 100% |
| *Serratia marcescens* strain YHYF1 chromosome, complete genome | | | | | |
| NRPS | 263,161 | 321,984 | vulnibactin | NRP | 18% |
| NRPS-like,hserlactone | 525,454 | 574,703 | - | - | - |
| betalactone | 781,253 | 806,923 | - | - | - |
| **prodigiosin** | **1,150,979** | **1,186,005** | **prodigiosin** | **Polyketide** | **100%** |
| hserlactone | 1,213,650 | 1,232,702 | olimycin A / olimycin B | Polyketide | 5% |
| thiopeptide | 1,825,114 | 1,851,558 | O-antigen | Saccharide | 14% |
| NRPS | 2,447,774 | 2,493,653 | microcin H47 | RiPP:Microcin | 20% |
| redox-cofactor | 2,570,197 | 2,592,359 | lankacidin C | NRP + Polyketide | 13% |
| NRPS | 2,623,653 | 2,671,574 | pyrronazol B | NRP + Polyketide | 9% |

| | | | | | |
|---|---|---|---|---|---|
| hserlactone | 3,743,913 | 3,764,545 | - | - | - |
| NRPS | 4,745,728 | 4,789,663 | xantholipin | Polyketide | 4% |
| *Serratia marcescens subsp. marcescens* ATCC 13880 chromosome, complete genome | | | | | |
| NRPS | 1,114,925 | 1,158,447 | xantholipin | Polyketide | 4% |
| NRPS | 1,954,807 | 2,013,424 | vulnibactin | NRP | 12% |
| NRPS-like | 2,135,877 | 2,175,997 | - | - | - |
| betalactone | 2,413,728 | 2,439,399 | - | - | - |
| **prodigiosin** | **2,794,945** | **2,829,965** | **prodigiosin** | **Polyketide** | **100%** |
| thiopeptide | 3,423,043 | 3,449,487 | O-antigen | Saccharide | 14% |
| NRPS | 4,023,898 | 4,070,909 | microcin H47 | RiPP:Microcin | 20% |
| redox-cofactor | 4,145,815 | 4,167,977 | lankacidin C | NRP + Polyketide | 13% |
| NRPS | 4,197,325 | 4,245,835 | pyrronazol B | NRP + Polyketide | 9% |
| *Serratia marcescens subsp. marcescens* ATCC 13880 chromosome, complete genome | | | | | |
| NRPS | 299,774 | 358,408 | vulnibactin | NRP | 12% |
| NRPS-like | 480,866 | 520,990 | - | - | - |
| betalactone | 758,770 | 784,446 | - | - | - |
| **prodigiosin** | **1,140,027** | **1,175,047** | **prodigiosin** | **Polyketide** | **100%** |
| thiopeptide | 1,768,028 | 1,794,461 | O-antigen | Saccharide | 14% |
| NRPS-like,NRPS | 2,368,303 | 2,415,283 | microcin H47 | RiPP:Microcin | 20% |
| RRE-containing | 2,491,195 | 2,511,473 | lankacidin C | NRP + Polyketide | 20% |
| NRPS | 2,542,046 | 2,588,442 | pyrronazol B | NRP + Polyketide | 9% |
| NRPS | 4,575,382 | 4,619,182 | xantholipin | Polyketide | 4% |
| *Serratia marcescens subsp. marcescens* ATCC 13880 substr. Sm_S13_jyu2015 chromosome, complete genome | | | | | |
| NRPS | 299,729 | 358,346 | vulnibactin | NRP | 12% |
| NRPS-like | 480,799 | 522,880 | - | - | - |
| betalactone | 759,910 | 785,581 | - | - | - |
| **prodigiosin** | **1,141,128** | **1,176,148** | **prodigiosin** | **Polyketide** | **100%** |
| thiopeptide | 1,769,229 | 1,795,673 | O-antigen | Saccharide | 14% |
| NRPS | 2,370,087 | 2,417,098 | microcin H47 | RiPP:Microcin | 20% |
| redox-cofactor | 2,492,004 | 2,514,166 | lankacidin C | NRP + Polyketide | 13% |

| | | | | | |
|---|---|---|---|---|---|
| NRPS | 2,544,015 | 2,590,434 | pyrronazol B | NRP + Polyketide | 9% |
| NRPS | 4,578,309 | 4,622,247 | xantholipin | Polyketide | 4% |
| *Serratia marcescens subsp. marcescens* ATCC 13880 substr. Sm_S22_jyu2015 chromosome, complete genome | | | | | |
| NRPS | 299,729 | 358,346 | vulnibactin | NRP | 12% |
| NRPS-like | 480,799 | 520,920 | - | - | - |
| betalactone | 758,651 | 784,322 | - | - | - |
| **prodigiosin** | **1,139,869** | **1,174,889** | **prodigiosin** | **Polyketide** | **100%** |
| thiopeptide | 1,767,991 | 1,794,435 | O-antigen | Saccharide | 14% |
| NRPS | 2,368,848 | 2,415,859 | microcin H47 | RiPP:Microcin | 20% |
| redox-cofactor | 2,490,765 | 2,512,927 | lankacidin C | NRP + Polyketide | 13% |
| NRPS | 2,542,776 | 2,589,195 | pyrronazol B | NRP + Polyketide | 9% |
| NRPS | 4,577,070 | 4,621,008 | xantholipin | Polyketide | 4% |
| *Serratia marcescens subsp. marcescens* ATCC 13880 substr. Sm_S24_jyu2015 chromosome, complete genome | | | | | |
| NRPS | 299,728 | 358,345 | vulnibactin | NRP | 12% |
| NRPS-like | 480,798 | 522,879 | - | - | - |
| betalactone | 759,909 | 785,580 | - | - | - |
| **prodigiosin** | **1,141,127** | **1,176,147** | **prodigiosin** | **Polyketide** | **100%** |
| thiopeptide | 1,769,228 | 1,795,672 | O-antigen | Saccharide | 14% |
| NRPS | 2,370,085 | 2,417,096 | microcin H47 | RiPP:Microcin | 20% |
| redox-cofactor | 2,492,002 | 2,514,164 | lankacidin C | NRP + Polyketide | 13% |
| NRPS | 2,544,013 | 2,590,432 | pyrronazol B | NRP + Polyketide | 9% |
| NRPS | 4,578,307 | 4,622,245 | xantholipin | Polyketide | 4% |
| *Serratia marcescens subsp. marcescens* ATCC 13880 substr. Sm_S28_jyu2015 chromosome, complete genome | | | | | |
| NRPS | 299,728 | 358,345 | vulnibactin | NRP | 12% |
| NRPS-like | 480,799 | 520,920 | - | - | - |
| betalactone | 758,651 | 784,322 | - | - | - |
| **prodigiosin** | **1,139,869** | **1,174,889** | **prodigiosin** | **Polyketide** | **100%** |
| thiopeptide | 1,767,970 | 1,794,414 | O-antigen | Saccharide | 14% |
| NRPS | 2,368,827 | 2,415,838 | microcin H47 | RiPP:Microcin | 20% |
| redox-cofactor | 2,490,744 | 2,512,906 | lankacidin C | NRP + Polyketide | 13% |

| | | | | | |
|---|---|---|---|---|---|
| NRPS | 2,542,755 | 2,589,174 | pyrronazol B | NRP + Polyketide | 9% |
| NRPS | 4,577,049 | 4,620,987 | xantholipin | Polyketide | 4% |
| *Serratia marcescens subsp. marcescens* ATCC 13880 substr. Sm_S33_jyu2015 chromosome, complete genome | | | | | |
| NRPS | 299,729 | 358,346 | vulnibactin | NRP | 12% |
| NRPS-like | 480,799 | 520,920 | - | - | - |
| betalactone | 758,651 | 784,322 | - | - | - |
| **prodigiosin** | **1,139,869** | **1,174,889** | **prodigiosin** | **Polyketide** | **100%** |
| thiopeptide | 1,767,971 | 1,794,415 | O-antigen | Saccharide | 14% |
| NRPS | 2,368,828 | 2,415,839 | microcin H47 | RiPP:Microcin | 20% |
| redox-cofactor | 2,490,745 | 2,512,907 | lankacidin C | NRP + Polyketide | 13% |
| NRPS | 2,542,756 | 2,589,175 | pyrronazol B | NRP + Polyketide | 9% |
| NRPS | 4,577,050 | 4,620,988 | xantholipin | Polyketide | 4% |
| *Serratia marcescens subsp. marcescens* ATCC 13880 substr. Sm_S37_jyu2015 chromosome, complete genome | | | | | |
| NRPS | 299,728 | 358,345 | vulnibactin | NRP | 12% |
| NRPS-like | 480,798 | 520,919 | - | - | - |
| betalactone | 758,650 | 784,321 | - | - | - |
| **prodigiosin** | **1,139,868** | **1,174,888** | **prodigiosin** | **Polyketide** | **100%** |
| thiopeptide | 1,767,969 | 1,794,413 | O-antigen | Saccharide | 14% |
| NRPS | 2,368,826 | 2,415,837 | microcin H47 | RiPP:Microcin | 20% |
| redox-cofactor | 2,490,743 | 2,512,905 | lankacidin C | NRP + Polyketide | 13% |
| NRPS | 2,542,754 | 2,589,173 | pyrronazol B | NRP + Polyketide | 9% |
| NRPS | 4,577,039 | 4,620,977 | xantholipin | Polyketide | 4% |
| *Serratia marcescens subsp. marcescens* ATCC 13880 substr. Sm_S57_jyu2015 chromosome, complete genome | | | | | |
| NRPS | 299,729 | 358,346 | vulnibactin | NRP | 12% |
| NRPS-like | 480,799 | 520,920 | - | - | - |
| betalactone | 758,651 | 784,322 | - | - | - |
| **prodigiosin** | **1,139,869** | **1,174,889** | **prodigiosin** | **Polyketide** | **100%** |
| thiopeptide | 1,767,970 | 1,794,414 | O-antigen | Saccharide | 14% |
| NRPS | 2,368,828 | 2,415,839 | microcin H47 | RiPP:Microcin | 20% |
| redox-cofactor | 2,490,745 | 2,512,907 | lankacidin C | NRP + Polyketide | 13% |

| | | | | | |
|---|---|---|---|---|---|
| NRPS | 2,542,756 | 2,589,175 | pyrronazol B | NRP + Polyketide | 9% |
| NRPS | 4,577,050 | 4,620,988 | xantholipin | Polyketide | 4% |
| *Serratia marcescens subsp. marcescens* ATCC 13880 substr. Sm_S60_jyu2015 chromosome, complete genome | | | | | |
| NRPS | 299,729 | 358,346 | vulnibactin | NRP | 12% |
| NRPS-like | 480,799 | 520,920 | - | - | - |
| betalactone | 758,651 | 784,322 | - | - | - |
| **prodigiosin** | **1,139,869** | **1,174,889** | **prodigiosin** | **Polyketide** | **100%** |
| thiopeptide | 1,769,249 | 1,795,693 | O-antigen | Saccharide | 14% |
| NRPS | 2,370,106 | 2,417,117 | microcin H47 | RiPP:Microcin | 20% |
| redox-cofactor | 2,492,023 | 2,514,185 | lankacidin C | NRP + Polyketide | 13% |
| NRPS | 2,544,034 | 2,590,453 | pyrronazol B | NRP + Polyketide | 9% |
| NRPS | 4,578,328 | 4,622,266 | xantholipin | Polyketide | 4% |
| *Serratia marcescens subsp. marcescens* ATCC 13880 substr. Sm_S64_jyu2015 chromosome, complete genome | | | | | |
| NRPS | 299,729 | 358,346 | vulnibactin | NRP | 12% |
| NRPS-like | 480,799 | 520,920 | - | - | - |
| betalactone | 758,651 | 784,322 | - | - | - |
| **prodigiosin** | **1,139,869** | **1,174,889** | **prodigiosin** | **Polyketide** | **100%** |
| thiopeptide | 1,767,969 | 1,794,413 | O-antigen | Saccharide | 14% |
| NRPS | 2,368,825 | 2,415,836 | microcin H47 | RiPP:Microcin | 20% |
| redox-cofactor | 2,490,742 | 2,512,904 | lankacidin C | NRP + Polyketide | 13% |
| NRPS | 2,542,753 | 2,589,172 | pyrronazol B | NRP + Polyketide | 9% |
| NRPS | 4,577,047 | 4,620,985 | xantholipin | Polyketide | 4% |
| *Serratia marcescens subsp. marcescens* ATCC 13880 substr. Sm_S65_jyu2015 chromosome, complete genome | | | | | |
| NRPS | 299,729 | 358,346 | vulnibactin | NRP | 12% |
| NRPS-like | 480,799 | 520,920 | - | - | - |
| betalactone | 758,651 | 784,322 | - | - | - |
| **prodigiosin** | **1,139,869** | **1,174,889** | **prodigiosin** | **Polyketide** | **100%** |
| thiopeptide | 1,767,970 | 1,794,414 | O-antigen | Saccharide | 14% |
| NRPS | 2,368,827 | 2,415,838 | microcin H47 | RiPP:Microcin | 20% |
| redox-cofactor | 2,490,744 | 2,512,906 | lankacidin C | NRP + Polyketide | 13% |

| | | | | | |
|---|---|---|---|---|---|
| NRPS | 2,542,755 | 2,589,174 | pyrronazol B | NRP + Polyketide | 9% |
| NRPS | 4,577,049 | 4,620,987 | xantholipin | Polyketide | 4% |
| *Serratia marcescens subsp. marcescens* ATCC 13880 substr. Sm_S67_jyu2015 chromosome, complete genome | | | | | |
| NRPS | 299,730 | 358,347 | vulnibactin | NRP | 12% |
| NRPS-like | 480,800 | 520,921 | - | - | - |
| betalactone | 758,652 | 784,323 | - | - | - |
| **prodigiosin** | **1,139,870** | **1,174,890** | **prodigiosin** | **Polyketide** | **100%** |
| thiopeptide | 1,767,971 | 1,794,415 | O-antigen | Saccharide | 14% |
| NRPS | 2,368,828 | 2,415,839 | microcin H47 | RiPP:Microcin | 20% |
| redox-cofactor | 2,490,745 | 2,512,907 | lankacidin C | NRP + Polyketide | 13% |
| NRPS | 2,542,756 | 2,589,175 | pyrronazol B | NRP + Polyketide | 9% |
| NRPS | 4,577,050 | 4,620,988 | xantholipin | Polyketide | 4% |
| *Serratia marcescens* strain BP2 chromosome, complete genome | | | | | |
| NRPS | 317,234 | 375,724 | vulnibactin | NRP | 18% |
| betalactone | 794,673 | 820,343 | - | - | - |
| redox-cofactor | 1,023,821 | 1,044,475 | lankacidin C | NRP + Polyketide | 13% |
| **NRPS,prodigiosin** | **1,183,642** | **1,244,504** | **prodigiosin** | **Polyketide** | **100%** |
| thiopeptide | 1,825,954 | 1,852,398 | O-antigen | Saccharide | 14% |
| redox-cofactor | 2,516,897 | 2,539,059 | lankacidin C | NRP + Polyketide | 13% |
| NRPS | 2,553,833 | 2,602,040 | pyrronazol B | NRP + Polyketide | 9% |
| NRPS | 3,237,153 | 3,280,921 | - | - | - |
| arylpolyene | 4,549,763 | 4,593,359 | aryl polyenes | Other | 100% |
| NRPS | 4,600,446 | 4,644,384 | xantholipin | Polyketide | 4% |
| *Serratia marcescens* strain BWH-23 chromosome, complete genome | | | | | |
| hserlactone | 488,965 | 509,657 | - | - | - |
| betalactone | 692,180 | 717,848 | - | - | - |
| thiopeptide | 1,666,597 | 1,693,040 | O-antigen | Saccharide | 14% |
| NRPS | 2,286,156 | 2,333,168 | microcin H47 | RiPP:Microcin | 20% |
| RRE-containing | 2,424,572 | 2,444,850 | lankacidin C | NRP + Polyketide | 13% |
| NRPS | 2,466,729 | 2,513,234 | pyrronazol B | NRP + Polyketide | 9% |

| | | | | | |
|---|---|---|---|---|---|
| T1PKS,NRPS | 3,072,479 | 3,134,476 | althiomycin | NRP | 100% |
| NRPS | 4,516,202 | 4,573,982 | rhizomide A / rhizomide B / rhizomide C | NRP | 100% |
| *Serratia marcescens* strain BWH-35 chromosome, complete genome | | | | | |
| NRPS | 267,647 | 326,056 | turnerbactin | NRP | 30% |
| NRPS-like | 440,636 | 482,648 | - | - | - |
| betalactone | 685,071 | 710,741 | - | - | - |
| thiopeptide | 1,794,394 | 1,820,836 | O-antigen | Saccharide | 14% |
| siderophore | 1,970,686 | 1,982,545 | - | - | - |
| redox-cofactor | 2,540,914 | 2,563,076 | lankacidin C | NRP + Polyketide | 13% |
| T1PKS,NRPS | 3,235,806 | 3,298,743 | althiomycin | NRP | 100% |
| NRPS | 3,425,499 | 3,470,698 | - | - | - |
| NRPS | 4,664,694 | 4,722,546 | rhizomide A / rhizomide B / rhizomide C | NRP | 100% |
| *Serratia marcescens* strain Byron chromosome, complete genome | | | | | |
| redox-cofactor | 381,296 | 402,268 | - | - | - |
| NRPS | 420,707 | 467,120 | pyrronazol B | NRP + Polyketide | 9% |
| NRPS | 1,137,641 | 1,181,410 | - | - | - |
| NRPS | 2,502,197 | 2,545,372 | xantholipin | Polyketide | 4% |
| NRPS | 3,322,988 | 3,382,806 | vulnibactin | NRP | 18% |
| hserlactone | 3,556,360 | 3,577,052 | - | - | - |
| betalactone | 3,776,439 | 3,802,109 | - | -- | - |
| redox-cofactor | 3,999,108 | 4,021,270 | lankacidin C | NRP + Polyketide | 13% |
| **prodigiosin** | **4,173,842** | **4,208,862** | **prodigiosin** | **Polyketide** | **100%** |
| thiopeptide | 4,813,843 | 4,840,287 | O-antigen | Saccharide | 14% |
| *Serratia marcescens* strain C110 chromosome, complete genome | | | | | |
| NRPS | 292,398 | 350,101 | vulnibactin | NRP | 18% |
| NRPS-like | 474,519 | 516,298 | - | - | - |
| betalactone | 772,672 | 798,341 | - | - | - |
| **NRPS** | **1,191,320** | **1,235,994** | **prodigiosin** | **Polyketide** | **12%** |
| thiopeptide | 1,833,432 | 1,859,873 | O-antigen | Saccharide | 14% |

| | | | | | |
|---|---|---|---|---|---|
| NRPS | 2,548,027 | 2,594,726 | colicin V | RiPP | 1% |
| RRE-containing | 2,679,397 | 2,699,675 | lankacidin C | NRP + Polyketide | 13% |
| NRPS | 2,719,864 | 2,765,007 | pyrronazol B | NRP + Polyketide | 9% |
| NRPS | 3,407,170 | 3,456,720 | lipopolysaccharide | Saccharide:Lipopolysaccharide | 5% |
| NRPS | 3,519,912 | 3,576,769 | gobichelin A / gobichelin B | NRP | 11% |
| RiPP-like | 3,918,458 | 3,931,004 | - | - | - |
| NRPS | 4,829,833 | 4,887,685 | rhizomide A / rhizomide B / rhizomide C | NRP | 100% |
| *Serratia marcescens* strain CM2012_028 chromosome, complete genome | | | | | |
| NRPS | 296,036 | 354,916 | vulnibactin | NRP | 18% |
| NRPS-like,hserlactone | 478,540 | 530,525 | - | - | - |
| betalactone | 722,031 | 747,701 | - | - | - |
| redox-cofactor | 924,975 | 946,345 | lankacidin C | NRP + Polyketide | 13% |
| **prodigiosin** | **1,108,577** | **1,143,597** | **prodigiosin** | **Polyketide** | **100%** |
| thiopeptide | 1,745,599 | 1,772,043 | O-antigen | Saccharide | 14% |
| NRPS | 2,314,745 | 2,360,618 | microcin H47 | RiPP:Microcin | 20% |
| redox-cofactor | 2,436,200 | 2,458,362 | lankacidin C | NRP + Polyketide | 13% |
| NRPS | 2,486,711 | 2,533,972 | pyrronazol B | NRP + Polyketide | 9% |
| NRPS | 4,501,602 | 4,545,537 | xantholipin | Polyketide | 4% |
| *Serratia marcescens* strain E28 chromosome, complete genome | | | | | |
| NRPS | 283,349 | 342,070 | vulnibactin | NRP | 12% |
| NRPS-like | 467,913 | 510,130 | - | - | - |
| betalactone | 713,591 | 739,261 | - | - | - |
| thiopeptide | 1,774,004 | 1,800,446 | O-antigen | Saccharide | 14% |
| NRPS | 2,378,990 | 2,426,161 | colicin V | RiPP | 1% |
| redox-cofactor | 2,499,931 | 2,522,093 | lankacidin C | NRP + Polyketide | 13% |
| T1PKS,NRPS | 3,228,504 | 3,335,305 | althiomycin | NRP | 87% |
| NRPS | 4,854,739 | 4,912,591 | rhizomide A / rhizomide B / rhizomide C | NRP | 100% |
| *Serratia marcescens* strain EL1 chromosome | | | | | |
| redox-cofactor | 517,325 | 538,384 | lankacidin C | NRP + Polyketide | 13% |

| | | | | | |
|---|---|---|---|---|---|
| NRPS | 558,246 | 604,763 | pyrronazol B | NRP + Polyketide | 9% |
| NRPS | 1,250,939 | 1,294,693 | - | - | - |
| NRPS | 2,636,589 | 2,679,765 | xantholipin | Polyketide | 4% |
| NRPS | 3,481,117 | 3,540,958 | vulnibactin | NRP | 18% |
| betalactone | 3,930,125 | 3,955,795 | - | - | - |
| redox-cofactor | 4,126,334 | 4,148,496 | lankacidin C | NRP + Polyketide | 13% |
| **prodigiosin** | **4,301,062** | **4,336,082** | **prodigiosin** | **Polyketide** | **100%** |
| thiopeptide | 4,928,283 | 4,954,727 | O-antigen | Saccharide | 14% |
| *Serratia marcescens* strain ESE2014 chromosome, complete genome | | | | | |
| NRPS | 297,033 | 355,150 | vulnibactin | NRP | 12% |
| NRPS-like | 478,197 | 520,531 | - | - | - |
| betalactone | 723,420 | 749,089 | - | - | - |
| **prodigiosin** | **1,098,550** | **1,133,570** | **prodigiosin** | **Polyketide** | **100%** |
| thiopeptide | 1,720,096 | 1,746,540 | O-antigen | Saccharide | 14% |
| NRPS | 2,335,218 | 2,382,230 | microcin H47 | RiPP:Microcin | 20% |
| redox-cofactor | 2,456,818 | 2,478,980 | lankacidin C | NRP + Polyketide | 13% |
| NRPS | 2,508,322 | 2,554,704 | pyrronazol B | NRP + Polyketide | 9% |
| NRPS | 4,520,162 | 4,564,100 | xantholipin | Polyketide | 4% |
| *Serratia marcescens* strain SJC1058 genome assembly, chromosome: 1 | | | | | |
| T1PKS,NRPS | 1 | 44,141 | althiomycin | NRP | 100% |
| NRPS | 109,836 | 154,929 | - | - | - |
| NRPS | 220,189 | 266,246 | yersiniabactin | NRP + Polyketide | 4% |
| NRPS | 1,595,548 | 1,653,111 | rhizomide A / rhizomide B / rhizomide C | NRP | 100% |
| NRPS | 2,501,455 | 2,561,446 | vulnibactin | NRP | 12% |
| NRPS-like | 2,684,756 | 2,727,737 | - | - | - |
| RRE-containing | 2,734,486 | 2,749,147 | - | - | - |
| betalactone | 2,955,862 | 2,981,531 | - | - | - |
| thiopeptide | 4,008,799 | 4,035,242 | O-antigen | Saccharide | 14% |
| siderophore | 4,220,889 | 4,232,748 | - | - | - |

| | | | | | |
|---|---|---|---|---|---|
| redox-cofactor | 4,803,993 | 4,826,155 | lankacidin C | NRP + Polyketide | 13% |
| *Serratia marcescens* strain SJC1061 genome assembly, chromosome: 1 | | | | | |
| betalactone | 783,606 | 809,275 | - | - | - |
| RRE-containing | 1,015,990 | 1,030,651 | - | - | - |
| NRPS-like | 1,037,826 | 1,080,013 | - | - | - |
| NRPS | 1,204,183 | 1,263,156 | vulnibactin | NRP | 12% |
| NRPS | 2,112,187 | 2,169,750 | rhizomide A / rhizomide B / rhizomide C | NRP | 100% |
| NRPS | 3,499,014 | 3,545,071 | yersiniabactin | NRP + Polyketide | 4% |
| NRPS | 3,610,331 | 3,655,424 | - | - | - |
| NRPS,T1PKS | 3,721,010 | 3,784,186 | althiomycin | NRP | 100% |
| redox-cofactor | 4,418,418 | 4,440,580 | lankacidin C | NRP + Polyketide | 13% |
| siderophore | 5,011,991 | 5,023,850 | - | - | - |
| thiopeptide | 5,209,496 | 5,235,939 | O-antigen | Saccharide | 14% |
| *Serratia marcescens* strain SJC1062 genome assembly, chromosome: 1 | | | | | |
| T1PKS,NRPS | 1 | 44,104 | althiomycin | NRP | 100% |
| NRPS | 109,799 | 154,892 | - | - | - |
| NRPS | 220,152 | 266,209 | yersiniabactin | NRP + Polyketide | 4% |
| NRPS | 1,597,119 | 1,653,075 | xenoamicin A / xenoamicin B | NRP:Cyclic depsipeptide | 20% |
| NRPS | 2,501,419 | 2,561,410 | vulnibactin | NRP | 12% |
| NRPS-like | 2,684,720 | 2,727,701 | - | - | - |
| RRE-containing | 2,734,450 | 2,749,111 | - | - | - |
| betalactone | 2,955,826 | 2,981,495 | - | - | - |
| thiopeptide | 4,008,768 | 4,035,211 | O-antigen | Saccharide | 14% |
| siderophore | 4,220,858 | 4,232,717 | - | - | - |
| redox-cofactor | 4,804,126 | 4,826,288 | lankacidin C | NRP + Polyketide | 13% |
| *Serratia marcescens* strain SJC1070 genome assembly, chromosome: 1 | | | | | |
| T1PKS,NRPS | 1 | 44,120 | althiomycin | NRP | 100% |
| NRPS | 109,815 | 154,908 | - | - | - |
| NRPS | 220,168 | 266,225 | yersiniabactin | NRP + Polyketide | 4% |

| | | | | | |
|---|---|---|---|---|---|
| NRPS | 1,595,597 | 1,653,160 | rhizomide A / rhizomide B / rhizomide C | NRP | 100% |
| NRPS | 2,501,504 | 2,561,495 | vulnibactin | NRP | 12% |
| NRPS-like | 2,684,805 | 2,727,786 | - | - | - |
| RRE-containing | 2,734,535 | 2,749,196 | - | - | - |
| betalactone | 2,955,911 | 2,981,580 | - | - | - |
| thiopeptide | 4,008,853 | 4,035,296 | O-antigen | Saccharide | 14% |
| siderophore | 4,220,943 | 4,232,802 | - | - | - |
| redox-cofactor | 4,804,214 | 4,826,376 | lankacidin C | NRP + Polyketide | 13% |
| *Serratia marcescens* strain SJC1039 genome assembly, chromosome: 1 | | | | | |
| thiopeptide | 254,427 | 280,868 | O-antigen | Saccharide | 14% |
| NRPS | 823,613 | 870,322 | colicin V | RiPP | 1% |
| RRE-containing | 938,792 | 959,070 | lankacidin C | NRP + Polyketide | 13% |
| NRPS | 977,344 | 1,023,806 | turnerbactin | NRP | 15% |
| NRPS | 1,638,664 | 1,687,777 | - | - | - |
| NRPS | 2,992,156 | 3,049,002 | rhizomide A / rhizomide B / rhizomide C | NRP | 100% |
| NRPS,T1PKS | 3,271,856 | 3,326,021 | - | - | - |
| NRPS | 3,836,196 | 3,894,716 | vulnibactin | NRP | 18% |
| NRPS-like | 4,008,260 | 4,050,681 | - | - | - |
| betalactone | 4,282,738 | 4,308,407 | - | - | - |
| **NRPS** | **4,649,548** | **4,695,256** | **prodigiosin** | **Polyketide** | **12%** |
| *Serratia marcescens* strain SJC1043 genome assembly, chromosome: 1 | | | | | |
| NRPS | 291,794 | 351,526 | vulnibactin | NRP | 12% |
| NRPS-like | 475,241 | 517,428 | - | - | - |
| RRE-containing | 524,603 | 539,264 | - | - | - |
| betalactone | 745,983 | 771,652 | - | - | - |
| thiopeptide | 1,842,231 | 1,868,674 | O-antigen | Saccharide | 14% |
| siderophore | 2,054,320 | 2,066,179 | - | - | - |
| redox-cofactor | 2,592,522 | 2,614,684 | lankacidin C | NRP + Polyketide | 13% |
| T1PKS,NRPS | 3,247,736 | 3,310,803 | althiomycin | NRP | 100% |

| | | | | | |
|---|---|---|---|---|---|
| NRPS | 3,376,498 | 3,421,591 | - | - | - |
| NRPS | 3,486,851 | 3,532,908 | yersiniabactin | NRP + Polyketide | 4% |
| NRPS | 4,820,546 | 4,875,176 | rhizomide A / rhizomide B / rhizomide C | NRP | 100% |
| *Serratia marcescens* strain SJC1044 genome assembly, chromosome: 1 | | | | | |
| thiopeptide | 249,745 | 276,188 | O-antigen | Saccharide | 14% |
| siderophore | 461,835 | 473,694 | - | - | - |
| redox-cofactor | 1,000,037 | 1,022,199 | lankacidin C | NRP + Polyketide | 13% |
| T1PKS,NRPS | 1,655,251 | 1,718,318 | althiomycin | NRP | 100% |
| NRPS | 1,784,013 | 1,829,106 | - | - | - |
| NRPS | 1,894,366 | 1,940,423 | yersiniabactin | NRP + Polyketide | 4% |
| NRPS | 3,229,611 | 3,282,345 | orfamide A / orfamide C | NRP:Cyclic depsipeptide | 17% |
| NRPS | 4,131,882 | 4,191,873 | vulnibactin | NRP | 12% |
| NRPS-like | 4,315,183 | 4,358,164 | - | - | - |
| RRE-containing | 4,362,811 | 4,383,077 | - | - | - |
| betalactone | 4,586,293 | 4,611,962 | - | - | - |
| *Serratia marcescens* strain SJC1045 genome assembly, chromosome: 1 | | | | | |
| thiopeptide | 249,682 | 276,125 | O-antigen | Saccharide | 14% |
| siderophore | 461,772 | 473,631 | - | - | - |
| redox-cofactor | 999,974 | 1,022,136 | lankacidin C | NRP + Polyketide | 13% |
| T1PKS,NRPS | 1,655,221 | 1,718,288 | althiomycin | NRP | 100% |
| NRPS | 1,783,983 | 1,829,076 | - | - | - |
| NRPS | 1,894,336 | 1,940,393 | yersiniabactin | NRP + Polyketide | 4% |
| NRPS | 3,228,025 | 3,282,366 | rhizomide A / rhizomide B / rhizomide C | NRP | 100% |
| NRPS | 4,131,903 | 4,191,894 | vulnibactin | NRP | 12% |
| NRPS-like | 4,315,204 | 4,358,185 | - | - | - |
| RRE-containing | 4,362,832 | 4,383,098 | - | - | - |
| betalactone | 4,586,314 | 4,611,983 | - | - | - |
| *Serratia marcescens* strain SJC1046 genome assembly, chromosome: 1 | | | | | |
| redox-cofactor | 653,517 | 675,679 | lankacidin C | NRP + Polyketide | 13% |

| | | | | | |
|---|---|---|---|---|---|
| siderophore | 1,244,161 | 1,256,020 | - | | |
| thiopeptide | 1,441,667 | 1,468,110 | O-antigen | Saccharide | 14% |
| betalactone | 2,499,957 | 2,525,626 | - | - | - |
| RRE-containing | 2,728,838 | 2,749,104 | - | - | - |
| NRPS-like | 2,754,177 | 2,796,364 | - | - | - |
| NRPS | 2,920,079 | 2,979,346 | vulnibactin | NRP | 12% |
| NRPS | 3,821,868 | 3,879,431 | rhizomide A / rhizomide B / rhizomide C | NRP | 100% |
| NRPS | 5,210,183 | 5,256,240 | yersiniabactin | NRP + Polyketide | 4% |
| NRPS | 5,321,500 | 5,366,593 | - | - | -- |
| NRPS,T1PKS | 5,432,179 | 5,476,370 | althiomycin | NRP | 100% |
| *Serratia marcescens subsp. marcescens* ATCC 13880 substr. Sm_S68_jyu2015 chromosome, complete genome | | | | | |
| NRPS | 299,728 | 358,345 | vulnibactin | NRP | 12% |
| NRPS-like | 480,798 | 520,919 | - | - | - |
| betalactone | 758,650 | 784,321 | - | - | - |
| **prodigiosin** | **1,139,868** | **1,174,888** | **prodigiosin** | **Polyketide** | **100%** |
| thiopeptide | 1,767,970 | 1,794,414 | O-antigen | Saccharide | 14% |
| NRPS | 2,368,827 | 2,415,838 | microcin H47 | RiPP:Microcin | 20% |
| redox-cofactor | 2,490,744 | 2,512,906 | lankacidin C | NRP + Polyketide | 13% |
| NRPS | 2,542,755 | 2,589,174 | pyrronazol B | NRP + Polyketide | 9% |
| NRPS | 4,577,049 | 4,620,987 | xantholipin | Polyketide | 4% |
| *Serratia marcescens subsp. marcescens* ATCC 13880 substr. Sm_S6_jyu2015 chromosome, complete genome | | | | | |
| NRPS | 299,729 | 358,346 | vulnibactin | NRP | 12% |
| NRPS-like | 480,799 | 520,920 | - | - | - |
| betalactone | 758,651 | 784,322 | - | - | - |
| **prodigiosin** | **1,139,869** | **1,174,889** | **prodigiosin** | **Polyketide** | **100%** |
| thiopeptide | 1,767,971 | 1,794,415 | O-antigen | Saccharide | 14% |
| NRPS | 2,368,828 | 2,415,839 | microcin H47 | RiPP:Microcin | 20% |

| | | | | | |
|---|---|---|---|---|---|
| redox-cofactor | 2,490,745 | 2,512,907 | lankacidin C | NRP + Polyketide | 13% |
| NRPS | 2,542,756 | 2,589,175 | pyrronazol B | NRP + Polyketide | 9% |
| NRPS | 4,577,050 | 4,620,988 | xantholipin | Polyketide | 4% |
| *Serratia marcescens subsp. marcescens* ATCC 13880 substr. Sm_S71_jyu2015 chromosome, complete genome | | | | | |
| NRPS | 299,729 | 358,346 | vulnibactin | NRP | 12% |
| NRPS-like | 480,799 | 520,920 | - | - | - |
| betalactone | 758,651 | 784,322 | - | - | - |
| **prodigiosin** | **1,139,869** | **1,174,889** | **prodigiosin** | **Polyketide** | **100%** |
| thiopeptide | 1,767,991 | 1,794,435 | O-antigen | Saccharide | 14% |
| NRPS | 2,368,848 | 2,415,859 | microcin H47 | RiPP:Microcin | 20% |
| redox-cofactor | 2,490,765 | 2,512,927 | lankacidin C | NRP + Polyketide | 13% |
| NRPS | 2,542,776 | 2,589,195 | pyrronazol B | NRP + Polyketide | 9% |
| NRPS | 4,577,070 | 4,621,008 | xantholipin | Polyketide | 4% |
| *Serratia marcescens subsp. marcescens* ATCC 13880 substr. Sm_S78_jyu2015 chromosome, complete genome | | | | | |
| NRPS | 299,730 | 358,347 | vulnibactin | NRP | 12% |
| NRPS-like | 480,800 | 520,921 | - | - | - |
| betalactone | 758,653 | 784,325 | - | - | - |
| **prodigiosin** | **1,139,872** | **1,174,892** | **prodigiosin** | **Polyketide** | **100%** |
| thiopeptide | 1,767,952 | 1,794,396 | O-antigen | Saccharide | 14% |
| NRPS | 2,368,809 | 2,415,821 | microcin E492 | RiPP:Microcin | 18% |
| redox-cofactor | 2,490,727 | 2,512,889 | lankacidin C | NRP + Polyketide | 13% |
| NRPS | 2,542,738 | 2,589,157 | pyrronazol B | NRP + Polyketide | 9% |
| NRPS | 4,577,032 | 4,620,970 | xantholipin | Polyketide | 4% |
| *Serratia marcescens subsp. marcescens* ATCC 13880 substr. Sm_S79_jyu2015 chromosome, complete genome | | | | | |
| NRPS | 299,728 | 358,345 | vulnibactin | NRP | 12% |
| NRPS-like | 480,798 | 522,879 | - | - | - |
| betalactone | 759,909 | 785,580 | - | - | - |

| | | | | | |
|---|---|---|---|---|---|
| **prodigiosin** | **1,141,127** | **1,176,147** | **prodigiosin** | **Polyketide** | **100%** |
| thiopeptide | 1,769,207 | 1,795,651 | O-antigen | Saccharide | 14% |
| NRPS | 2,370,064 | 2,417,075 | microcin H47 | RiPP:Microcin | 20% |
| redox-cofactor | 2,491,981 | 2,514,143 | lankacidin C | NRP + Polyketide | 13% |
| NRPS | 2,543,992 | 2,590,411 | pyrronazol B | NRP + Polyketide | 9% |
| NRPS | 4,578,286 | 4,622,224 | xantholipin | Polyketide | 4% |
| *Serratia marcescens subsp. marcescens* ATCC 13880 substr. Sm_S81_jyu2015 chromosome, complete genome | | | | | |
| NRPS | 299,729 | 358,346 | vulnibactin | NRP | 12% |
| NRPS-like | 480,799 | 520,920 | - | - | - |
| betalactone | 758,651 | 784,322 | - | - | - |
| **prodigiosin** | **1,139,870** | **1,174,890** | **prodigiosin** | **Polyketide** | **100%** |
| thiopeptide | 1,767,972 | 1,794,416 | O-antigen | Saccharide | 14% |
| NRPS | 2,368,829 | 2,415,840 | microcin H47 | RiPP:Microcin | 20% |
| redox-cofactor | 2,490,746 | 2,512,908 | lankacidin C | NRP + Polyketide | 13% |
| NRPS | 2,542,757 | 2,589,176 | pyrronazol B | NRP + Polyketide | 9% |
| NRPS | 4,577,051 | 4,620,989 | xantholipin | Polyketide | 4% |
| *Serratia marcescens subsp. marcescens* ATCC 13880 substr. Sm_S89_jyu2015 chromosome, complete genome | | | | | |
| NRPS | 299,729 | 358,346 | vulnibactin | NRP | 12% |
| NRPS-like | 480,799 | 520,920 | - | - | - |
| betalactone | 758,651 | 784,322 | - | - | - |
| **prodigiosin** | **1,139,870** | **1,174,890** | **prodigiosin** | **Polyketide** | **100%** |
| thiopeptide | 1,767,971 | 1,794,415 | O-antigen | Saccharide | 14% |
| NRPS | 2,368,828 | 2,415,839 | microcin H47 | RiPP:Microcin | 20% |
| redox-cofactor | 2,490,745 | 2,512,907 | lankacidin C | NRP + Polyketide | 13% |
| NRPS | 2,542,756 | 2,589,175 | pyrronazol B | NRP + Polyketide | 9% |
| NRPS | 4,577,050 | 4,620,988 | xantholipin | Polyketide | 4% |
| *Serratia marcescens subsp. marcescens* ATCC 13880 substr. Sm_S94_jyu2015 chromosome, complete genome | | | | | |

| | | | | | |
|---|---|---|---|---|---|
| NRPS | 299,729 | 358,346 | vulnibactin | NRP | 12% |
| NRPS-like | 480,799 | 520,920 | - | - | - |
| betalactone | 758,651 | 784,322 | - | - | - |
| **prodigiosin** | **1,139,869** | **1,174,889** | **prodigiosin** | **Polyketide** | **100%** |
| thiopeptide | 1,767,979 | 1,794,423 | O-antigen | Saccharide | 14% |
| NRPS | 2,368,836 | 2,415,847 | microcin H47 | RiPP:Microcin | 20% |
| redox-cofactor | 2,490,753 | 2,512,915 | lankacidin C | NRP + Polyketide | 13% |
| NRPS | 2,542,764 | 2,589,183 | pyrronazol B | NRP + Polyketide | 9% |
| NRPS | 4,577,058 | 4,620,996 | xantholipin | Polyketide | 4% |
| *Serratia marcescens subsp. marcescens* ATCC 13880 substr. Sm_S95_jyu2015 chromosome, complete genome | | | | | |
| NRPS | 299,729 | 358,346 | vulnibactin | NRP | 12% |
| NRPS-like | 482,058 | 522,179 | - | - | - |
| betalactone | 759,910 | 785,581 | - | - | - |
| **prodigiosin** | **1,141,128** | **1,176,148** | **prodigiosin** | **Polyketide** | **100%** |
| thiopeptide | 1,769,250 | 1,795,694 | O-antigen | Saccharide | 14% |
| NRPS | 2,370,107 | 2,417,118 | microcin H47 | RiPP:Microcin | 20% |
| redox-cofactor | 2,492,024 | 2,514,186 | lankacidin C | NRP + Polyketide | 13% |
| NRPS | 2,544,035 | 2,590,454 | pyrronazol B | NRP + Polyketide | 9% |
| NRPS | 4,578,329 | 4,622,267 | xantholipin | Polyketide | 4% |
| *Serratia marcescens subsp. marcescens* ATCC 13880 substr. Sm_S96_jyu2015 chromosome, complete genome | | | | | |
| NRPS | 299,730 | 358,347 | vulnibactin | NRP | 12% |
| NRPS-like | 480,800 | 520,921 | - | - | - |
| betalactone | 758,652 | 784,323 | - | - | - |
| **prodigiosin** | **1,139,870** | **1,174,890** | **prodigiosin** | **Polyketide** | **100%** |
| thiopeptide | 1,767,971 | 1,794,415 | O-antigen | Saccharide | 14% |
| NRPS | 2,368,828 | 2,415,839 | microcin H47 | RiPP:Microcin | 20% |
| redox-cofactor | 2,490,745 | 2,512,907 | lankacidin C | NRP + Polyketide | 13% |

| | | | | | |
|---|---|---|---|---|---|
| NRPS | 2,542,756 | 2,589,175 | pyrronazol B | NRP + Polyketide | 9% |
| NRPS | 4,577,050 | 4,620,988 | xantholipin | Polyketide | 4% |
| *Serratia marcescens* strain SCH909 chromosome, complete genome | | | | | |
| NRPS,T1PKS | 273,948 | 359,632 | vulnibactin | NRP | 12% |
| NRPS-like | 473,624 | 515,317 | - | - | - |
| betalactone | 735,092 | 760,761 | - | - | - |
| thiopeptide | 1,902,742 | 1,929,183 | O-antigen | Saccharide | 14% |
| RRE-containing | 2,616,697 | 2,636,975 | lankacidin C | NRP + Polyketide | 13% |
| NRPS | 2,651,856 | 2,698,162 | - | - | - |
| NRPS | 3,347,252 | 3,397,034 | - | - | - |
| NRPS | 4,733,280 | 4,791,132 | xenotetrapeptide | NRP | 100% |
| *Serratia marcescens* strain SCQ1 chromosome, complete genome | | | | | |
| NRPS | 821,856 | 865,151 | xantholipin | Polyketide | 4% |
| NRPS | 1,637,989 | 1,696,809 | vulnibactin | NRP | 18% |
| NRPS-like | 1,820,133 | 1,861,342 | - | - | - |
| betalactone | 2,063,158 | 2,088,830 | - | - | - |
| **prodigiosin** | **2,434,448** | **2,469,468** | **prodigiosin** | **Polyketide** | **100%** |
| thiopeptide | 3,055,132 | 3,081,576 | O-antigen | Saccharide | 14% |
| NRPS | 3,631,603 | 3,677,389 | microcin H47 | RiPP:Microcin | 20% |
| RRE-containing | 3,756,132 | 3,776,410 | lankacidin C | NRP + Polyketide | 13% |
| NRPS | 3,805,939 | 3,854,449 | pyrronazol B | NRP + Polyketide | 9% |
| *Serratia marcescens* strain SGAir0764 chromosome, complete genome | | | | | |
| NRPS | 271,596 | 330,471 | vulnibactin | NRP | 18% |
| NRPS-like | 566,069 | 607,775 | - | - | - |
| betalactone | 805,220 | 830,890 | - | - | - |
| **prodigiosin** | **1,159,270** | **1,194,290** | **prodigiosin** | **Polyketide** | **100%** |
| thiopeptide | 1,763,450 | 1,789,894 | O-antigen | Saccharide | 14% |

| | | | | | |
|---|---|---|---|---|---|
| NRPS | 2,348,426 | 2,392,145 | microcin H47 | RiPP:Microcin | 20% |
| redox-cofactor | 2,469,253 | 2,491,415 | lankacidin C | NRP + Polyketide | 13% |
| NRPS | 2,516,490 | 2,562,713 | pyrronazol B | NRP + Polyketide | 9% |
| NRPS | 4,574,971 | 4,618,909 | xantholipin | Polyketide | 4% |
| RiPP-like | 4,929,853 | 4,942,384 | - | - | - |
| *Serratia marcescens* strain SMBC50 chromosome, complete genome | | | | | |
| NRPS | 267,723 | 325,426 | vulnibactin | NRP | 18% |
| NRPS-like | 449,844 | 491,623 | - | - | - |
| betalactone | 747,997 | 773,666 | - | - | - |
| **NRPS** | **1,166,644** | **1,211,318** | **prodigiosin** | **Polyketide** | **12%** |
| thiopeptide | 1,808,511 | 1,834,952 | O-antigen | Saccharide | 14% |
| NRPS | 2,558,492 | 2,605,191 | colicin V | RiPP | 1% |
| RRE-containing | 2,689,862 | 2,710,140 | lankacidin C | NRP + Polyketide | 13% |
| NRPS | 2,730,329 | 2,775,472 | pyrronazol B | NRP + Polyketide | 9% |
| NRPS | 3,452,973 | 3,502,523 | lipopolysaccharide | Saccharide:Lipopolysaccharide | 5% |
| NRPS | 3,565,715 | 3,622,572 | gobichelin A / gobichelin B | NRP | 11% |
| RiPP-like | 3,962,059 | 3,974,605 | - | - | - |
| NRPS | 4,570,125 | 4,627,977 | rhizomide A / rhizomide B / rhizomide C | NRP | 100% |
| *Serratia marcescens* strain SMNSF-1 chromosome, complete genome | | | | | |
| NRPS | 298,033 | 356,999 | vulnibactin | NRP | 18% |
| NRPS-like | 485,301 | 524,943 | - | - | - |
| betalactone | 783,151 | 808,821 | - | - | - |
| **prodigiosin** | **1,164,832** | **1,199,858** | **prodigiosin** | **Polyketide** | **100%** |
| thiopeptide | 1,768,432 | 1,794,876 | O-antigen | Saccharide | 14% |
| NRPS | 2,379,367 | 2,425,790 | microcin H47 | RiPP:Microcin | 20% |

| | | | | | |
|---|---|---|---|---|---|
| redox-cofactor | 2,499,844 | 2,522,006 | lankacidin C | NRP + Polyketide | 13% |
| NRPS | 2,549,550 | 2,598,060 | pyrronazol B | NRP + Polyketide | 9% |
| *Serratia marcescens* strain SmUNAM836 chromosome, complete sequence | | | | | |
| betalactone | 61,653 | 87,322 | - | - | - |
| **NRPS** | **429,252** | **473,901** | **prodigiosin** | **Polyketide** | **12%** |
| thiopeptide | 1,118,087 | 1,144,529 | O-antigen | Saccharide | 14% |
| NRPS | 1,734,087 | 1,780,797 | colicin V | RiPP | 1% |
| RRE-containing | 1,869,032 | 1,889,310 | lankacidin C | NRP + Polyketide | 13% |
| NRPS | 1,903,877 | 1,950,692 | - | - | - |
| NRPS | 2,614,272 | 2,663,290 | - | - | - |
| NRPS | 3,981,217 | 4,038,065 | rhizomide A / rhizomide B / rhizomide C | NRP | 100% |
| NRPS | 4,802,707 | 4,862,536 | vulnibactin | NRP | 18% |
| NRPS-like,hserlactone | 4,973,901 | 5,026,128 | - | - | - |
| *Serratia marcescens* strain Sys06 chromosome, complete genome | | | | | |
| NRPS | 310,758 | 369,305 | vulnibactin | NRP | 18% |
| betalactone | 745,030 | 770,699 | - | - | - |
| **NRPS,prodigiosin** | **1,135,249** | **1,196,122** | **prodigiosin** | **Polyketide** | **100%** |
| thiopeptide | 1,781,008 | 1,807,452 | O-antigen | Saccharide | 14% |
| NRPS | 2,463,253 | 2,508,769 | microcin H47 | RiPP:Microcin | 20% |
| redox-cofactor | 2,581,007 | 2,603,169 | lankacidin C | NRP + Polyketide | 13% |
| NRPS | 2,613,058 | 2,661,199 | - | - | - |
| NRPS | 3,327,405 | 3,403,931 | ravidomycin | Polyketide | 5% |
| NRPS | 4,881,352 | 4,925,290 | xantholipin | Polyketide | 4% |
| *Serratia marcescens* strain U36365 chromosome, complete genome | | | | | |
| NRPS | 288,656 | 329,856 | enterobactin | NRP | 12% |
| betalactone | 707,762 | 733,430 | - | - | - |

| | | | | | |
|---|---|---|---|---|---|
| RRE-containing | 929,883 | 949,788 | lankacidin C | NRP + Polyketide | 13% |
| **NRPS,prodigiosin** | **1,102,061** | **1,162,917** | **prodigiosin** | **Polyketide** | **100%** |
| thiopeptide | 1,745,141 | 1,771,583 | O-antigen | Saccharide | 14% |
| NRPS | 2,337,950 | 2,383,545 | microcin H47 | RiPP:Microcin | 20% |
| redox-cofactor | 2,464,738 | 2,486,900 | lankacidin C | NRP + Polyketide | 13% |
| NRPS | 2,495,912 | 2,543,838 | vanchrobactin | NRP | 20% |
| NRPS | 3,197,799 | 3,274,199 | ravidomycin | Polyketide | 5% |
| NRPS | 4,574,674 | 4,618,612 | xantholipin | Polyketide | 4% |
| *Serratia marcescens* 2020-O-9 DNA, complete genome | | | | | |
| NRPS | 263,474 | 322,000 | vulnibactin | NRP | 18% |
| NRPS-like,hserlactone | 435,964 | 487,457 | - | - | - |
| betalactone | 713,611 | 739,280 | - | - | - |
| **NRPS** | **1,129,447** | **1,174,122** | **prodigiosin** | **Polyketide** | **12%** |
| thiopeptide | 1,827,365 | 1,853,806 | O-antigen | Saccharide | 14% |
| NRPS | 2,453,865 | 2,500,576 | colicin V | RiPP | 1% |
| RRE-containing | 2,572,053 | 2,592,331 | lankacidin C | NRP + Polyketide | 13% |
| NRPS | 2,610,205 | 2,654,669 | - | - | - |
| NRPS | 3,343,672 | 3,392,693 | - | - | - |
| NRPS | 4,863,317 | 4,920,677 | xenotetrapeptide | NRP | 100% |
| *Serratia marcescens* AS-1 DNA, complete genome | | | | | |
| NRPS | 300,445 | 359,422 | vulnibactin | NRP | 18% |
| NRPS-like | 485,553 | 525,366 | - | - | - |
| betalactone | 768,048 | 793,718 | - | - | - |
| **prodigiosin** | **1,122,547** | **1,157,558** | **prodigiosin** | **Polyketide** | **100%** |
| thiopeptide | 1,727,800 | 1,754,244 | O-antigen | Saccharide | 14% |
| NRPS | 2,308,311 | 2,354,183 | microcin H47 | RiPP:Microcin | 20% |
| redox-cofactor | 2,429,962 | 2,452,124 | lankacidin C | NRP + Polyketide | 13% |

| | | | | | |
|---|---|---|---|---|---|
| NRPS | 2,480,922 | 2,527,585 | pyrronazol B | NRP + Polyketide | 9% |
| NRPS | 4,530,605 | 4,574,540 | xantholipin | Polyketide | 4% |
| *Serratia marcescens* ATCC 274 DNA, complete genome | | | | | |
| NRPS | 274,433 | 333,005 | vulnibactin | NRP | 12% |
| NRPS-like | 458,734 | 498,640 | - | - | - |
| betalactone | 719,523 | 745,192 | - | - | - |
| **prodigiosin** | **1,087,690** | **1,122,710** | **prodigiosin** | **Polyketide** | **100%** |
| thiopeptide | 1,732,825 | 1,759,268 | O-antigen | Saccharide | 14% |
| NRPS | 2,311,585 | 2,358,592 | microcin E492 | RiPP:Microcin | 12% |
| redox-cofactor | 2,433,927 | 2,456,089 | lankacidin C | NRP + Polyketide | 13% |
| NRPS | 2,484,591 | 2,531,445 | pyrronazol B | NRP + Polyketide | 9% |
| NRPS | 4,604,519 | 4,648,289 | xantholipin | Polyketide | 4% |
| *Serratia marcescens* isolate GN26 chromosome | | | | | |
| **NRPS** | **213,720** | **258,396** | **prodigiosin** | **Polyketide** | **12%** |
| thiopeptide | 896,329 | 922,771 | O-antigen | Saccharide | 14% |
| NRPS | 1,465,562 | 1,512,278 | colicin V | RiPP | 1% |
| RRE-containing | 1,583,452 | 1,603,730 | lankacidin C | NRP + Polyketide | 13% |
| NRPS | 1,618,612 | 1,664,234 | turnerbactin | NRP | 15% |
| NRPS | 2,320,263 | 2,369,375 | - | - | - |
| NRPS | 3,675,927 | 3,732,796 | xenotetrapeptide | NRP | 100% |
| NRPS,T1PKS | 3,966,368 | 4,020,760 | - | - | - |
| hserlactone | 4,275,438 | 4,296,112 | - | - | - |
| NRPS | 4,526,981 | 4,586,804 | vulnibactin | NRP | 18% |
| NRPS-like | 4,699,145 | 4,742,132 | - | - | - |
| betalactone | 4,971,369 | 4,997,038 | - | - | - |
| *Serratia marcescens* isolate PWN146_assembly genome assembly, chromosome: Chromosome | | | | | |
| betalactone | 60,892 | 86,559 | - | - | - |

| | | | | | |
|---|---|---|---|---|---|
| NRPS,T1PKS | 546,260 | 622,499 | olimycin A / olimycin B | Polyketide | 5% |
| thiopeptide | 1,196,198 | 1,222,640 | O-antigen | Saccharide | 14% |
| NRPS | 1,775,544 | 1,822,844 | microcin E492 | RiPP:Microcin | 18% |
| RRE-containing | 1,899,914 | 1,920,192 | lankacidin C | NRP + Polyketide | 13% |
| T1PKS,NRPS | 1,929,414 | 1,999,831 | pyrronazol B | NRP + Polyketide | 9% |
| T1PKS,NRPS | 2,596,947 | 2,660,035 | althiomycin | NRP | 100% |
| NRPS | 2,726,955 | 2,772,474 | - | - | - |
| siderophore | 3,951,781 | 3,963,640 | - | - | - |
| NRPS | 4,147,563 | 4,204,049 | xenotetrapeptide | NRP | 100% |
| NRPS | 5,033,555 | 5,093,384 | vulnibactin | NRP | 18% |
| RiPP-like | 5,200,901 | 5,213,138 | - | - | - |
| *Serratia marcescens* SM39 DNA, complete genome | | | | | |
| betalactone | 60,877 | 86,546 | - | - | - |
| **NRPS** | **476,718** | **521,393** | **prodigiosin** | **Polyketide** | **12%** |
| thiopeptide | 1,171,529 | 1,197,979 | O-antigen | Saccharide | 14% |
| NRPS | 1,764,120 | 1,810,831 | colicin V | RiPP | 1% |
| RRE-containing | 1,882,308 | 1,902,586 | lankacidin C | NRP + Polyketide | 13% |
| NRPS | 1,920,322 | 1,964,924 | - | - | - |
| NRPS | 2,609,074 | 2,658,095 | - | - | - |
| NRPS | 3,982,793 | 4,039,646 | xenotetrapeptide | NRP | 100% |
| NRPS | 4,835,798 | 4,895,615 | vulnibactin | NRP | 18% |
| NRPS-like,hserlactone | 5,008,228 | 5,060,300 | - | - | - |
| *Serratia marcescens* SMB2099 complete genome | | | | | |
| NRPS | 319,342 | 378,038 | vulnibactin | NRP | 18% |
| NRPS-like | 492,592 | 535,160 | - | - | - |
| betalactone | 787,697 | 813,366 | - | - | - |
| **NRPS** | **1,154,309** | **1,198,985** | **prodigiosin** | **Polyketide** | **12%** |

| | | | | | |
|---|---|---|---|---|---|
| thiopeptide | 1,804,409 | 1,830,851 | O-antigen | Saccharide | 14% |
| NRPS | 2,373,641 | 2,420,358 | colicin V | RiPP | 1% |
| RRE-containing | 2,491,533 | 2,511,811 | lankacidin C | NRP + Polyketide | 13% |
| NRPS | 2,526,716 | 2,572,914 | vanchrobactin | NRP | 20% |
| NRPS | 3,193,684 | 3,242,795 | - | - | - |
| NRPS | 4,593,237 | 4,650,089 | xenoamicin A / xenoamicin B | NRP:Cyclic depsipeptide | 20% |
| NRPS,T1PKS | 4,881,757 | 4,936,379 | - | - | - |
| *Serratia marcescens* strain 11/2010 chromosome, complete genome | | | | | |
| NRPS | 282,874 | 341,107 | vulnibactin | NRP | 18% |
| NRPS-like | 454,922 | 497,028 | - | - | - |
| betalactone | 701,219 | 726,888 | - | - | - |
| thiopeptide | 1,733,192 | 1,759,635 | O-antigen | Saccharide | 14% |
| RRE-containing | 2,533,651 | 2,553,929 | lankacidin C | NRP + Polyketide | 13% |
| NRPS | 2,574,600 | 2,622,335 | pyrronazol B | NRP + Polyketide | 9% |
| NRPS | 3,269,618 | 3,355,308 | - | - | - |
| lanthipeptide-class-i | 3,384,824 | 3,412,657 | yersiniabactin | NRP + Polyketide | 2% |
| NRPS,butyrolactone | 4,789,483 | 4,849,143 | xenotetrapeptide | NRP | 100% |
| *Serratia marcescens* strain 1140- chromosome, complete genome | | | | | |
| NRPS | 292,397 | 350,100 | vulnibactin | NRP | 18% |
| NRPS-like | 474,518 | 516,297 | - | - | - |
| betalactone | 772,671 | 798,340 | - | - | - |
| **NRPS** | **1,191,319** | **1,235,993** | **prodigiosin** | **Polyketide** | **12%** |
| thiopeptide | 1,833,431 | 1,859,872 | O-antigen | Saccharide | 14% |
| NRPS | 2,548,192 | 2,594,891 | colicin V | RiPP | 1% |
| RRE-containing | 2,679,562 | 2,699,840 | lankacidin C | NRP + Polyketide | 13% |
| NRPS | 2,720,029 | 2,765,172 | pyrronazol B | NRP + Polyketide | 9% |

| | | | | | |
|---|---|---|---|---|---|
| NRPS | 3,407,335 | 3,456,885 | lipopolysaccharide | Saccharide:Lipopolysaccharide | 5% |
| NRPS | 3,520,077 | 3,576,934 | gobichelin A / gobichelin B | NRP | 11% |
| RiPP-like | 3,918,624 | 3,931,170 | - | - | - |
| NRPS | 4,831,097 | 4,888,949 | rhizomide A / rhizomide B / rhizomide C | NRP | 100% |
